# Modulated Light Dependence of Growth, Flowering, and the Accumulation of Secondary Metabolites in Chilli

**DOI:** 10.3389/fpls.2022.801656

**Published:** 2022-03-22

**Authors:** Eva Darko, Kamirán A. Hamow, Tihana Marček, Mihály Dernovics, Mohamed Ahres, Gábor Galiba

**Affiliations:** ^1^Agricultural Institute, Centre for Agricultural Research, Martonvásár, Hungary; ^2^Faculty of Food Technology, Josip Juraj Strossmayer University of Osijek, Osijek, Croatia; ^3^Georgicon Faculty, Hungarian University of Agriculture and Life Sciences, Keszthely, Hungary

**Keywords:** capsaicine, flavonoids, LED lighting, secondary metabolites, chilli

## Abstract

Chili is widely used as a food additive and a flavouring and colouring agent and also has great importance in health preservation and therapy due to the abundant presence of many bioactive compounds, such as polyphenols, flavonoids, carotenoids, and capsaicinoids. Most of these secondary metabolites are strong antioxidants. In the present study, the effect of light intensity and spectral composition was studied on the growth, flowering, and yield of chilli together with the accumulation of secondary metabolites in the fruit. Two light intensities (300 and 500 μmol m^–2^ s^–1^) were applied in different spectral compositions. A broad white LED spectrum with and without FR application and with blue LED supplement was compared to blue and red LED lightings in different (80/20 and 95/5%) blue/red ratios. High light intensity increased the harvest index (fruit yield vs. biomass production) and reduced the flowering time of the plants. The amount of secondary metabolites in the fruit varied both by light intensity and spectral compositions; phenolic content and the radical scavenging activity were stimulated, whereas capsaicin accumulation was suppressed by blue light. The red colour of the fruit (provided by carotenoids) was inversely correlated with the absolute amount of blue, green, and far-red light. Based on the results, a schematic model was created, representing light-dependent metabolic changes in chilli. The results indicated that the accumulation of secondary metabolites could be modified by the adjustment of light intensity and spectral composition; however, different types of metabolites required different light environments.

## Introduction

Chilli is widely used as a food additive, a flavouring and colouring agent, and as a part of traditional medicine; it is used to treat, for instance, coughs, sore throat, rheumatism, and gastrointestinal ailments. It has great importance in preventing chronic diseases, such as diabetes and high cholesterol levels, cardiovascular or neurodegenerative diseases, and it provides protection against different types of cancer (Wahyuni et al., 2013). The beneficial therapeutic effects of the chilli fruit are mainly associated with the abundant presence of phytochemicals, such as carotenoids, flavonoids, polyphenols, and capsaicinoids. Most of these are strong antioxidants with anti-inflammatory, anticancer, and antimicrobial effects (Sarker et al., 2018c). They can act as immunomodulators (Jimenez-Garcia et al., 2013). While they can directly scavenge various free radicals, carotenoids give the attractive colours of the fruit, and flavonoids (such as quercetin and kaempferols) protect the low-density lipoprotein cholesterols from oxidation. They stimulate the enzymes involved in the detoxification of cancerogenic substrates and inhibit inflammations (Jimenez-Garcia et al., 2013).

Capsaicinoids, including trans-capsaicin (t-C), dihydrocapsaicin (DHC), nordihydrocapsaicin (n-DHC), homocapsaicin (h-C), and homodihydrocapsaicin (h-DHC) are responsible for the hotness (the pungency level) of the chilli and affect the cardiovascular and respiratory systems (Wahyuni et al., 2013). The pungency of capsaicinoidsis is usually expressed in Scoville heat units (SHU), indicating the highest dilution of a chilli fruit extract at which heat can be detected (Scoville, 1912). Nowadays, SHU is mainly determined by chromatographic methods, which are considered to be more reliable and accurate (Nwokem et al., 2010).

In chilli fruit, the amount of the main secondary metabolites shows enormous variations between the different cultivars, and it strongly depends on the maturity stage of the plants and the environmental conditions where the plants grow (Wahyuni et al., 2011; Hernández-Pérez et al., 2020). As summarised by Antonio et al. (2018), the capsaicinoid content of chilli varies from 28 to 200,000 μg of g dry fruit, providing 100–2,000,000 SHU, which enables chilli to be used for various purposes. For instance, moderate or low capsaicin content found in a cherry bomb type chilli is preferable for medical purposes due to low alkaloid toxicity (Acunha et al., 2017). The carotenoid, flavonoid, and polyphenol contents of the chilli fruit also show great variability (Wahyuni et al., 2011; Lemos et al., 2019). However, the high diversity in the metabolic profile made it a difficult task to understand the biochemical behaviour of plants grown under different environmental conditions.

Light is one of the most important environmental factors that determine the growth and development of plants. Through photosynthesis and different kinds of photoreceptors, light intensity and spectral composition affect biomass and yield formation through the modification of the primary and secondary metabolic pathways. The application of LED (light-emitting diode) technology in plant cultivation has accelerated the research on the effect of light fluence and wavelength on plant metabolism.

The influence of LEDs on growth and yield production has been widely investigated in many vegetable crops as summarised in several papers (Olle and Viršile, 2013; Darko et al., 2014; Loi et al., 2021). These studies have revealed that different light environments (including light intensity and spectral composition) are optimal for different kinds of crops, and that the use of a continuous wide spectrum composed of white, red, and blue LEDs is more suitable for plant cultivation than red and blue LEDs only (i.e., the lack of the green region) (Song et al., 2017; Liu et al., 2019; Naznin et al., 2019).

However, comparing the ample amount of data obtained from different species grown under different environmental conditions is difficult. The main light factors determining a given morphological or physiological parameter have not been identified in many species. Is it the absolute amount or the relative ratio of different light wavelengths that counts? Even less information is available about how the different kinds of LEDs affect the individual metabolic pathways—this is especially true for the production of secondary metabolites—in spite of the fact that LEDs provide unique possibilities for the targeted manipulation of plant metabolism (Darko et al., 2014). Only a few papers discuss the metabolic changes in chilli grown under LEDs. An early research studied the leaf and stem anatomy of Hungarian wax chilli (Schuerger et al., 1997). Later, changes in the primary (sugar, starch, and proteins) and secondary (fruit colour and the pungency level of capsaicinoids) metabolites were compared in chilli grown under monochromatic red or blue LEDs and under a 1:1 mixture of red and blue LEDs (Gangadhar et al., 2012). The highest biomass and yield were found under combined blue and red LEDs, and were accompanied by intense fruit colour. However, pure blue LEDs stimulated the accumulation of capsaicinoids. Recently, detailed metabolomic analysis focusing on capsaicinoids has been carried out on the fruit of a super-hot chilli grown in a greenhouse (under sunlight) supplemented with monochromatic red, blue, and red + blue (1:1) lightings for 5 h (Yap et al., 2021). Although the yield was the highest under sunlight (control), the additional blue LEDs significantly increased the accumulation of capsaicinoids, including C, DHC, n-DHC, h-C, and h-DHC (Yap et al., 2021). These results indicate that applying LED technology in the growth of chilli plants can be utilised for modifying the quality of the product, especially the secondary metabolite contents.

The aim of this research was to study the growth, yield, and the accumulation of several secondary metabolites (carotenoids, capsaicinoids, and phenolic compounds) and the antioxidant capacity of the fruit of chilli grown under pure artificial (LED) lighting conditions. The effects of blue and red LEDs were compared to different broad white spectrums with and without far-red application and with an increased blue region. Correlation analyses were performed to reveal the light factors affecting the production of these secondary metabolites.

## Materials and Methods

### Plant Materials and Growth Conditions

A cherry bomb type Hungarian chilli (*Capsicum annuum* cv. Kalocsai) was used in the experiments. The treatments were arranged following a completely randomised block design and repeated 3 times. Before reaching the eight-leaf stage, the plants were grown in 10-cm- × –10-cm- × –6.5-cm jiffy pots (Jiffy Group, Oslo, Norway) placed in streamline half-strength Hoagland solution under the same conditions; the temperature was between 22 and 25°C, and the light intensity was 250 ± 12 μmol m^–2^ s^–1^, provided by fluorescent lamps for 12 h per day. Afterward, the plants were transplanted into 5-L plastic pots (1 plant/pot) filled with a 2:1:1 (v/v/v/) mixture of garden soil, humus, and sand. About 72 pots were randomised into 6 groups, and the plants were grown under 6 different light regimens (designated regimen A-F) for 87 days (till maturity) at 16/8 h photoperiod, 22/25°C day/night temperature, and 70% humidity in growth chambers (PGV-36, ConvironEnv LTD, Winnipeg, MB, Canada). The light was provided by LED modules. Each LED module is composed of wide-spectrum LEDs and 4 types of narrow-bandwidth LEDs with dominant wavelengths of 420 and 448 nm (“blue LED”), 665 nm (“red LED”), and 750 nm (“far-red LED”), respectively. All of them could be controlled independently. In the present study, different LED-based light combinations (regimens) were tested; as basic spectrums, only the red and blue LEDs were used at moderate (300 μmol m^–2^ s^–1^) light intensity in two different red/blue proportions: 95/5% (regimen A) and 80/20% (regimen B). Since sunlight has a wide spectrum, another spectrum was designed, containing white, red, and blue LEDs in a proportion of 65:20:15 of blue:green:red regions, giving a total light intensity of 300 μmol m^–2^ s^–1^ (regimen C). Furthermore, to simulate sunrise and sunset (Kotilainen et al., 2020), the latter spectrum was also supplemented with 5% far-red light in the first and last hours of the light periods (Regimen D). To study whether the absolute or the relative amount of different spectral regions is important, this latter spectral combination was also used at a higher (500 μmol m^–2^ s^–1^) light intensity (Regimen E). Finally, the light regimen E was modified by increasing the proportion of the blue region by 5% in the middle of the light cycle for 4 h (Regimen F). The light spectrums and the programmes are presented in Supplementary Figure 1. In addition, the light intensities and the spectral characteristics of the light were integrated daily (DLI), as summarised in Table 1. This type of calculation was used for correlation analysis. During the growth period, the plants were rearranged regularly within the light regimens and watered every day. A nutrient solution containing 41 mg/L N; 7 mg/L P_2_O_5_; 21 mg/L K_2_O; 4 mg/L Mg; 5 mg/L Ca; and 1 mg/L B, Cu, Mn, Fe, and Zn was applied two times a week to ensure adequate water and nutrient supply.

**TABLE 1:** Spectral characteristics of light used in the experiments according to daily light integral (DLI), a red/blue ratio, and a proportion of different regions.

Light regimens	DLI (mol/day)					Red/Blue ratio	Red%	Green%	Blue%	Far-red%
	SUM	Blue region (400–500 nm)	Green region (500–600 nm)	Red region (600–700 nm)	Far-Red region (> 700 nm)					
A	17.28	0.86	0	16.42	0	19	95	0	5	0
B	17.28	3.46	0	13.82	0	4.0	80	0	20	0
C	17.28	2.59	3.46	11.23	0	4.3	65	20	15	0
D	17.28	2.59	3.46	11.12	0.11	4.3	65	20	15	0.6
E	28.8	4.32	5.76	18.53	0.18	4.3	65	20	15	0.6
F	28.8	4.72	5.76	18.53	0.18	3.9	64	20	16	0.6

*Light regimens used: Regimen A, red and blue LEDs were used with a 95:5 ratio at moderate (300 μmol m^–2^ s^–1^) light intensity; Regimen B, utilisation of red and blue LEDs with an 80:20 ratio at moderate (300 μmol m^–2^ s^–1^) light intensity; Regimen C, a wide spectrum (containing white, red, and blue LEDs) in an average proportion of 65:20:15 of red:green:blue at moderate (300 μmol m^–2^ s^–1^) light intensity: Regimen D, a wide spectrum supplemented with far-red application in an average proportion of 65:20:15:0.6 of red:green:blue:far-red at moderate (300 μmol m^–2^ s^–1^) light intensity; Regimen E, a wide spectrum supplemented with far-red application in an average proportion of 65:20:15:0.6 of red:green:blue:far-red at high (500 μmol m^–2^ s^–1^) light intensity; Regimen F, a wide spectrum supplemented with far-red and blue light application in an average proportion of 64:20:16:0.6 of red:green:blue:far-red at high (500 μmol m^–2^ s^–1^) light intensity. The total photosynthetic photon flux density (PPFD) integrated between 400 and 750 nm and the spectral distribution were determined by PG200N Spectral PAR Meter (Uprtek Europe Dl Technology GmbH, Aachen, Germany), where the blue region was integrated between 400 and 500 nm, the green region between 500 and 600 nm, the red region between 600 and 700 nm, and the far-red region between 700 and 750 nm.*

### Morphological Parameters

At harvest (87 days after the start of the light treatments), several morphological parameters were determined, such as the aboveground plant height and mass (with and without yield) and the amount and weight of the fruit. In the present study, the aboveground plant mass without yield is designated as green mass, which includes the weight of the shoot and leaves. The number of flowers was counted during the flowering period (16–46 days after the light treatments). Harvest index (HI) was calculated as the ratio of the fruit yield and total aboveground biomass production of each plant. The morphological parameters were determined from 10 plants per light treatment.

### Metabolomics Analysis

For the metabolomics analyses, the ripe fruit was collected from each plant. The fruit was frozen in liquid nitrogen and kept at −80°C until preparation. Five samples were prepared per light regimen. Each sample consisted of 10 pieces of fruit collected from two plants (5 pieces of fruit per plant). During the extraction, the samples were homogenised using a blender as required for the analytical methods.

The total carotenoid content was determined from fruit pericarps according to the protocol described by Acunha et al. (2017). For each sample, 0.5-g fruit extract was homogenised with 10-ml solvent composed of hexane/methanol/acetone/toluene at 10:6:7:7 for 1 h in the dark. Next, carotenoids were transferred to the hexane (10 ml) phase, and their amount was measured spectrophotometrically, using a UV-visible spectrophotometer (160A, Shimadzu Corp., Kyoto, Japan).

Total phenolic content was determined according to Gomes et al. (2021). The samples (0.5-g fruit pericarps for each) were extracted in methanol (1:10), and the phenolic content was measured spectrophotometrically based on its reaction with Folin-Ciocalteu reagent. Gallic acid standard was used for the calibration curve, and the results were expressed in gallic acid (Merck-Sigma group, Darmstadt, Germany) equivalents (mg/g fresh mass).

The antioxidant activity of the fruit was determined by a DPPH (2,2-diphenyl-1-picrylhydrazyl) free radical assay according to Oney-Montalvo et al. (2020), and the radical scavenging activity was calculated in DPPH% reduction determined after 30-min reaction time.

The amount of capsaicinoids and phenolic composition were determined by the use of a Waters Acquity I-class Ultra performance liquid chromatography (UPLC) system equipped with a PDA detectors, which was coupled to either a Xevo TQ-XS Triple Quadrupole Mass Spectrometer (Waters Corp.; Milford, MA, United States) or a Vion IMS-QTOF-MS (Waters). The full protocols, including the preparation of samples, separation, identification, and the quantification of compounds, are detailed in Supplementary Material (Worksheets a–c); here, only the main parameters are given.

The extraction of capsaicinoids was carried out according to the Hungarian Standard Method “MSZ9681-4:2002.” Briefly, homogenised fruit samples (0.5-g dry mass) were extracted with 50-ml methanol in two steps by ultrasonication. The extracts were combined, centrifuged (4,000 × g for 15 min), and the supernatants were filtered through 0.22-μm PTFE syringe filters and were diluted 20-fold prior to analysis. The separation of capsaicinoids was achieved on a Supelco Core Phenyl-Hexyl analytical column (2.7 μm; 4.6 mm × 150 mm; Merck-Sigma group) under water: acetonitrile gradient elution. A Xevo TQ-XS MS detector equipped with a Unispray source was used in multiple reaction monitoring (MRM) and a positive ion mode. Quantification of the compounds was based on the characteristic fragment of m/z 137 of the [M + H]^+^ parent masses of capsaicinoids. A detailed description is found in Supplementary Material (Worksheet a).

The extraction and chromatographic analyses of phenolic compounds were carried out according to Vrhovsek et al. (2012) and Pál et al. (2019) with slight modifications. Homogenised fruit pericarps (0.5-g FW) were spiked with 50 ng [^2^H_6_](+)-*cis-, trans*-abscisic acid (OlChemIms.r.o. Olomouc, Czech Republic), serving as an internal standard before the extraction with 2-ml- × –2.5-ml methanol: water (2:1 v/v%). After centrifugation (at 14,000 × *g;* 4°C; for 10 min), 2.5 ml of n-hexane was added to the supernatants to remove carotenoids. Then, the phases were separated by centrifugation and the methanol: water phase was filtered through 0.22-μm PTFE syringe filters prior to analysis. Separation of phenolic compounds was achieved on an HSS T3 column (1.8 μm; 100 mm × 2.1 mm; Waters) under water: acetonitrile gradient elution (details to be found in Supplementary Material, Worksheet b). Xevo TQ-XS MS was utilised in MRM mode, and the respective MRM transitions used for quantification are listed in Supplementary Material (Worksheet b). Furthermore, identification of major phenolic analytes detected by PDA at λ = 330 nm, which were uncovered by the MRM methods, was achieved with the UPLC-Vion IMS-QTOF-MS setup according to Jeong et al. (2011), Morales-Soto et al. (2013), Kelebek et al. (2020), Mara de Menezes Epifanio et al. (2020), and Guclu et al. (2021) (for instrumental details, see Supplementary Materials, Worksheet c). Quantification of these analytes was carried out at λ = 330 nm against quercetin 3-rutinoside (rutin) reference material (Merck-Sigma group). Furthermore, several compounds possessed considerably higher abundance in the function of experimental setups on the basis of UV detection at λ = 330 nm, but they could not be unambiguously identified (referred to as NI1-20). However, additional data could be provided on the basis of their UPLC-MS characteristics (retention time, tentative elemental composition, MS/MS acquisitions, etc.) in Supplementary Material (Worksheet c), and HR-MS figures (presented in the Supplementary PDF File).

### Statistics

For each light regimen, the data presented in the tables and the figures derived from triplicate experiments involve at least 10 or 5 biological replicates of the morphological and analytical investigations, respectively. The SPSS 22 statistical programme and Tukey’s *post-hoc* test were used to determine differences between the light treatments. Different letters indicate significant differences at the *p* < 0.05 level. Spearman’s rank order correlation coefficients (at the significance level *p* < 0.05) were calculated with the Statistica 13.5 software (TIBCO Software Inc., Palo Alto, CA, United States) in order to determine the main light factors affecting the physiological and metabolic parameters measured. The value -1 represents negative (inverse) correlation, 1 indicates positive (direct) correlation, whereas the values close to zero indicate no correlation.

## Results

### Plant Growth, Flowering, and Yield Production

Plant height and green mass were measured to characterise the growth of chilli under different light intensities and spectral compositions (Table 2). The greatest plant height was measured when the plants were grown under blue and red light with 95/5 red/blue proportion at 300 μmol m^–2^ s^–1^ light intensity (light Regimen A), whereas the lowest values were detected in those plants which were grown at high light intensities with complete spectral composition (light Regimens E and F). Surprisingly, high light intensity (light Regimens E and F) resulted in the lowest green mass (biomass without the fruit); meanwhile, the yield was the highest, which was reflected in the high harvest index (Table 2). In contrast, the red and blue LEDs with a 95:5 ratio (light Regimen A) provided the highest green mass, which was accompanied with the lowest fruit yield, resulting in the lowest harvest index (Table 2).

**TABLE 2:** Plant morphology and biomass production of chilli grown under different light regimens (A–F).

Light regimens	Plant height (cm)	Greenmass (g)	No. of fruit per plant	Yield (g) of fruit per plant	Average fruit mass (g/pc.)	HI (yield/biomass)
A	61.5 ± 3.0a	114.7 ± 12.0a	8.9 ± 1.3a	54.4 ± 4.4b	6.11 ± 1.14*c*	0.32 ± 0.04b
B	59.4 ± 3.2a	112.3 ± 12.8a	8.6 ± 1.3ab	63.4 ± 5.3ab	7.37 ± 1.19*bc*	0.35 ± 0.05ab
C	58.8 ± 2.2a	111.6 ± 11.2a	8.4 ± 1.3ab	67.0 ± 5.6a	8.00 ± 1.24a	0.38 ± 0.05ab
D	58.0 ± 2.6ab	102.3 ± 12.4ab	7.4 ± 1.1*b*	59.2 ± 5.7ab	7.96 ± 1.25a	0.37 ± 0.05ab
E	54.4 ± 2.1*bc*	92.9 ± 10.5b	9.1 ± 1.2a	67.4 ± 5.1a	7.43 ± 1.12ab	0.42 ± 0.04a
F	51.1 ± 2.7*c*	87.8 ± 10.8b	9.0 ± 1.2a	66.1 ± 5.3a	7.34 ± 1.11ab	0.43 ± 0.04a

*Green mass represents the plant mass without yield mass. HI: harvest index calculated as the ratio of fruit yield and total above-ground biomass. Light conditions used: A, application of red and blue LEDs with a 95:5 ratio at moderate (300 μmol m^–2^ s^–1^) light intensity; B, utilisation of red and blue LEDs with an 80:20 ratio at (300 μmol m^–2^ s^–1^) light intensity; C, a wide spectrum with an average proportion of 65:20:15 of red:green:blue at moderate (300 μmol m^–2^ s^–1^) light intensity: D, A wide spectrum supplemented with far-red application in an average proportion of 65:20:15:0.6 of red:green:blue:far-red at moderate (300 μmol m^–2^ s^–1^) light intensity; E, a wide spectrum supplemented with far-red application in an average proportion of 65:20:15:0.6 of red:green:blue:far-red at high (500 μmol m^–2^ s^–1^) light intensity; F, a wide spectrum supplemented with far-red and blue light application in an average proportion of 64:20:16:0.6 of red:green:blue:far-red at high (500 μmol m^–2^ s^–1^) light intensity. Values indicated with different letters are significantly different at p < 0.05.*

Considering the data of yield, the fruit mass per plant and the average fruit mass together (Table 2), it appeared that high light intensity with complete spectral composition (light Regimens E and F) provided large amount of fruit with medium-average fruit mass. The similar spectral composition at moderate light intensity (light Regimens C and D) provided less fruit combined with the highest average fruit mass. When only blue and red LEDs with high proportion of blue light (light Regimen B) were applied, the plants developed large amount of fruit with low-average fruit mass (Table 2). The average fruit weight was the lowest when the plants were grown under blue and red light with 95/5 red/blue proportion at moderate light intensity (light Regimen A).

However, the pairwise comparison of growth parameters (i.e., plant height and green mass) showed significant differences between light Regimens A and B, C and D, and E and F, indicating that plant height and green mass were mainly affected by light intensity. The adverse effect of a high-red proportion applied in light Regimen A was detected in the lowest harvest index and in the average fruit mass. On the other hand, the application of far-red light at the beginning and at end of the light period, as well as the addition of blue light in the middle of the light period, all appeared ineffective.

**FIGURE 1:**
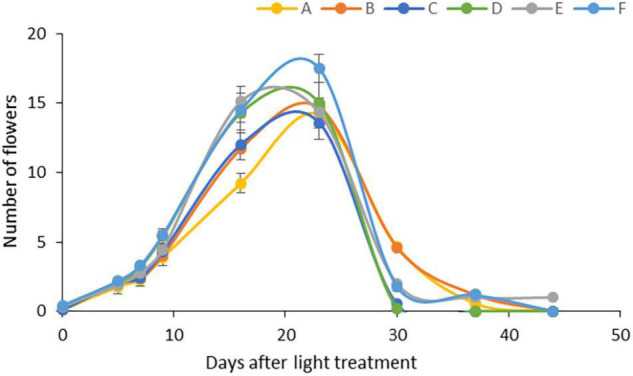
Flowering under different light regimens: **(A)** Application of red and blue LEDs with a 95:5 ratio at moderate (300 μmol m^–2^ s^–1^) light intensity; **(B)** utilisation of red and blue LEDs with a 80:20 ratio at (300 μmol m^–2^ s^–1^) light intensity; **(C)** a wide spectrum with an average proportion of 65:20:15 of red:green:blue at moderate (300 μmol m^–2^ s^–1^) light intensity: **(D)** a wide spectrum supplemented with far-red application in an average proportion of 65:20:15:0.6 of red:green:blue:far-red at moderate (300 μmol m^–2^ s^–1^) light intensity; **(E)** a wide spectrum supplemented with far-red application in an average proportion of 65:20:15:0.6 of red:green:blue:far-red at high (500 μmol m^–2^ s^–1^) light intensity; **(F)** a wide spectrum supplemented with far-red and blue light application in an average proportion of 64:20:16:0.6 of red:green:blue:far-red at high (500 μmol m^–2^ s^–1^) light intensity.

The light conditions modified the flowering period (Figure 1). The average number of flowers was similar under all light regimens, but high light intensity (light Regimens E and F) accelerated flowering, followed by the far-red application at moderate light intensity (light Regimen D). When only blue and red LEDs were used, the high proportion (20%) of blue light (light Regimen B) prolonged the flowering period, while the high proportion (95%) of red light (light Regimen A) delayed it.

### Light-Induced Metabolomic Changes in the Fruit

Capsaicinoids are one of the most important components of chilli. Capsaicinoid content, including the amount of trans-capsaicin (t-C), dihydrocapsaicin (DHC), nordihydrocapsaicin (n-DHC), homocapsaicin (h-C), and homodihydrocapsaicin (h-DHC), varied significantly under different light environments (Table 3). Surprisingly, the total amount of capsaicinoids was reduced in the fruit of the plants grown under high light intensity (light Regimens E and F), as well as under moderate light intensity with a high proportion of blue light (light Regimen B) as compared to other spectral combinations, where the amount of blue light was lower (Table 1). The application of FR light did not modify significantly the amount of capsaicinoids either at moderate or at high light intensity, according to the pairwise comparisons of light Regimens C and D, and E and F, respectively. These light-induced differences were observed in most of the capsaicinoid components, providing 40–83% maximal deviations, depending on the components.

**TABLE 3:** The amount of capsaicinoids (mg/kg DW) and the pungency level Scoville Heat Unit (SHU) value of chilli fruits: trans-capsaicin (t-C), dihydrocapsaicin (DHC), nordihydrocapsaicin (n-DHC), homocapsaicin (h-C); homodihydrocapsaicin (h-DHC), and the values of SHU.

Light regimens	Total capsaicinoids	t-C	DHC	n-DHC	h-DHC	h-C	SHU value
A	1,216 ± 79a	624 ± 51*ab*	440 ± 26a	72 ± 5.1a	52 ± 4.8a	22 ± 2.5a	18,303 ± 1,174a
B	887 ± 73b	462 ± 39c	316 ± 33b	52 ± 5.8b	38 ± 3.8b	15 ± 1.6b	13,373 ± 1,094b
C	1,249 ± 70a	672 ± 35a	428 ± 27a	74 ± 4.7a	50 ± 4.4a	20 ± 1.3a	18,868 ± 1,043a
D	1,183 ± 82a	598 ± 42b	435 ± 38a	75 ± 6.8a	51 ± 2.5a	19 ± 1.2a	17,799 ± 1,240a
E	928 ± 151b	518 ± 66bc	301 ± 60b	55 ± 10.2bc	36 ± 9.7b	15 ± 3.2b	14,030 ± 1,992b
F	859 ± 65b	487 ± 37c	282 ± 25b	41 ± 3.7c	31 ± 2.9b	16 ± 1.8b	13,072 ± 984b

*Light conditions used: A, application of red and blue LEDs with a 95:5 ratio at moderate (300 μmol m^–2^ s^–1^) light intensity; B, utilisation of red and blue LEDs with a 80:20 ratio at (300 μmol m^–2^ s^–1^) light intensity; C, a wide spectrum with an average proportion of 65:20:15 of red:green:blue at moderate (300 μmol m^–2^ s^–1^) light intensity: D, a wide spectrum supplemented with far-red application in an average proportion of 65:20:15:0.6 of red:green:blue:far-red at moderate (300 μmol m^–2^ s^–1^) light intensity; E, a wide spectrum supplemented with far-red application in an average proportion of 65:20:15:0.6 of red:green:blue:far-red at high (500 μmol m^–2^ s^–1^) light intensity; F, a wide spectrum supplemented with far-red and blue light application in an average proportion of 64:20:16:0.6 of red:green:blue:far-red at high (500 μmol m^–2^ s^–1^) light intensity. Values indicated with different letters are significantly different at p < 0.05.*

The t-C and DHC were the dominant capsaicinoids, reaching 85–90% within the total amount. The n-DHC and h-DHC represented about 5% of the total capsaicinoid content, whereas h-C was about 1–2%. When the relative proportions of each component were compared, the relative amount of t-C and DHC changed inversely with light treatment. Namely, in those cases where the total capsaicinoid content was high (under light Regimens A, C, and D), the relative amount of t-C (t-C/DHC ratio) was lower (ranged between 1.1 and 1.17) than in those cases where the total amount of capsaicinoids was low (under light Regimens B, E, and F) (Table 3). The h-DHC changed similarly to DHC, whereas the relative proportion of other components (n-DHC and h-C) did not show significant changes. Accordingly, the SHU indices (which indicate the most characteristic feature of chilli fruit, the hotness, and the spicy flavour) ranged between 13,000 and 19,000 (Table 3). Higher values were observed under light Regimens A, C, and D, while lower values were detected under light Regimens B, E, and F like in the case of t-C and DHC values. The former group (light Regimens A, C, and D) contained low absolute amount of blue light, while the light Regimens B, E, and F emitted higher absolute amount of blue light (Table 1). The SHU values imply a moderate pungency level; however, they are significantly higher than that of a typical cherrybomb fruit, whose SHU index generally ranges between 2,500 and 5,000.

**FIGURE 2:**
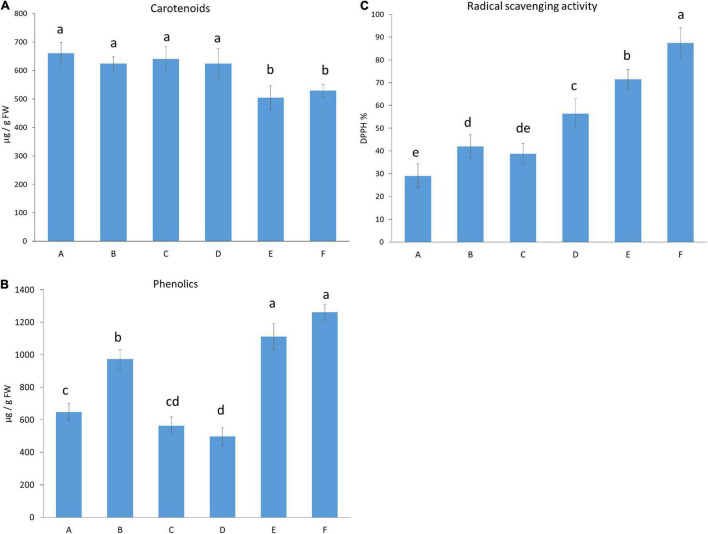
Carotenoid **(A)** and total phenolic content **(B)**, and an antioxidant capacity of the fruit **(C)**. Light conditions used: **(A)** Application of red and blue LEDs with a 95:5 ratio at moderate (300 μmol m^–2^ s^–1^) light intensity; **(B)**, utilisation of red and blue LEDs with an 80:20 ratio at (300 μmol m^–2^ s^–1^) light intensity; **(C)** a wide spectrum with an average proportion of 65:20:15 of red:green:blue at moderate (300 μmol m^–2^ s^–1^) light intensity: **(D)** a wide spectrum supplemented with far-red application in an average proportion of 65:20:15:0.6 of red:green:blue:far-red at moderate (300 μmol m^–2^ s^–1^) light intensity; **(E)**, a wide spectrum supplemented with far-red application in an average proportion of 65:20:15:0.6 of red:green:blue:far-red at high (500 μmol m^–2^ s^–1^) light intensity; **(F)** a wide spectrum supplemented with far-red and blue light application in an average proportion of 64:20:16:0.6 of red:green:blue:far-red at high (500 μmol m^–2^ s^–1^) light intensity.

The total amounts of carotenoids and phenolics and the antioxidant scavenging activity of the fruit were determined (Figure 2). The carotenoid content, responsible for fruit colours, was higher when the plants were grown in moderate light intensity (under light Regimens A, B, C, and D) compared to those fruits that were grown under high light intensity (light Regimens E and F) (Figure 2A), indicating that amongst the light conditions used, light intensity was the most dominant light factor that influenced carotenoids. Inversely, the highest amount of phenolic content was detected under high light intensity (light Regimens E and F), followed first by moderate light intensity with a high proportion of blue light (light Regimen B), and then by Regimens A and C. The lowest value was measured when a wide spectrum was used with far-red application under moderate light intensity (light Regimen D) (Figure 2B). The antioxidant capacity was also high at high light intensity (light Regimens E and F), especially when additional blue light was applied (Regimen F), followed by light Regimens D, B, and C (Figure 2C). The lowest antioxidant capacity was found when blue and red LEDs with a high proportion of red light were used (light Regimen A). The pairwise comparison of the radical scavenging activity and phenolics showed that both the light intensity and the spectral composition influenced the production of these metabolites.

**FIGURE 3:**
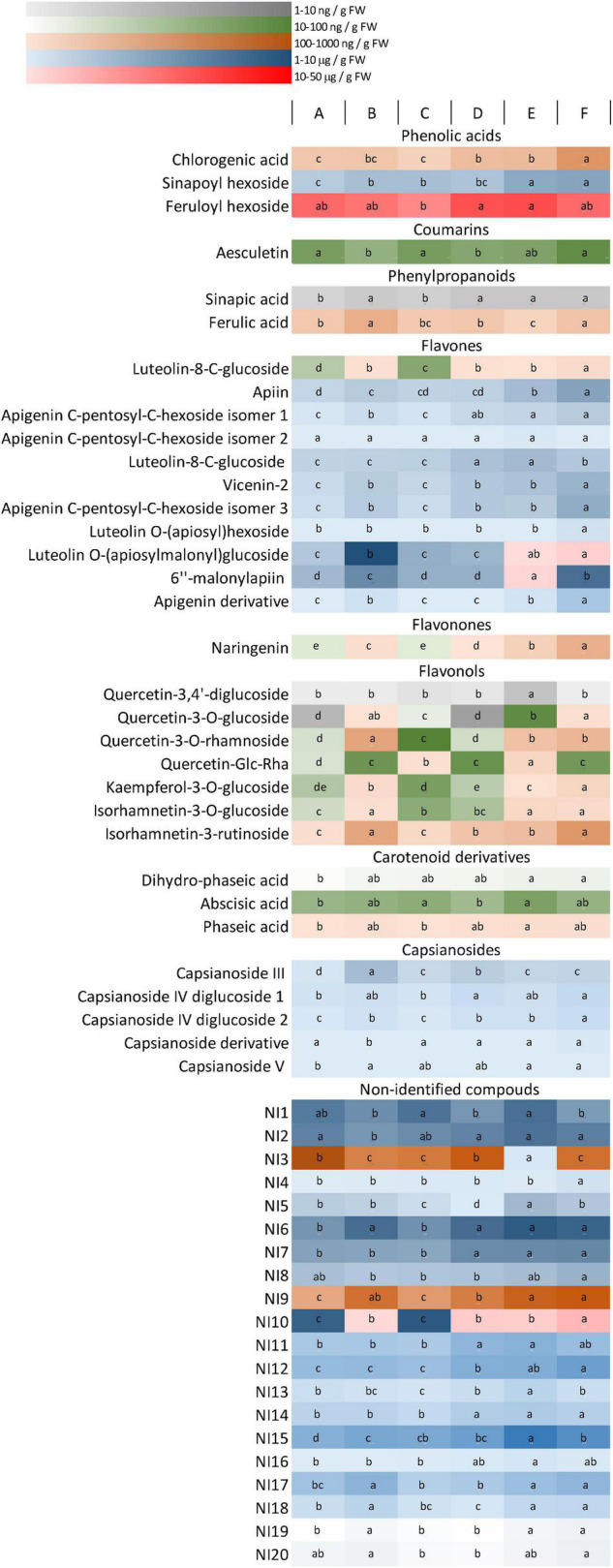
A heat map representing the accumulation of phenolic compounds in the fruit. Different colours represent different concentration ranges. Differences between the values (within the rows) are indicated by different letters. The significance was determined at the *p* < 0.05 level, using Tukey’ s *post-hoc* test. Light conditions used: **(A)** application of red and blue LEDs with a 95:5 ratio at moderate (300 μmol m^–2^ s^–1^) light intensity; **(B)** utilisation of red and blue LEDs with an 80:20 ratio at (300 μmol m^–2^ s^–1^) light intensity; **(C)** a wide spectrum with an average proportion of 65:20:15 of red:green:blue at moderate (300 μmol m^–2^ s^–1^) light intensity: **(D)** a wide spectrum supplemented with far-red application in an average proportion of 65:20:15:0.6 of red:green:blue:far-red at moderate (300 μmol m^–2^ s^–1^) light intensity; **(E)** a wide spectrum supplemented with far-red application in an average proportion of 65:20:15:0.6 of red:green:blue:far-red at high (500 μmol m^–2^ s^–1^) light intensity; **(F)** a wide spectrum supplemented with far-red and blue light application in an average proportion of 64:20:16:0.6 of red:green:blue:far-red at high (500 μmol m^–2^ s^–1^) light intensity.

Light dependence of phenolic compounds was determined by UPLC-MS analyses. Mainly, phenolic acids, coumarins, phenylpropanoids, and flavonoids, including flavones, flavonones, and flavonols, were identified in the fruit extracts. In addition, some other non-identified compounds (NI1-20) were also detected. Since they also showed light-dependence variation, the non-identified compounds (quantified in rutin equivalent) were also included in the figures. The amounts of the metabolites were indicated in Supplementary Table 1, and their light-induced changes were presented in Figure 3. Chilli fruit contains large amounts of flavones, capsianosides, precursors of phenylpropanoids and also, many non-identified compounds (NI1, 2, 3, 9, and 10) together with smaller amounts of flavonones, flavonols, and coumarins. Several metabolites showed light-dependent variations. Significant light-induced differences (more than 200% of the lowest value) were found for chlorogenic acid, ferulic acid, most flavones, flavonones, and flavonols, as well as for capsianoside III and non-identified compounds (NI) 5, 9, 10, and 19. These compounds make up 44% of all compounds and 65% of the total amounts (in μg/g FW). The light regimen induced moderate changes (ranging between 150 and 200%) for sinapic acid and its hexoside, dihydro-phaseic acid, capsianoside IV diglucoside 2, capsianoside V, and some minor luteolin and apigenin sugar derivatives, together with many non-identified compounds, including NI3, 7, 11–13, 15, 17, 18, and 20. These make up 30% of the total compounds and 27% of the total amount. About 26% of all compounds showed slight (but statistically significant) variation under different light environments, and they make up only 8% of the total amount. These compounds are feruloylhexoside, aesculetin, abscisic acid derived from carotenoids, phaseic acid, capsianoside IV diglucoside 1 casianoside derivative and several non-identified compounds, such as NI1, 2, 4, 7, 8, 14, and 16. These results indicate that the presence of phenolic compounds strongly depends on the light conditions. According to pairwise comparisons between light regimens A and B, C and D, and E and F), the blue light and far-red light caused significant changes in the amount of main phenolic compounds.

### Light Factors Affecting the Growth and Production of Various Metabolites

**FIGURE 4:**
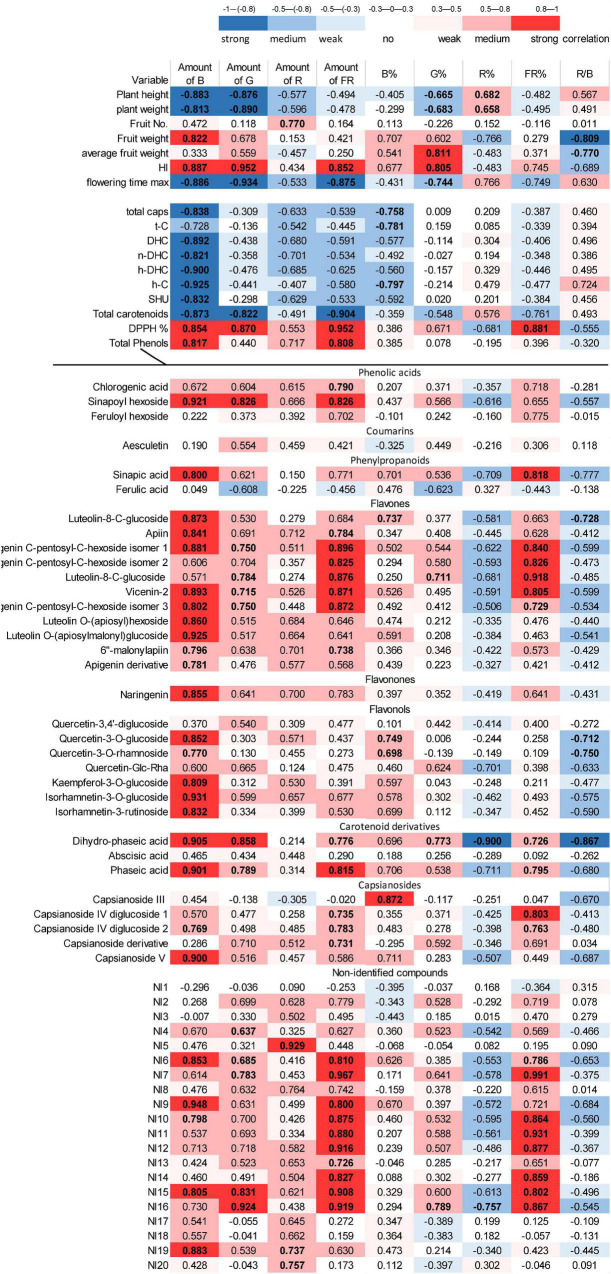
Correlation analysis between the light conditions and all studied parameters of chilli. The absolute and relative amounts of different light regions were integrated daily (DLI) and used as indicated in Table 1. The blue colour indicates negative (inverse), whereas red colour represents positive (direct) correlations, and more intense colour shows higher correlation. The “r” values are also presented. The bold values show the significance level at the *p* < 0.05 level.

According to the data, it seemed that, instead of specific light conditions used in the present experiments, the overall daily light, indeed, evoked the changes in the growth and metabolic changes in the fruits. Therefore, a correlation analysis was performed between the light conditions used (Table 1) and the morphological and metabolomics values in order to reveal whether the absolute amount or the relative ratios of specific spectral regions affect the given parameters (Figure 4).

Plant height and green mass were related to the absolute amount of blue and green spectral regions. In addition, the percentage of green (G%) and red light (R%) moderately affected these parameters. The blue and green regions correlated negatively with growth parameters (e.g., as blue and green regions increase, the plant height and green mass decrease). The percentage of red light (R%) affected these parameters in positive direction. Yield (fruit mass per plant) was mainly affected by the amount of blue light, but the red/blue ratio appeared as important as the absolute amount of blue light. Yield was affected positively by blue light and negatively by the R/B ratio. Interestingly, the average fruit weight was affected by the G% and moderately by the R/B ratio. The harvest index strongly depended on the absolute amounts of blue, green, and far-red regions together with G%. Similar results were found for flowering time in an inverse tendency, indicating that the higher amount of blue, green, and far-red light induces early flowering of plants. These results demonstrated that, besides blue and red regions– in which the absolute amount of blue light and the R% or the R/B ratio may be effective—the green region also played an important role in plant growth, flowering, and yield production.

The accumulation of metabolites in the fruit also showed light-dependent variations. The absolute amount of blue light was the main factor affecting the accumulation of capsaicinoids, including all compounds, such as t-C, DHC, n-DHC, h-DHC, and h-C, and the SHU in this chilli. The carotenoid content was affected by the absolute amount of blue, green, and far-red regions. Both groups of metabolites showed inverse correlation with the light factors, i.e., a higher amount of light yielded a lower amount of metabolites. In contrast, total phenolics showed direct (positive) correlation with the absolute amount of the blue and far-red regions. The antioxidant capacity of the fruit was correlated mainly with the amount of blue, green, and far-red regions and with FR%.

Detailed analysis of phenolic compounds also revealed strong light-dependent variations. Flavonoid (flavones, flavonones, and flavonols) content was mainly affected by absolute amount of the blue light but especially, in case of flavones (e.g., apigenin-sugar derivatives), far-red light also seemed to be important. A moderate effect of the green region could also be detected. Light did not significantly modify the amount of chlorogenic acid, ferulic acid, feruloylhexoside, and coumarin-type aesculetin. Similarly, the amount of abscisic acid did not change significantly, but its derivatives, phaseic, and dihydrophaseic acids were positively affected by blue, green, and far-red light and inversely by R% and the R/B proportion. The light dependency of many, approximately half of the non-identified metabolites (NI6, 7, 9–12 and 14–16), resembles that of flavones and differs from NI5, 8, 13, 17, 18, and 20. These results indicate that metabolic processes can be modified by the spectral composition of the light.

## Discussion

Plant cultivation under artificial light in indoor cultivation systems requires optimisation of light conditions. However, little to decide information has been available on specific light factors involved whether the absolute or the relative proportions of different spectral regions influence the growth and development of plants and their metabolic processes. Harmonisation of red and blue lighting to natural light environment can be a strategy for optimisation of artificial light environment. In the present study, the basic concept was to complete the commonly used red and blue spectra with white light, and then with FR treatment applied in the morning and evening periods (simulating the sunrise and sunset periods) at moderate and high light intensities. In addition, the energy level of light was increased in noon periods by adding supplemental blue lighting.

According to the results, the pairwise comparison of different light regimens, namely the different blue and red ratios (light Regimens A and B), and the application of white LED with or without far-red treatments (light Regimens C and D), together with the changes of light intensities (light Regimens E and F), showed that the pairwise changes were less important; indeed, the overall daily effect turned out to be influencing. For instance, in comparison with the light Regimens E and F, the extra energy applied during the middle of the growth period (light Regimen F) did not cause significant changes; as a whole, this modification triggered small changes in the daily light integral. In contrast, light regimens containing a high amount or a high proportion of blue light (light Regimens E, F, and B) induced similar changes in growth (e.g., decrease in plant height) and metabolism (e.g., inducing the accumulation of phenolics) in spite of the fact that they differed both in light intensities and spectral composition. Therefore, in the future, more attention should be given to the importance of the absolute amount and the relative proportion of different spectral regions.

In the present investigation, the morphological parameters mainly affected by light intensity and spectral combination were plant height, flowering time, and yield production. The absolute amount of blue light had a negative effect on plant height and green mass production and a positive effect on fruit mass and harvest indices. Apparently, this is a contradiction. However, as demonstrated by several authors previously, an increase in light intensity resulted in an increase of biomass and yield only up to a critical threshold, whereas it decreased plant height in many crops, including tomato seedlings (Huber et al., 2021), wheat (Monostori et al., 2018), and chilli (Masabni et al., 2016). The details also showed that the utilised photosynthetic energy was distributed unequally between green mass and yield production. For instance, doubling the light intensity from 250 to 500 μmol m^–2^ s^–1^ resulted in a 1.25-fold increase of green mass and a 1.5-fold increase of yield in wheat (Monostori et al., 2018). In chilli plants, growth at high light intensity did not result in elevated shoot mass as compared to moderate light intensity, whereas yield increased (Masabni et al., 2016). These results are in accordance with the present observations, indicating that when more energy is absorbed by the photosynthetic pigments, the energy can be more efficiently converted into yield production. In addition, the present results also demonstrate that spectral composition also affected green mass and yield production, resulting in the highest green mass and the lowest yield at low light dominated by the red spectral region. Similar results were found in tomato plants grown at different red/blue ratios, where the biomass production was the highest at a high proportion of red light, whereas the highest fruit production was obtained at a high proportion of blue light (Deram et al., 2014). It appeared that a low amount of blue light (at low light intensity with red dominant spectra) stimulates green mass production, whereas a sufficient amount of blue light can improve yield.

Stem elongation is controlled by many photoreceptors, including PhyA responsible for far-red light signalling, PhyB regulating red light signalling, and phototropins and cryptochroms responding to blue light regions (Wang and Deng, 2004; Zhiyu et al., 2007; Hu and Zhao, 2016; Legris et al., 2019; Pierik and Ballaré, 2021). The light-induced regulation mechanisms depend both on the fluence and the spectral composition of light (Wang, 2005). Under the light conditions used in the present experiments, plant height was mainly determined by absolute amounts of blue and green light. Since leaf numbers did not change significantly (data not shown), the blue and green lights mainly affected the stem elongation. The blue light-induced inhibition of stem elongation was also demonstrated in bell pepper (Claypool and Lieth, 2020), tomato seedlings (Deram et al., 2014), and in chrysanthemum (Kim et al., 2004). The inhibitory effect of blue light on stem elongation was also presented inversely by Runkle and Heins (2001), who reported that environments deficient in blue light promoted internode elongation in several long-day plants as compared to unfiltered sunlight with a similar daily light integral. However, in the present experiments, green light also contributed to the inhibition of stem elongation as much as the blue light (Figure 4), emphasising the importance of green light in the growth regulation in chilli. The participation of green light in stem elongation was also observed in lettuce (Kim et al., 2004) and wheat (Monostori et al., 2018). However, the role of green light in the regulation is not known. It is possible that the effect of green light is mediated by a special green photoreceptor as supposed by Folta (2004), or it may be due to the absorption of cryptochromes, phototropins, or phytochromes in the green region (Sellaro et al., 2010). It is likely that the light-induced morphological responses are the consequence of regulated cooperation of many photoreceptors.

Flowering is also controlled by many photoreceptors; however, the regulation mechanism can vary according to long-, short-day or day neutral plants (Lin, 2000; Kim, 2020). The present experiments showed that flowering time in chilli was accelerated at high light intensity with the high amount of blue and green regions, and far-red light was also applied. Studies of flowering responses to light in the most studied *Arabidopsis* model plant showed that flowering was accelerated by far-red and blue light, whereas red light caused repression (Lin, 2000; Kim, 2020). These processes were mediated by the far-red sensitive phytochrome A and by the blue light-sensitive cryptrochrome 1 and 2 photoreceptors, respectively, and flowering repression appeared to be mediated by the red-sensitive phytochrome B (Lin, 2000; Kim, 2020). Although, to elucidate the role of photoreceptors was not the subject of this study, our results also confirmed the role of blue and far-red light in the acceleration of flowering. In addition, the present research also highlights the possible importance of the green region in the regulation of flowering.

In greenhouses and under natural light environments, chilli is often shaded for protection against stressful sunlight. Plants 25–80% shaded provided similar or higher yields than non-shaded plants, whereas their fruit produced more capsaicinoids and carotenoids and less phenolics than those of the uncovered plants (Ombódi et al., 2016; Jeeatid et al., 2017). Likewise, higher capsaicinoid content was detected in the fruit when moderate fluorescent light was used instead of applying high light intensity in a greenhouse during the summer season (Murakami et al., 2006). These results are in accordance with the present findings, where the capsaicin and carotenoid contents were lower, whereas the phenolic content was higher under high light intensity than under moderate light. In contrast, we could not confirm the previous observations (Gangadhar et al., 2012; Yap et al., 2021), indicating that blue light stimulated capsaicin accumulation, since a negative correlation was found between the amount of blue light and the accumulation of capsaicinoids and carotenoids. However, it must be mentioned that in the before-mentioned experiments, a high amount of capsaicinoids was detected when pure blue light was used either as sole (Gangadhar et al., 2012) or supplemental lighting (Yap et al., 2021). These light conditions completely differed from the mixtures used in the present experiments in terms of both light intensity and spectral composition. The present experiments showed that, among the light factors, the absolute amount of blue light was the main factor that affected the accumulation of capsaicinoids.

**FIGURE 5:**
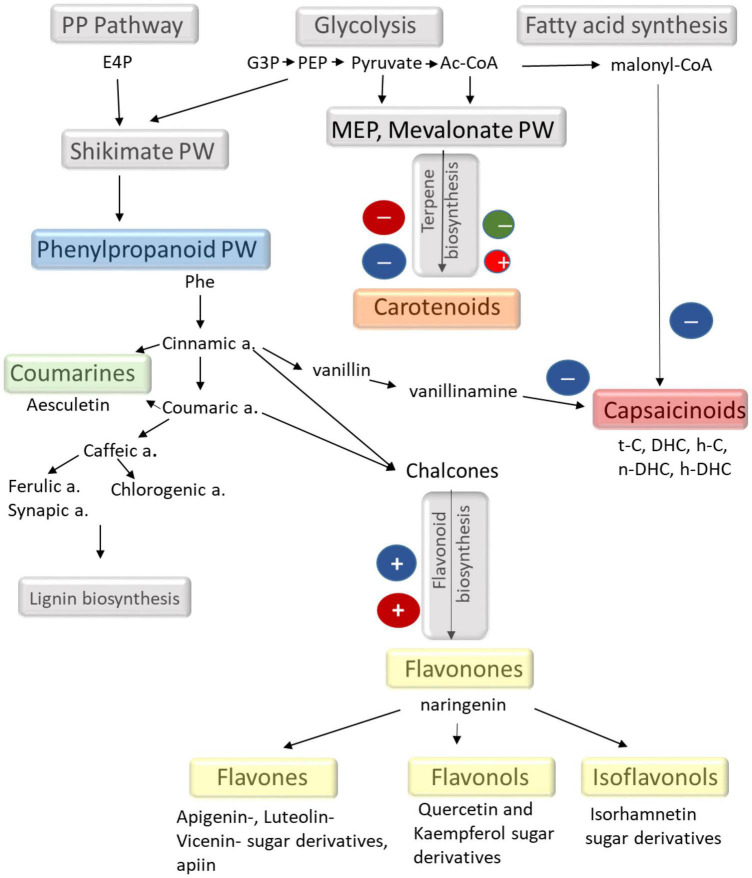
Schematic representation of different secondary metabolic pathways focusing on the metabolites found in the chilli fruit. The effects of light observed in the present experiments are also indicated. The scheme is based on the KEGG database (maps 00998, 00941, 00940, and 01060).

As summarised in Figure 5, capsaicinoids are synthetised from vanillin and phenylalanine *via* phenylpropanoid pathways together with the saturated (in DHC) or unsaturated (in C) alkyl chain (Antonio et al., 2018). Phenylpropanoid pathways are also linked with the synthesis of phenolics, including flavonoids, coumarins, and antocyanins (Figure 5). In spite of the fact that the metabolomic composition of chilli fruit grown under different environmental conditions (Antonio et al., 2018; Lemos et al., 2019; Kim et al., 2020) and the influence of light intensity and spectral composition on the accumulation of metabolites in several plants have already been investigated (Landi et al., 2020; Loi et al., 2021), the light regulation of secondary metabolism is still not understood in detail. The present results showed that the different light conditions induced approximately 40% change in capsaicinoids, 30% in carotenoids, and approximately 250% in phenolics. According to the results presented here, the blue light may induce a shift in the phenylpropanoid pathways. Namely, the synthesis of capsaicinoids was suppressed, whereas the synthesis of phenolics (mainly flavonoids) was stimulated by the amount of blue light. In addition, the inverse light-dependent behaviour of DHC and C suggests that light may also affect the saturation of the fatty acid chain during the synthesis of capsaicinoids. However, these metabolomic changes should be supported by further investigations.

Carotenoids, like β-carotene, α-carotene, different xanthophylls (zeaxanthin, neoxanthin, violaxanthin, lutein, etc.), are available in coloured vegetables and fruits and have strong radical quenching ability (Sarker et al., 2018b; Sarker and Oba, 2021). The red colour of chilli fruit mainly originates from capsanthin and capsorubin derived from violaxanthin and antheraxanthin. These highly apolar carotenoids are synthesised through the mevalonic acid pathways (Figure 5). According to the results presented here, it appeared that high light intensity did not force the accumulation of these metabolites (Figures 2, 4). In fact, it was the moderate light intensity and the higher proportion of red light that could stimulate their accumulation. However, the light-induced differences did not exceed 30%, which indicates that light is not a major factor in controlling carotenoid biosynthesis during fruit ripening. Abscisic acid and its derivatives and phaseic and dihydrophaseic acids are also part of the carotenoid pathways and are derived from same precursors as the red fruit colour. The low light-induced changes of these metabolites also suggested that light conditions were not detrimental for carotenoid biosynthesis in ripe fruits.

Phenolics and flavonoids, including hydroxybenzoicacids (Sarker and Oba, 2019a), hydroxycinnamic acids (Sarker and Oba, 2018d), flavanols (Sarker and Oba, 2020a), flavonols (Sarker et al., 2020), flavones (Sarker and Oba, 2020b), and flavanones (Sarker and Oba, 2020c) are strong antioxidants, having various health benefits (Sarker and Oba, 2018e). These compounds are also associated with some physiological activities in plants, such as reducing reactive oxygen species and oxidative damage (ROS) (Sarker and Oba, 2018b), alleviating osmotic stress (Sarker and Oba, 2018c), decreasing photosynthetic activities (Sarker and Oba, 2018e), improving nutrient imbalance (Sarker et al., 2018a) in plant cells, protecting plants from drastic reduction in growth and productivity (Sarker and Oba, 2019c), and ultimately enhancing the concentration of antioxidants (Sarker and Oba, 2018a) for human diet. The strong antioxidant properties of phenolics are also confirmed in the present experiments, as the radical scavenging activity of the fruit (calculated as DPPH%) mainly correlated (*r* = 0.716; *p* = 0.0136) with the total amount of phenolics. Furthermore, both of these showed a positive correlation with the amount of blue light, which also supports that phenolic compounds contribute significantly to antioxidant capacity. Likewise, positive and significant strong associations were observed between total phenolics and antioxidant capacity (DPPH) in several *Amaranthus* species (Sarker et al., 2018a,Sarker and Oba, 2018c; Sarker and Oba, 2019b,c) that corroborate the present findings.

As indicated by Marín et al. (2004), most flavonoids are present in the form of conjugated O-glycosides and C-glycosides in chilli. Many glycosylated derivatives of luteolin, quercetin, kaempferol, and apigenin were also identified in the present study (Figure 3 and Supplementary Table 1), similarly as found by Morales-Soto et al. (2013) and Kelebek et al. (2020). To our knowledge, this is the first report describing the light dependence of separated flavonoid compounds in chilli. Comparison of the light dependence of different phenolic compounds showed that most flavonoids, including luteolin, apigenin, naringenin, quercetin, kaempferol, and isorhamnetin derivatives, showed positive correlation with the amount of blue light, similarly as found for total phenolics and antioxidant-scavenging activity in fruits. This suggested that flavonoids belonging to flavone, flavonone, and flavonol groups were responsible for the accumulation of phenolics and also contributed to the antioxidant capacity of the fruit. Other phenolics belonging to the hydroxycinnamic acid family, such as chlorogenic, ferulic, and sinapic acids, and their derivatives were also found in the fruit pericarp; however, these metabolites showed less light dependence than flavonoids, except for sinapic acid and its derivatives.

Although the molecular mechanisms of flavonoid biosynthesis have been extensively studied (Tohge et al., 2014; Tang et al., 2019; Thoma et al., 2020; Al Murad et al., 2021), their light regulation has not yet been elucidated (Landi et al., 2020; Loi et al., 2021). The light regulatory units of the genes for the key enzymes, such as phenylalanine ammonia lyase (PAL), 4-coumaroyl CoA-ligase (4CL), and chalcone synthase (CHS) involved in flavonoid biosynthetic pathways, have already been identified in *Arabidopsis* and in some fruit species like grapes and apple (Hartmann et al., 2005; Zoratti et al., 2014; Liu et al., 2018). Based on these investigations, flavonoid biosynthesis may be mediated by the UVB and blue photoreceptors, such as phototropins and cryptochromes (Landi et al., 2020; Loi et al., 2021). Although the photoreceptors were not identified here, the present results also supported that phenolics, especially flavonoids, were strongly regulated by blue light in this chilli. In addition, both phenolic content and antioxidant capacity of the chilli fruit can be improved by the addition of a high amount of blue light. The light-induced metabolomic changes in chilli are summarised in a schematic figure (Figure 5). From these results, we can conclude that the application of LED lighting can be an alternative way to improve fruit quality in terms of bioactive compound.

## Conclusion

In the present study, the light factors affecting the growth and development of chilli and the production of secondary metabolites were investigated. The results demonstrated that light fluence and spectral composition together affected the growth, flowering time, and fruit production of chilli, in which the absolute amount of different spectral regions paid an important role; however, the proportion of green light and the R/B ratio also had an influence. Among the light-induced changes, the increase of light fluence and the proportion of blue light stimulated flowering and yield in chilli, whereas low amounts of blue light enhanced green mass accumulation. Secondary metabolite production in the fruit was also affected by spectral composition and light fluence. Blue light (its absolute amount) stimulated the accumulation of phenolic compounds, but it inversely affected the production of capsaicinoids and carotenoids. These results proved that the accumulation of secondary metabolites can be modified by appropriate modification of light fluence and spectral composition, but different spectral combinations are required to induce the accumulation of different types of secondary metabolites. A single spectral combination is not sufficient to ensure the optimal growth of chilli and the accumulation of all metabolites; only an adjustable light environment can ensure such conditions in indoor cultivation systems.

## Data Availability Statement

The original contributions presented in the study are included in the article/Supplementary Material, further inquiries can be directed to the corresponding author/s.

## Author Contributions

ED and GG conceived the research plan. ED and MA performed most of the experiments. KH and MD carried out the metabolomic analysis on capsaicinoids and phenolics. TM analysed the data. ED wrote the article. All authors contributed to the article and approved the submitted version.

## Conflict of Interest

 The authors declare that the research was conducted in the absence of any commercial or financial relationships that could be construed as a potential conflict of interest.

## Publisher’s Note

All claims expressed in this article are solely those of the authors and do not necessarily represent those of their affiliated organizations, or those of the publisher, the editors and the reviewers. Any product that may be evaluated in this article, or claim that may be made by its manufacturer, is not guaranteed or endorsed by the publisher.
