# Supplementary material for: Modulated Light Dependence of Growth, Flowering, and the Accumulation of Secondary Metabolites in Chilli
Source: Front Plant Sci. 2022 Mar 22;13:801656. doi: 10.3389/fpls.2022.801656 (PMC8981241; doi:10.3389/fpls.2022.801656)
Supplement: Supplementary file 1 [file Table_1.pdf]

Supplementary information for the article entitled

Modulated light dependence of growth, flowering and the accumulation of secondary metabolites in chilli

published in Frontiers in Plant Science
(section Crop and Product Physiology; manuscript No. 801656;
doi: 10.3389/fpls.2022.801656)

by

Eva Darko[1]*, Kamirán A. Hamow[1], Tihana Marcek[2], Mihály Dernovics[1], Mohamed Ahres[1] and Gábor Galiba[1,3]

[1] Centre for Agricultural Research, Agricultural Institute, Martonvásár, Hungary

[2] Faculty of Food Technology, Josip Juraj Strossmayer University of Osijek, Osijek, Croatia

[3] Hungarian University of Agriculture and Life Sciences, Keszthely, Hungary

*corresponding author

Supplemenraty Table 1: The accumulation of phenolic compounds in the fruit. 5 samples were analysed for each light regimen where each sample consisted of 10 pieces of fruit. Data are mean± standard deviation of 5 replicates per light treatments. A: red and blue LEDs with 95:5 ratio at moderate light (ML); B: red and blue LEDs with 80:20 ration at ML; C: wide spectrum at ML; D: wide spectrum with FR applications at ML, E: wide spectrum with FR at high light (HL); F: wide spectrum supplemented with FR and blue LEDs at HL. HI: harvest index calculated as the ratio of fruit yield and total above ground biomass production.

ng/g FW
µg / g FW

	A	B	C	D	E	F
			Phenolic acids			
Chlorogenic acid	285±20.3	300±28.4	221±26.4	358±34.6	369±37	611±41.5
Sinapoyl hexoside	2.89±0.09	3.56±0.23	3.57±0.07	3.29±0.10	4.77±0.42	5.11±0.50
Feruloyl hexoside	33.7±1.1	31.84±1.8	28.6±1.69	37.3±1.85	38.2±1.84	33.6±1.40
			Coumarins			
Aesculetin	84±3.3	67±4.8	85±2.1	74±2.4	77±4.9	92±5.6
			Phenylpropanoids			
Sinapic acid	2.2±0.33	3.1±0.30	2.6±0.17	3.4±0.29	3.4±0.27	3.3±0.22
Ferulic acid	281±23.9	447±42.0	269±20.6	284±37.6	210±23.1	310±29.2
			Flavones			
Luteolin-8-C-glucoside	48.6±1.33	126.9±10.1	73.4±2.22	119.45±5.6	114.2±7.2	162.2±10.2
Apiin	1.4±0.03	2.1±0.21	1.6±0.03	1.7±0.11	3.0±0.48	4.6±0.325
Apigenin C-pentosyl-C-hexoside isomer 1	1.3±0.16	1.9±0.17	1.5±0.06	2.1±0.11	2.3±0.26	2.9±0.14
Apigenin C-pentosyl-C-hexoside isomer 2	0.72±0.08	0.80±0.07	0.76±0.07	1.00±0.09	1.22±0.10	0.9±0.10
Luteolin-8-C-glucoside	2.4±0.13	2.5±0.21	2.6±0.04	3.5±0.18	3.8±0.22	3.1±0.19
Vicenin-2	2.0±0.16	2.9±0.16	2.2±0.08	3.0±0.09	3.4±0.21	4.2±0.17
Apigenin C-pentosyl-C-hexoside isomer 3	1.77±0.14	2.84±0.20	1.9±0.10	3.1±0.09	2.9±0.19	4.6±0.17
Luteolin O-(apiosyl)hexoside	0.08±0.01	0.81±0.09	0.14±0.08	0.13±0.05	0.82±0.17	1.59±0.14
Luteolin O-(apiosylmalonyl)glucoside	3.1±0.13	9.9±0.91	4.5±0.9	4.1±0.23	11.1±1.1	13.2±1.3
6''-malonylapiin	3.9±0.20	6.5±0.6	4.5±0.34	4.5±0.26	12.0±1.50	8.1±0.62
Apigenin derivative	0.6±0.02	1.8±0.17	0.9±0.06	0.7±0.42	1.7±0.23	3.76±0.45
			Flavonones			
Naringenin	12.7±2.2	139.5±13.5	11.9±2.1	70.9±9.3	229±15.6	438±65.2
			Flavonols			
Quercetin-3,4'-diglucoside	1.1±0.21	1.2±0.11	1.9±0.25	1.6±0.9	4.4±0.9	1.1±0.2
Quercetin-3-O-glucoside	5.3±0.32	119±9.6	23.1±3.9	7.1±0.5	93±12.5	127±9.4
Quercetin-3-O-rhamnoside	32.5±1.2	510±50	99±6.7	35±2.7	329±33	401±48
Quercetin-Glc-Rha	31±1.7	86±4.1	130±6.7	89±5.5	190±5.5	84±3.2
Kaempferol-3-O-glucoside	51±2.4	159±14	80±5.4	46±1.6	115±15.3	202±9.8
Isorhamnetin-3-O-glucoside	37±3.7	126±11.4	71±3.4	54±2.0	178±27	160±14.5
Isorhamnetin-3-rutinoside	136±15	502±37	175±11	308±12	353±45	597±30
			Carotenoid derivatives			
Dihydro-phaseic acid	12.5±1.0	16.4±1.1	16.9±1.3	17.3±1.2	19.3±0.8	19.7±0.8
Abscisic acid	64±2.1	67±3.3	70±1.5	60±1.8	79±1.2	66±3.7
Phaseic acid	111±2.7	130±6.5	119±2.3	132±6.1	139±2.6	135±5.2
			Capsianosides			
Capsianoside III	1.4±0.13	3.7±0.2	2.1±0.16	2.8±0.17	2.3±0.17	2.3±0.17
Capsianoside IV diglucoside 1	1.7±0.10	1.9±0.15	1.6±0.13	2.2±0.05	1.9±0.20	2.3±0.11
Capsianoside IV diglucoside 2	1.4±0.13	1.8±0.15	1.2±0.07	1.8±0.05	1.80.12	2.1±0.25
Capsianoside derivative	0.97±0.05	0.69±0.06	0.90±0.03	1.00±0.07	1.00±0.08	1.2±0.08
Capsianoside V	0.34±0.06	0.52±0.04	0.42±0.02	0.41±0.04	0.58±0.07	0.05±0.07
			Non-identified compouds			
NI1	7.5±0.24	6.4±0.34	7.8±0.25	5.9±0.23	8.1±0.23	5.6±0.40
NI2	7.0±0.20	5.8±0.31	6.5±0.10	6.9±0.12	7.9±0.33	7.2±0.31
NI3	1077±51	747±63	791±43	993±25	1313±54	855±28
NI4	1.0±0.03	1.1±0.14	1.1±0.13	1.2±0.15	1.1±0.10	1.6±0.12
NI5	2.8±0.12	2.5±0.17	1.9±0.06	1.1±0.13	3.9±0.16	3.18±0.15
NI6	6.0±0.85	8.2±0.70	6.2±0.11	8.3±0.34	9.3±0.86	8.7±0.52
NI7	5.2±0.21	5.1±0.35	4.9±0.16	6.5±0.20	6.5±0.15	6.7±0.27
NI8	3.5±0.22	3.1±0.15	3.2±0.11	3.3±0.12	3.6±0.27	4.3±0.25
NI9	494±53	848±71	581±61	769±59	944±46	1023±59
NI10	9.0±0.24	11.7±0.68	9.3±0.20	14.0±1.20	13.6±1.2	17.3±0.99
NI11	3.7±0.14	3.8±0.22	3.54±0.07	5.1±0.20	5.4±0.27	4.5±0.40
NI12	4.7±0.15	4.9±0.29	4.4±0.16	5.7±0.38	5.8±0.58	6.8±0.53
NI13	1.9±0.07	1.8±0.08	1.6±0.19	2.0±0.09	2.8±0.12	2.0±0.09
NI14	2.9±0.16	2.9±0.20	2.4±0.08	3.3±0.13	3.5±0.49	3.2±0.32
NI15	5.5±0.14	6.3±0.30	6.1±0.10	6.8±0.15	9.5±0.61	8.1±0.26
NI16	0.90±0.10	0.95±0.03	1.10±0.13	1.21±0.07	1.51±0.10	1.29±0.11
NI17	4.1±0.09	5.2±0.33	3.2±0.10	3.6±0.08	4.4±0.13	5.1±0.21
NI18	2.1±0.13	2.8±0.14	1.7±0.13	1.4±0.05	2.5±0.14	2.6±0.10
NI19	0.60±0.09	0.93±0.06	0.68±0.06	0.56±0.09	1.04±0.05	1.24±0.03
NI20	1.12±0.09	1.25±0.09	0.86±0.07	0.74±0.06	1.08±0.07	1.34±0.10
