# Supplementary material for: Modulated Light Dependence of Growth, Flowering, and the Accumulation of Secondary Metabolites in Chilli
Source: Front Plant Sci. 2022 Mar 22;13:801656. doi: 10.3389/fpls.2022.801656 (PMC8981241; doi:10.3389/fpls.2022.801656)

HR-MS# supplementary information for the article entitled

Modulated light dependence of growth, flowering and the accumulation of secondary metabolites in chilli

published in Frontiers in Plant Science
(section Crop and Product Physiology; manuscript No. 801656;
doi: 10.3389/fpls.2022.801656)

by

Eva Darko[1]*, Kamirán A. Hamow[1], Tihana Marcek[2], Mihály Dernovics[1], Mohamed Ahres[1] and Gábor Galiba[1,3]

[1] Centre for Agricultural Research, Agricultural Institute, Martonvásár, Hungary

[2] Faculty of Food Technology, Josip Juraj Strossmayer University of Osijek, Osijek, Croatia

[3] Hungarian University of Agriculture and Life Sciences, Keszthely, Hungary

*corresponding author

for accurate mass MS data and adduct information, see the Supplementary Information on instrumental parameters

MS/MS spectrum for the compound feruloyl hexoside
ESI-MS mode: negative, Unispray ion source

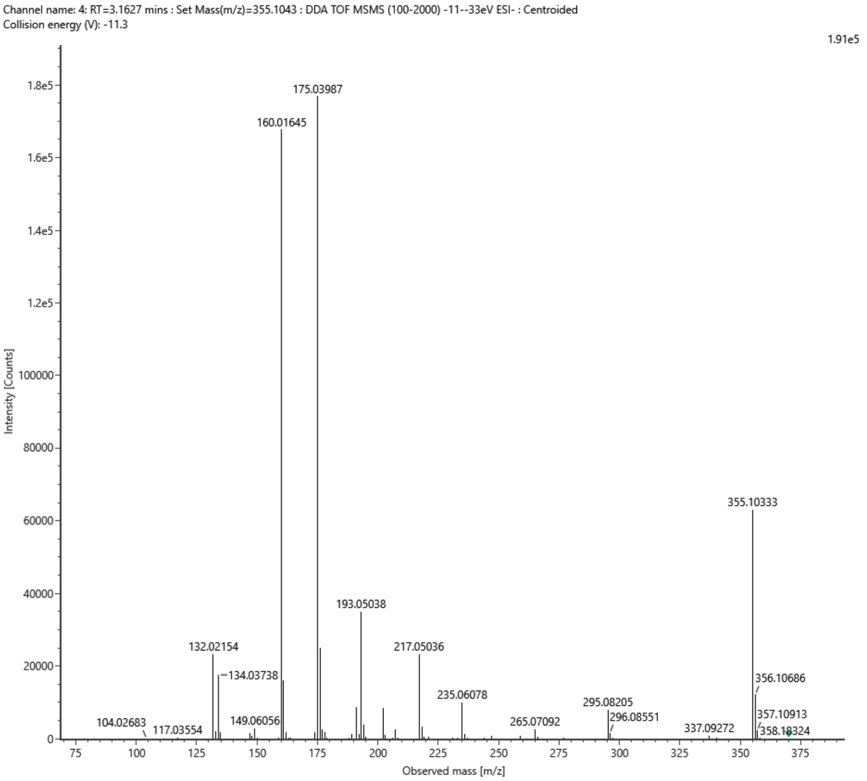

MS/MS spectrum for the compound sinapoyl hexoside
ESI-MS mode: negative, Unispray ion source

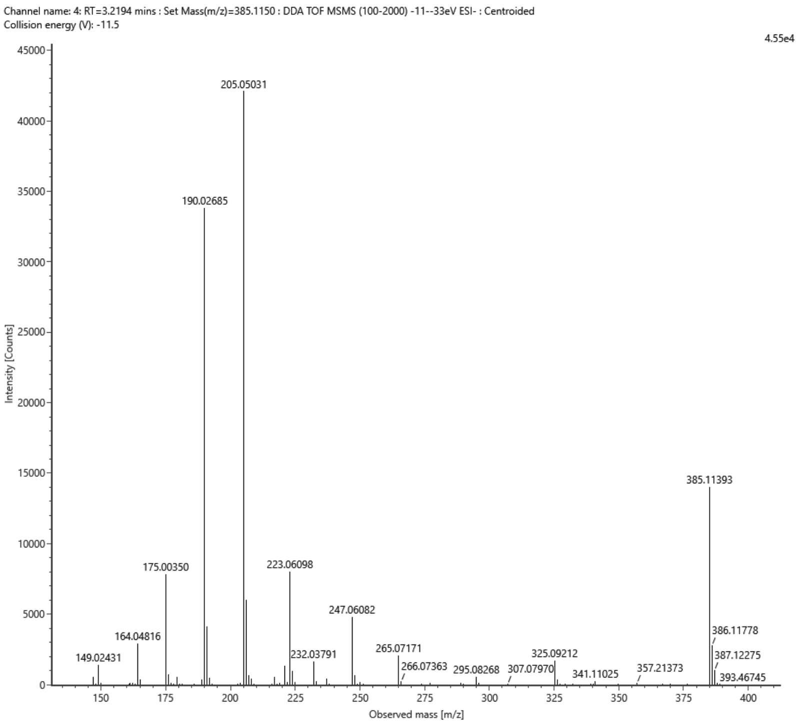

MS/MS spectrum for the compound Vicenin-2
ESI-MS mode: negative, Unispray ion source

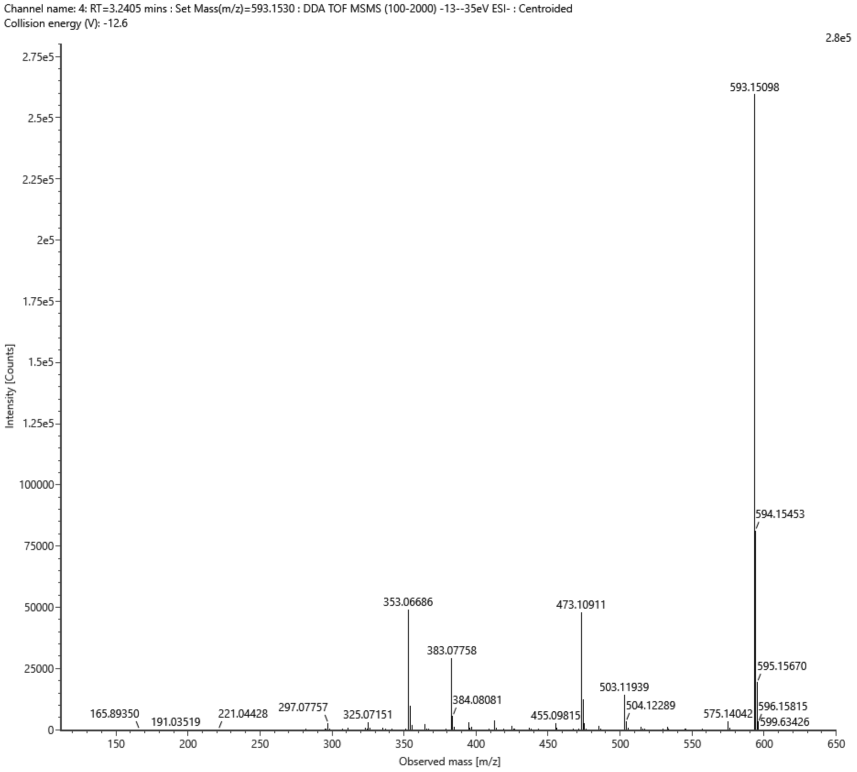

MS/MS spectrum for the compound apigenin C-pentosyl-C-hexoside
ESI-MS mode: negative, Unispray ion source
(R_t=3.50 min)

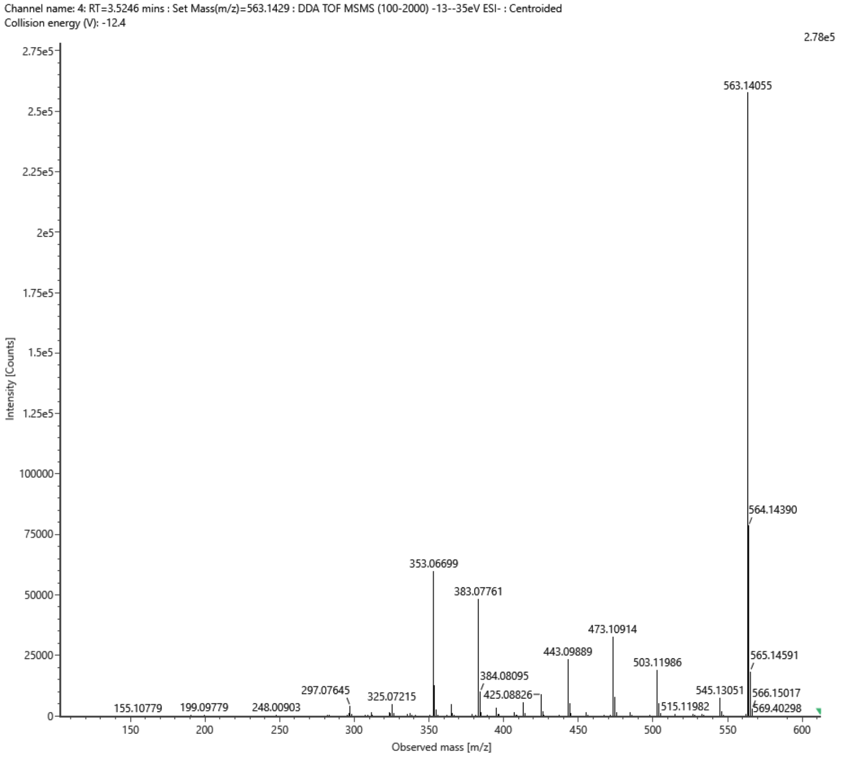

MS/MS spectrum for the compound apigenin C-pentosyl-C-hexoside
ESI-MS mode: negative, Unispray ion source
(R_t=3.63 min)

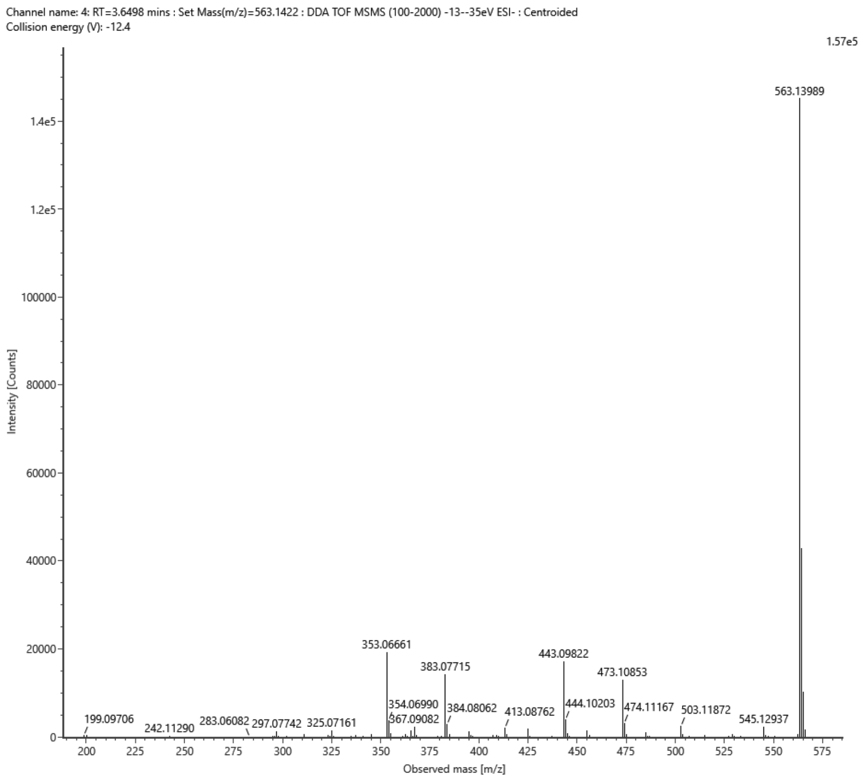

MS/MS spectrum for the compound luteolin-8-C-hexoside
ESI-MS mode: negative, Unispray ion source

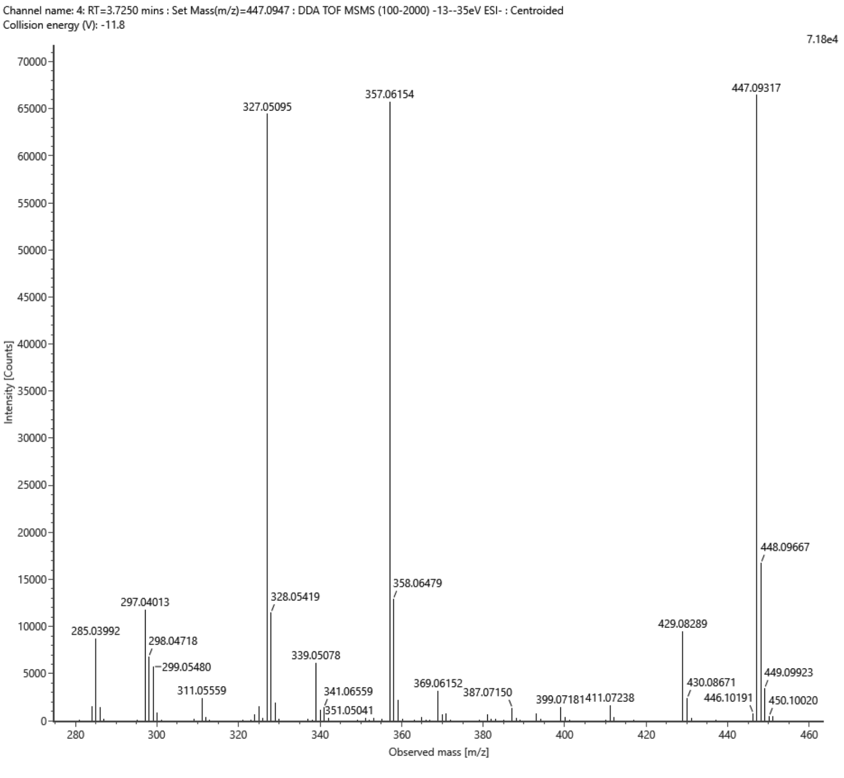

MS/MS spectrum for the compound apigenin C-pentosyl-C-hexoside
ESI-MS mode: negative, Unispray ion source
(R_t=3.82 min)

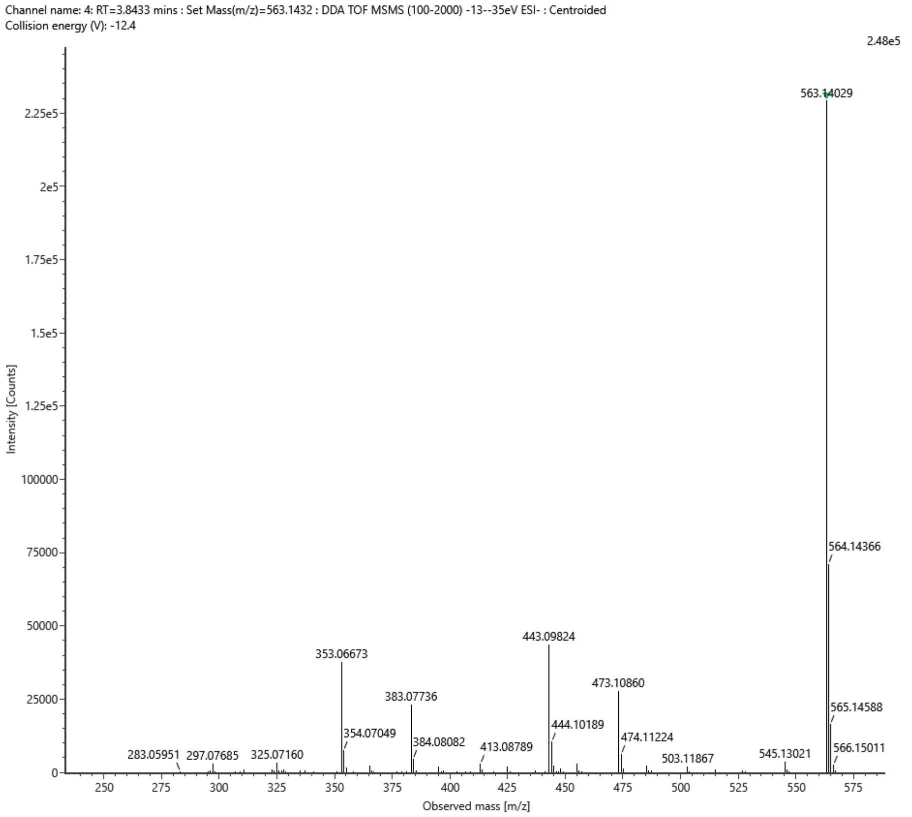

MS/MS spectrum for the compound luteolin O-(apiosyl)hexoside
ESI-MS mode: negative, Unispray ion source

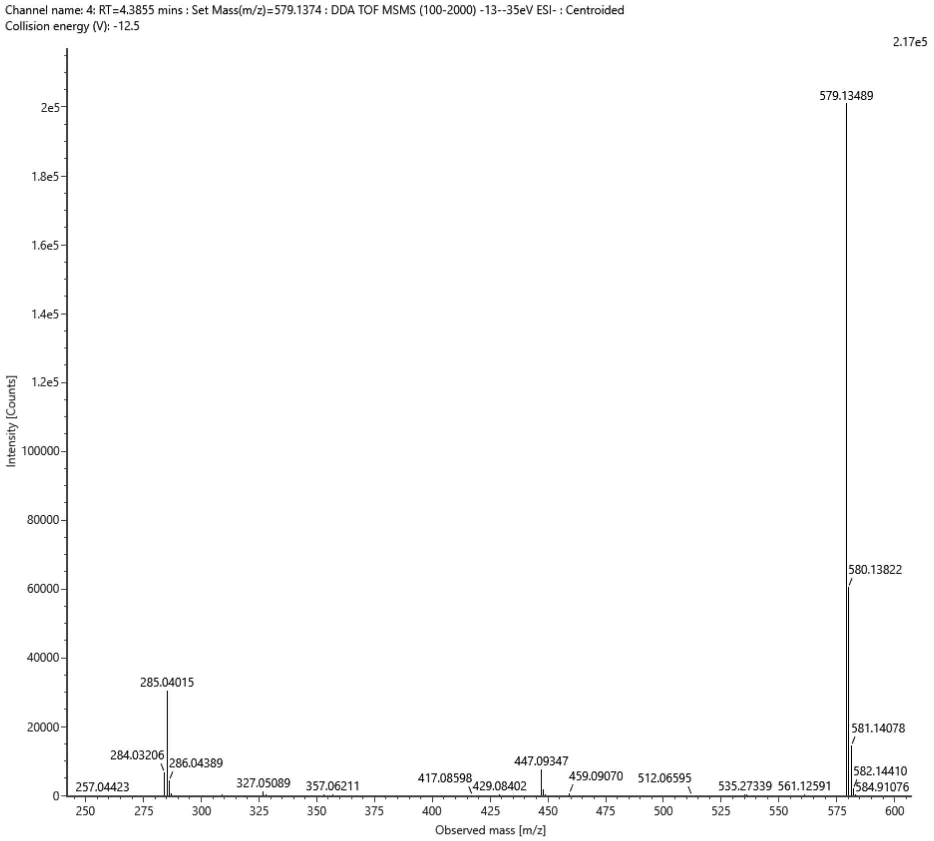

MS/MS spectrum for the compound luteolin O-(apiosylmalonyl)glucoside
ESI-MS mode: negative, Unispray ion source
(note the low abundance of the parent ion at m/z 665)

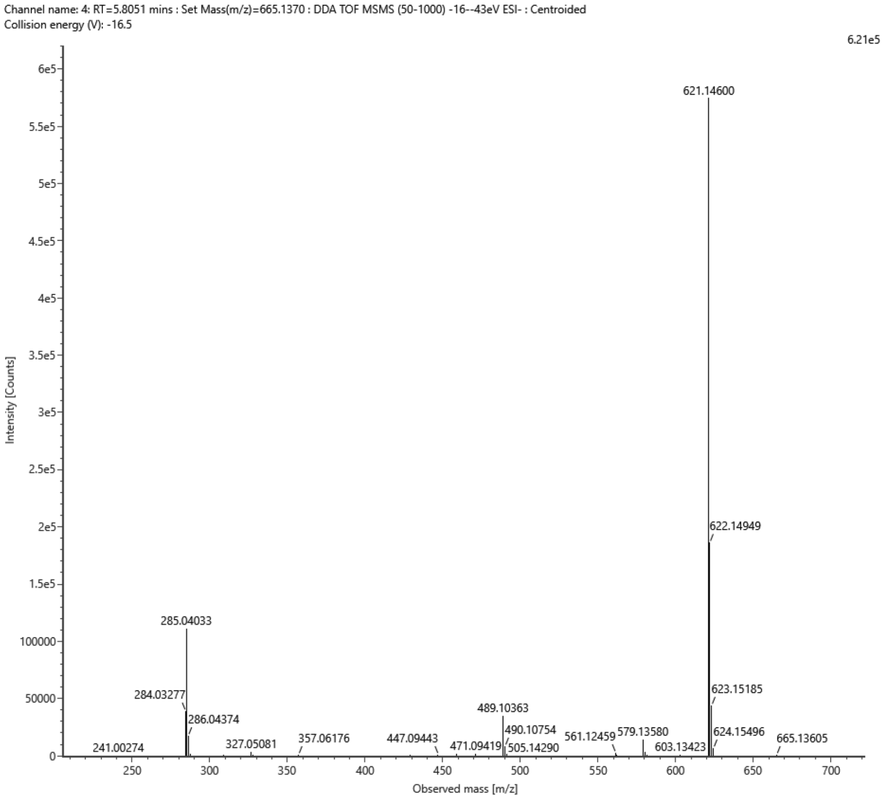

MS/MS spectrum for the compound 6''-acetylapiin
ESI-MS mode: negative, Unispray ion source
(note the isobaric interference during MS/MS at m/z 606)

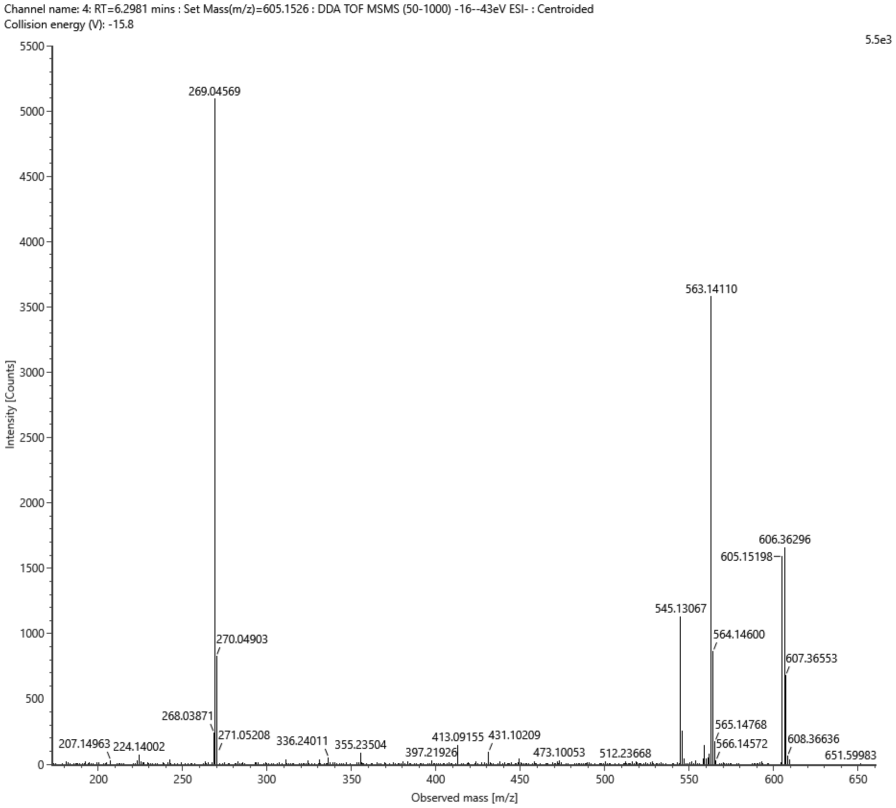

MS/MS spectrum for the compound 6''-malonylapiin

ESI-MS mode: negative, Unispray ion source

(note the low abundance of the parent ion at m/z 649)

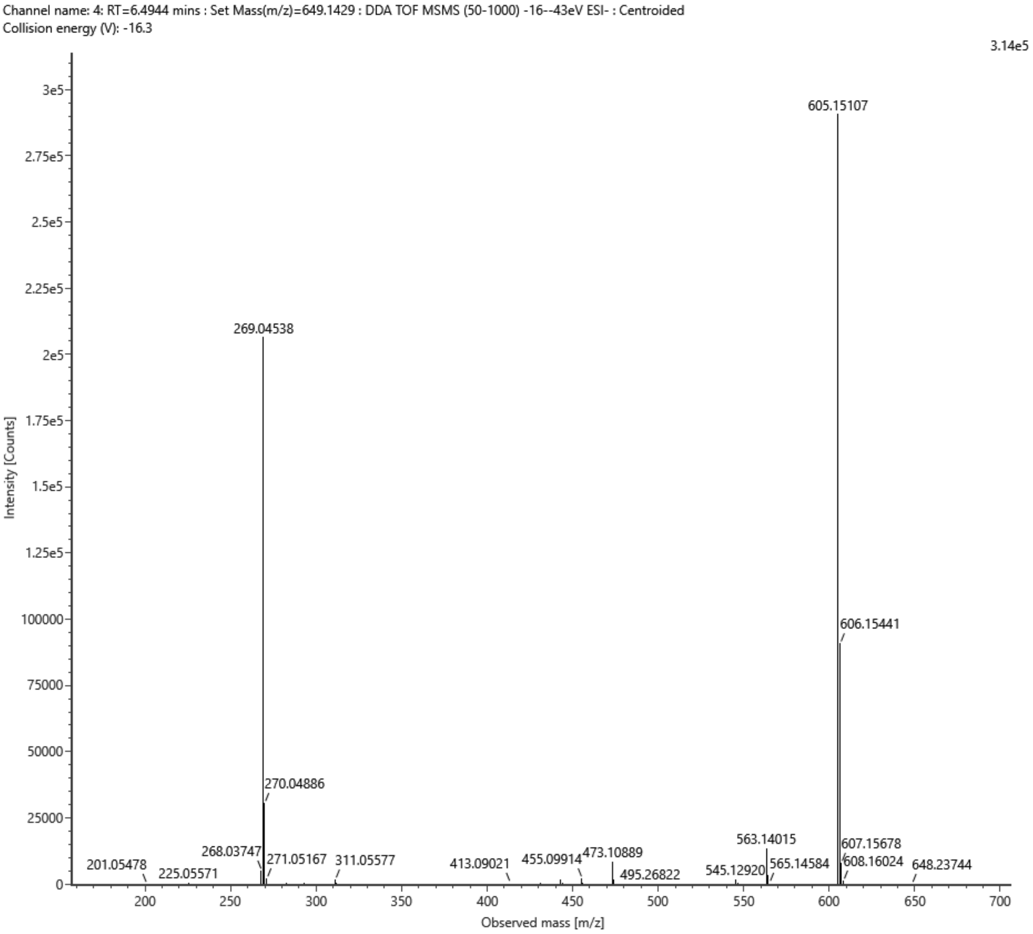

MS/MS spectrum for the compound feruloyl hexoside
ESI-MS mode: positive, Unispray ion source

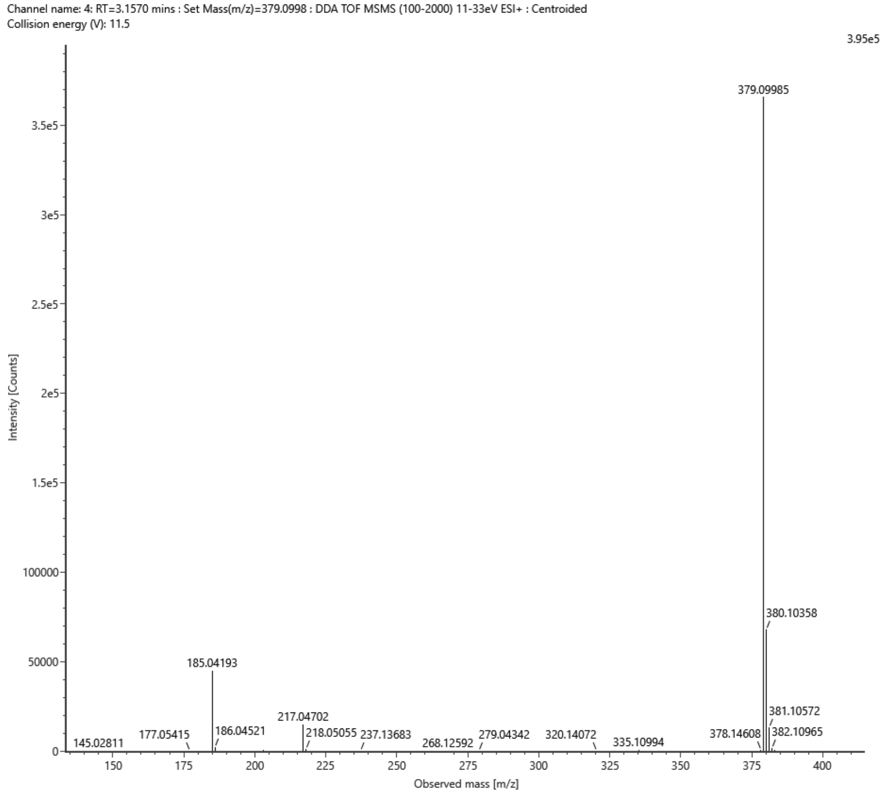

MS/MS spectrum for the compound sinapoyl hexoside
ESI-MS mode: positive, Unispray ion source

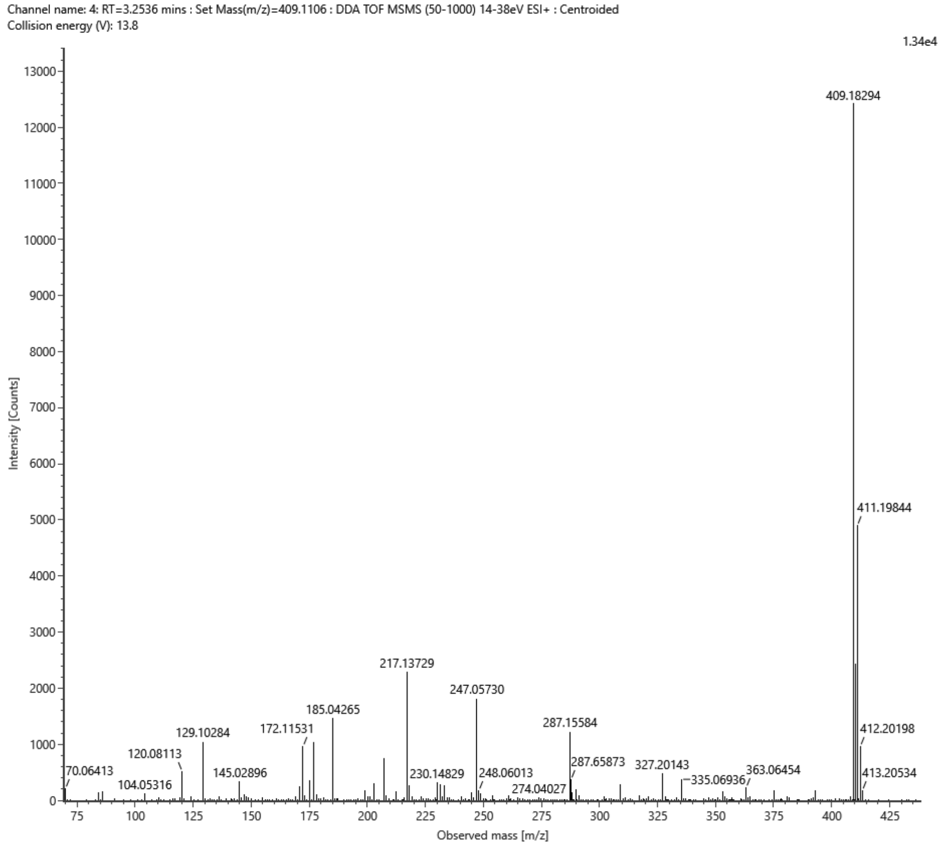

MS/MS spectrum for the compound Vicenin-2
ESI-MS mode: positive, Unispray ion source

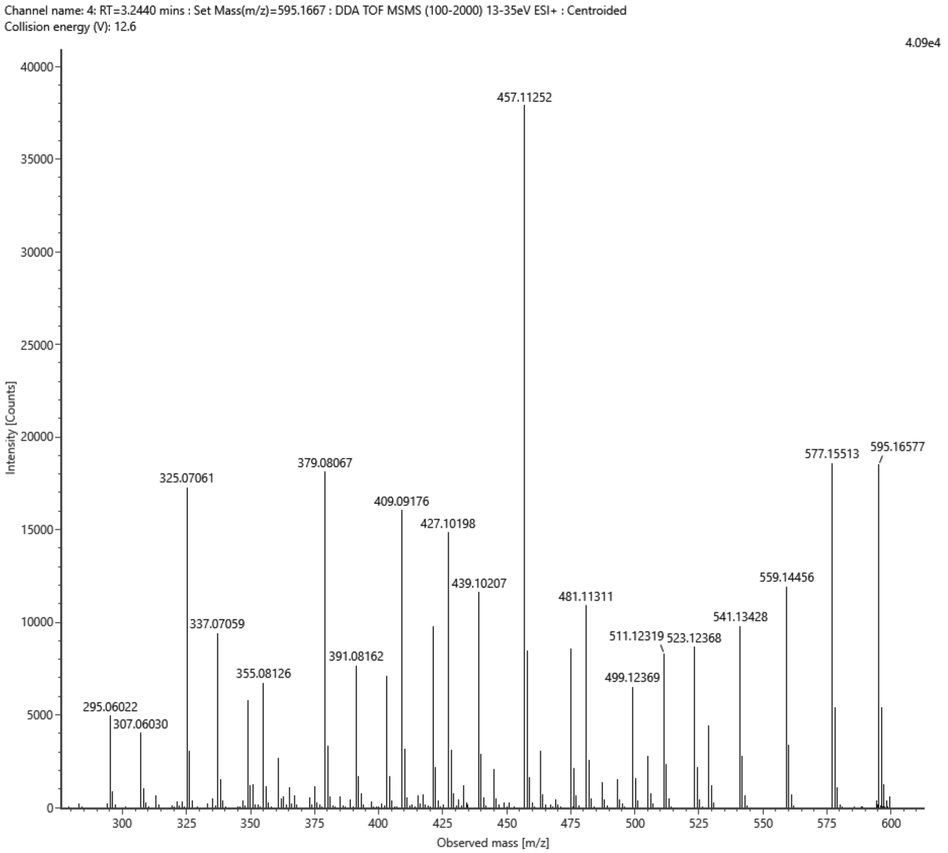

MS/MS spectrum for the compound apigenin C-pentosyl-C-hexoside
ESI-MS mode: positive, Unispray ion source
(R_t=3.50 min)

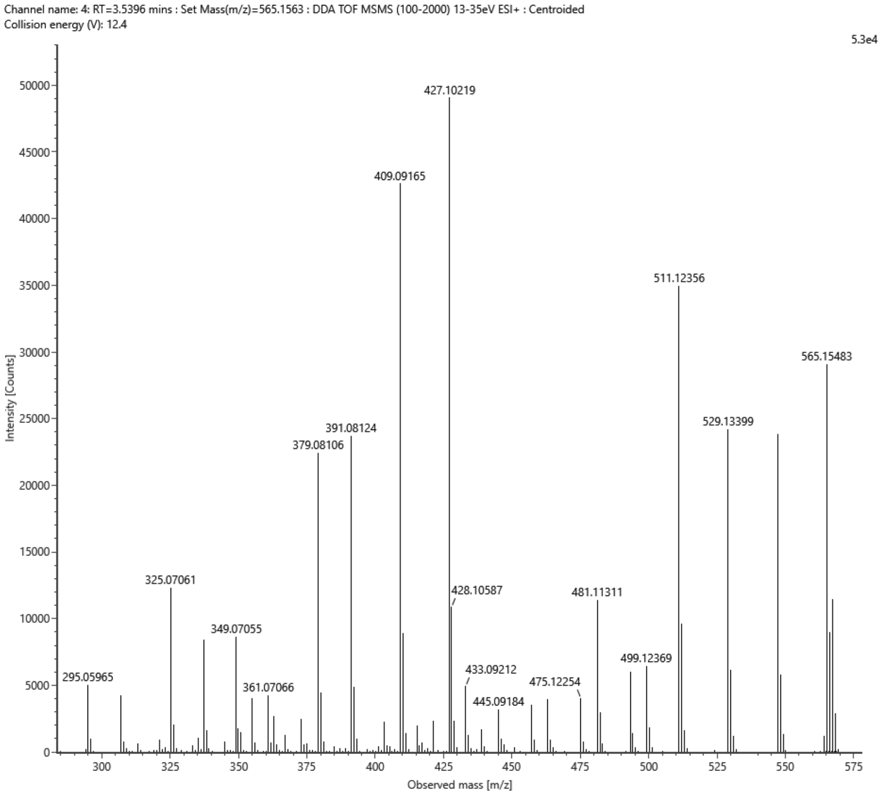

MS/MS spectrum for the compound apigenin C-pentosyl-C-hexoside
ESI-MS mode: positive, Unispray ion source
(R_t=3.63 min)

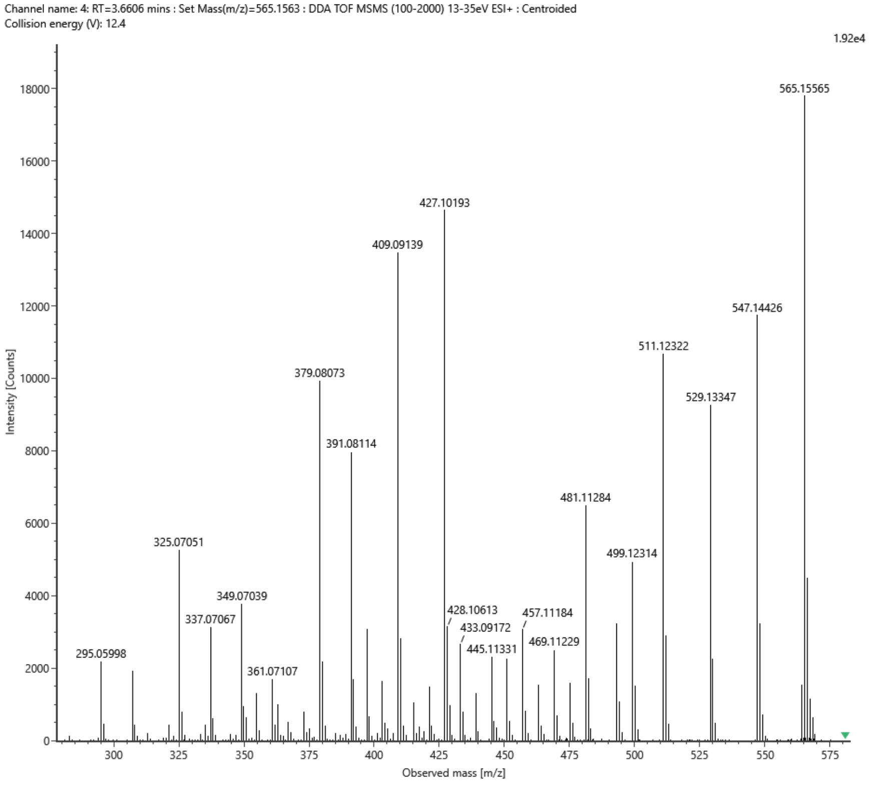

MS/MS spectrum for the compound luteolin-8-C-hexoside
ESI-MS mode: positive, Unispray ion source

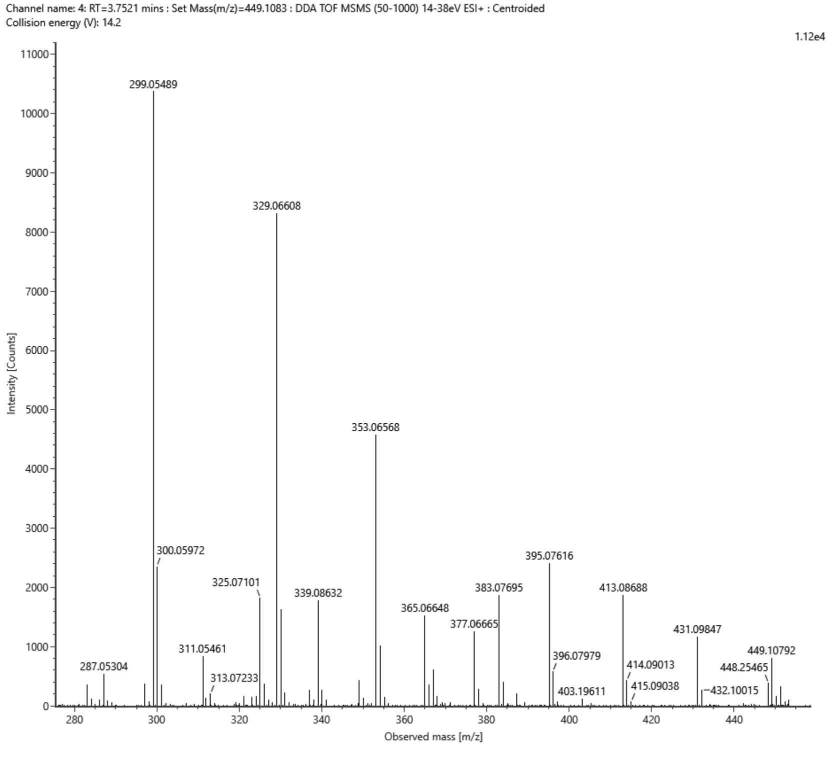

MS/MS spectrum for the compound apigenin C-pentosyl-C-hexoside
ESI-MS mode: positive, Unispray ion source
(R_t=3.82 min)

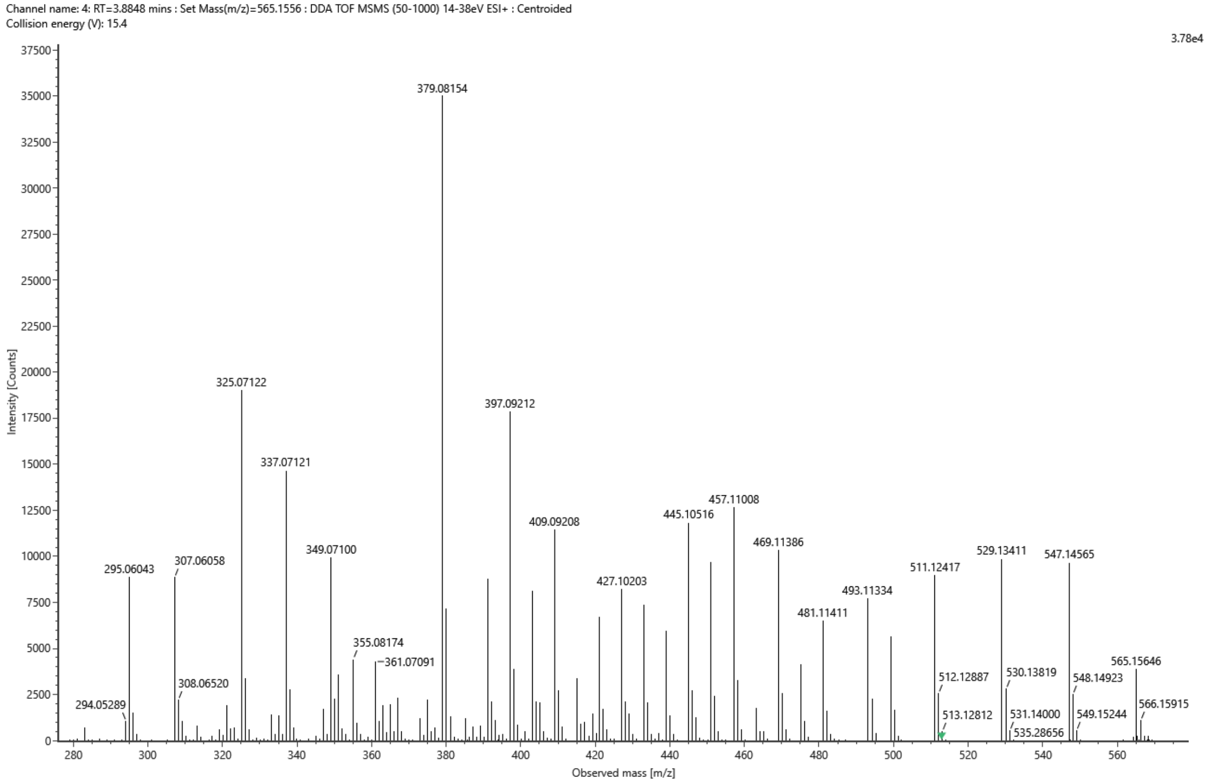

MS/MS spectrum for the compound luteolin O-(apiosyl)hexoside
ESI-MS mode: positive, Unispray ion source

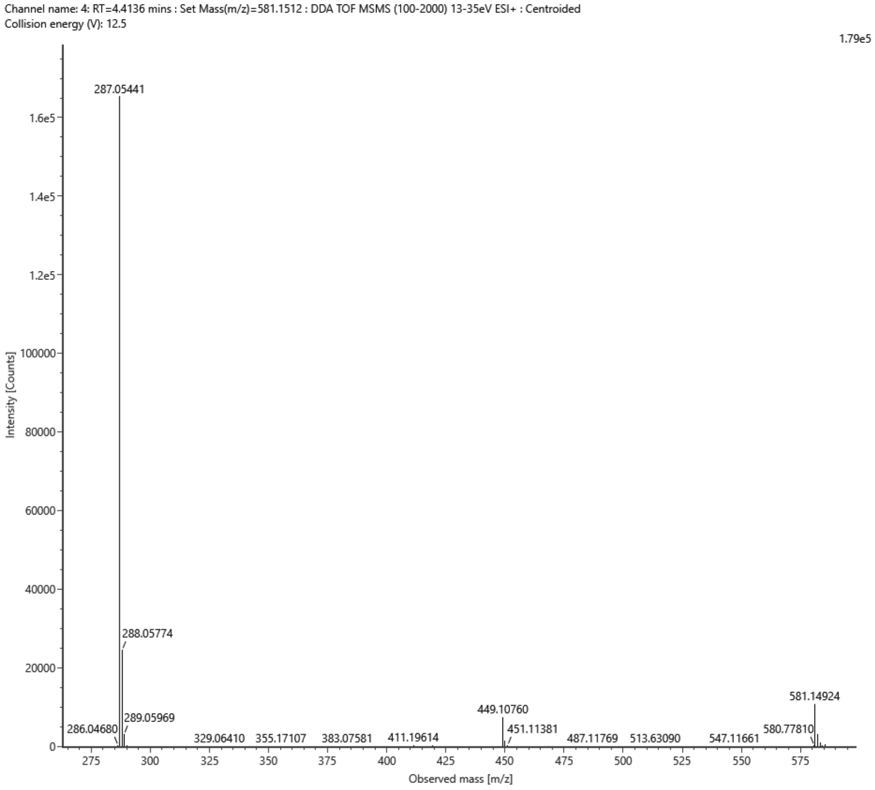

MS/MS spectrum for the compound luteolin O-(apiosylmalonyl)glucoside
ESI-MS mode: positive, Unispray ion source

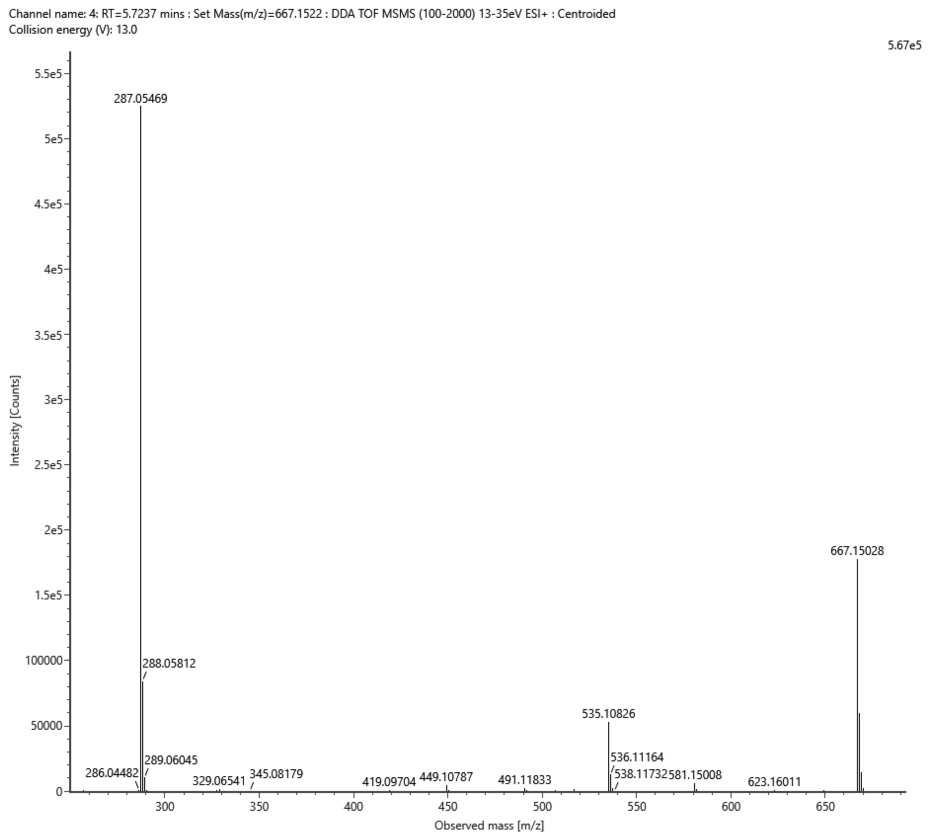

MS/MS spectrum for the compound 6''-malonylapiin
ESI-MS mode: positive, Unispray ion source

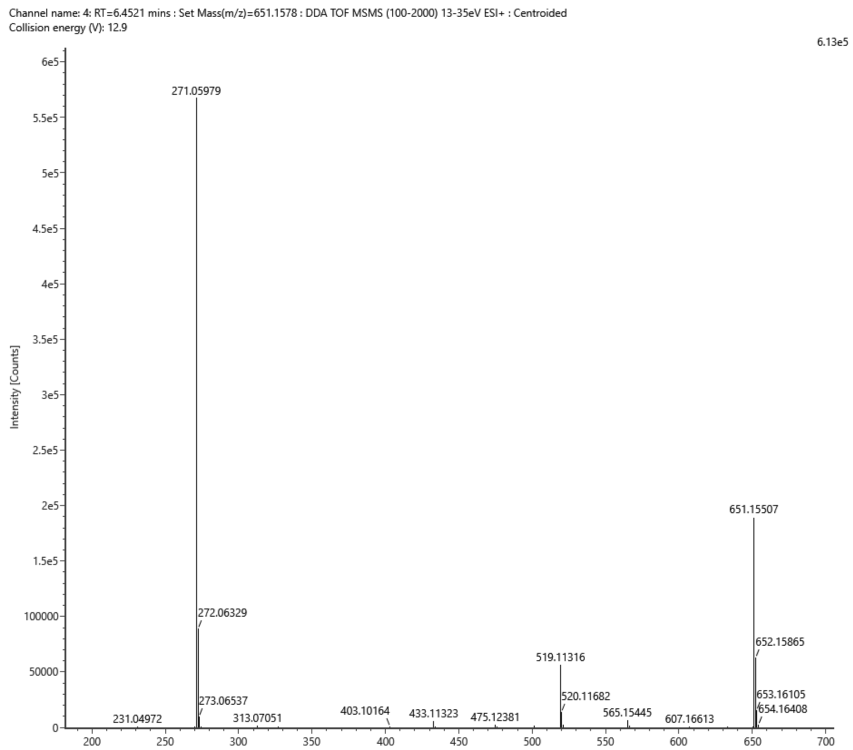

MS spectrum for the compound Capsianoside-III (Na+ adduct)
ESI-MS mode: positive, Unispray ion source

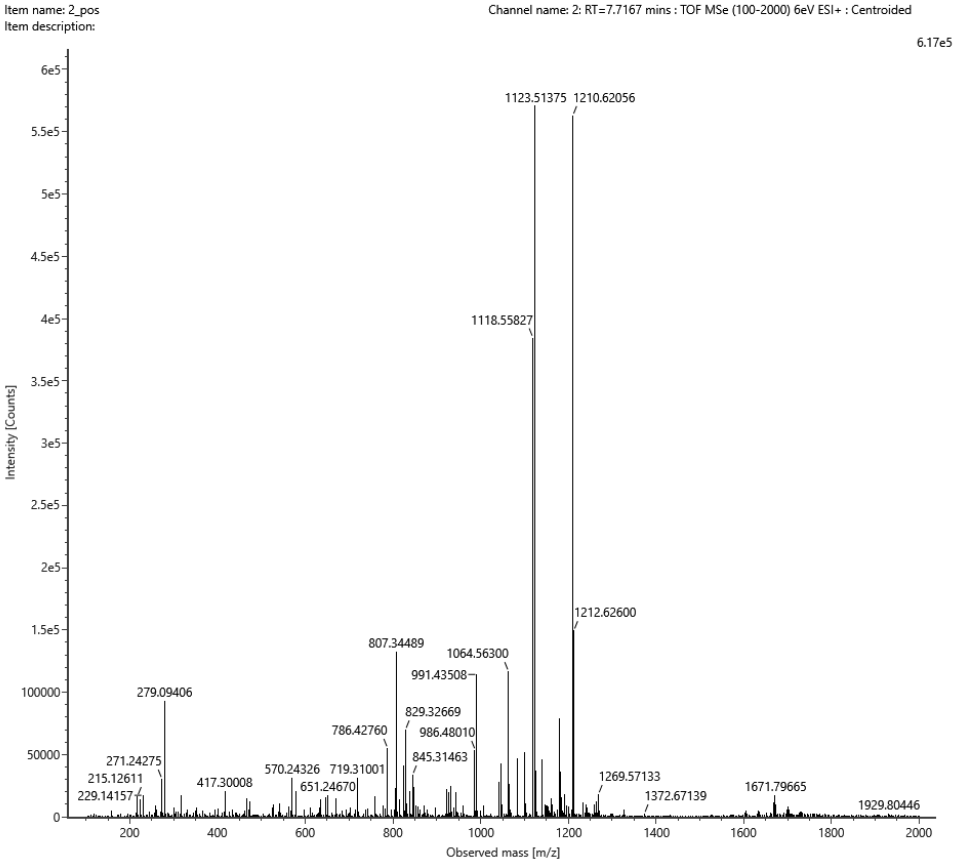

MS spectrum for the compound Capsianoside-III
ESI-MS mode: negative, Unispray ion source

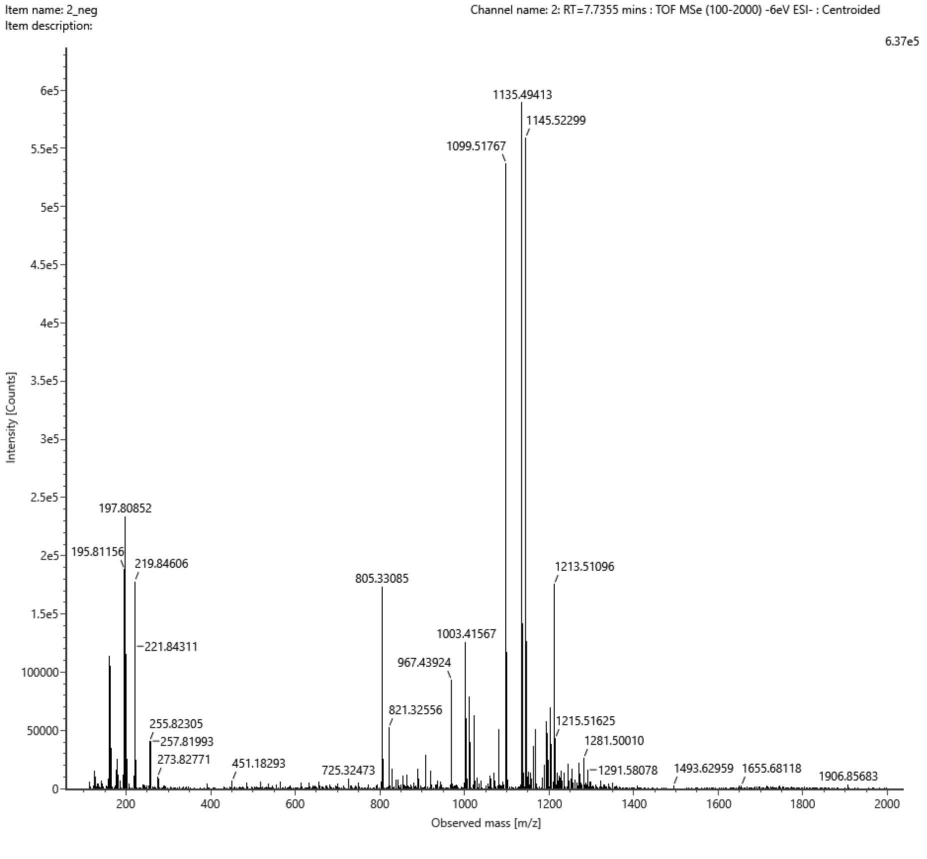

MS/MS spectrum for the compound Capsianoside-III
ESI-MS mode: positive

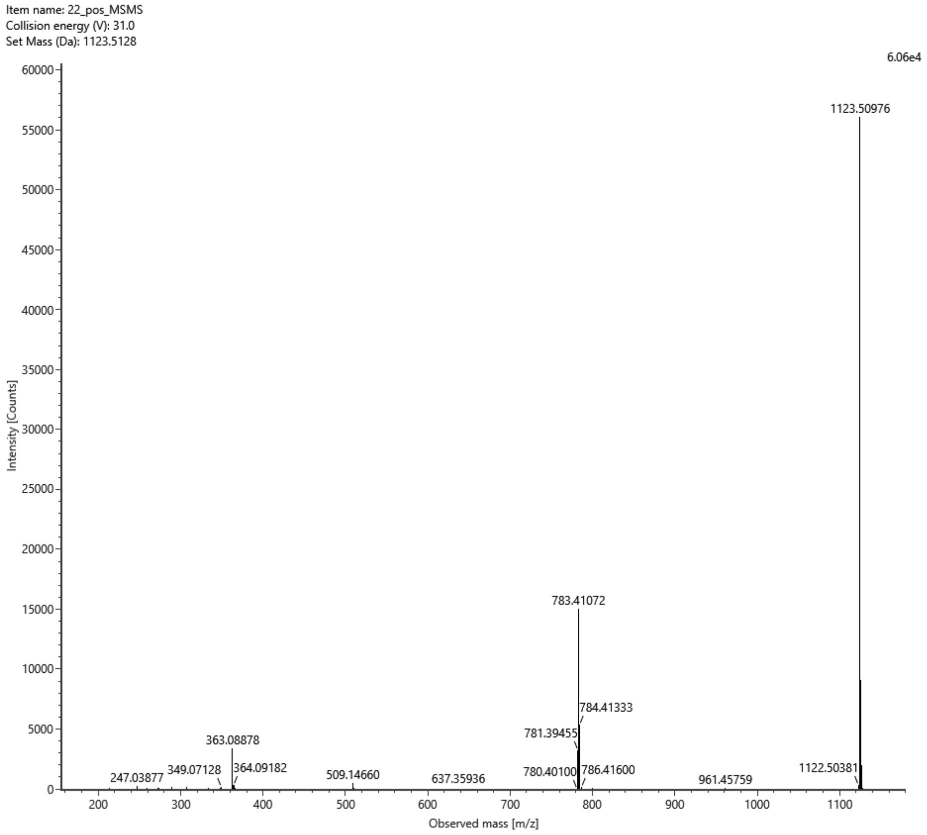

MS/MS spectrum for the compound Capsianoside-III
ESI-MS mode: negative

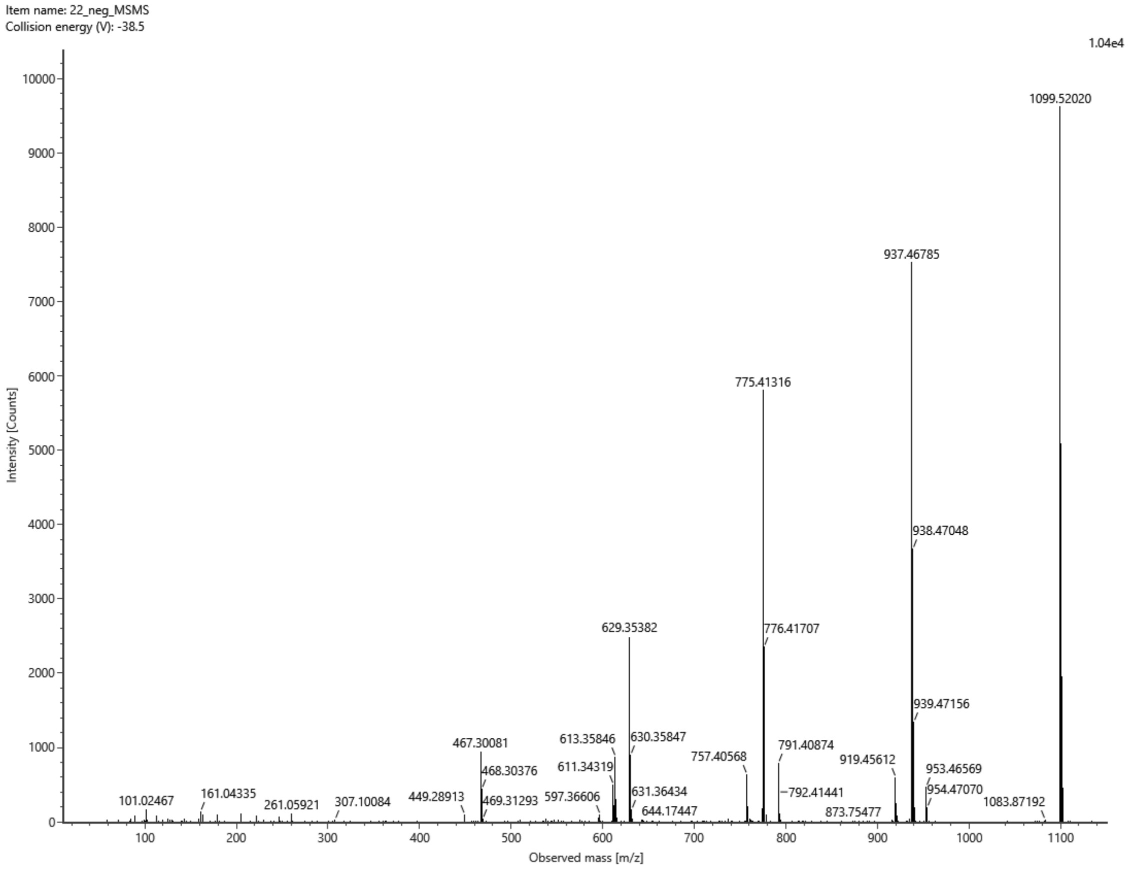

MS spectrum for the diglucoside of Capsianoside-IV (Na^+ adduct)
ESI-MS mode: positive, Unispray ion source

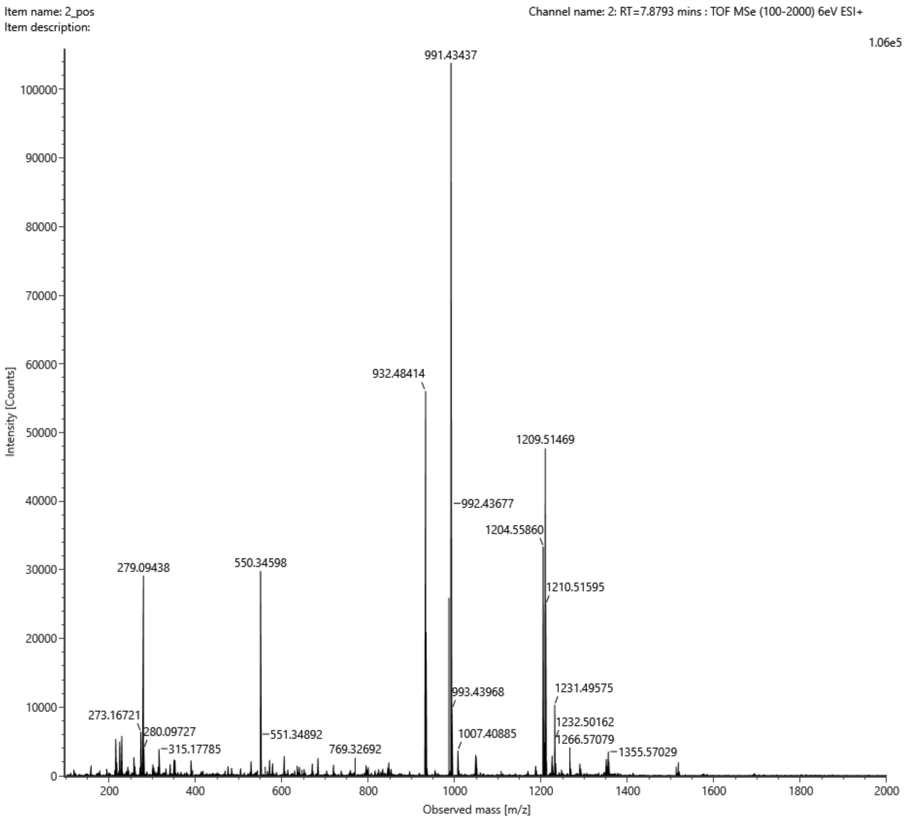

MS/MS spectrum for the diglucoside of Capsianoside-IV (Na^+ adduct)
ESI-MS mode: positive, Unispray ion source

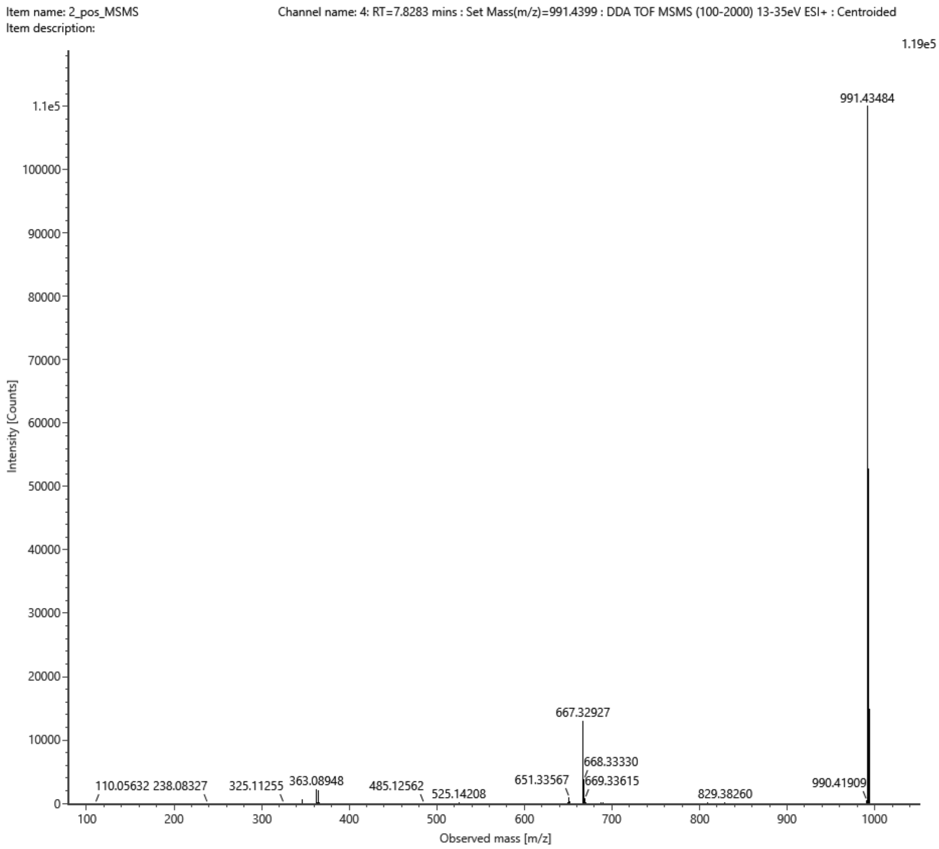

MS spectrum for the diglucoside of Capsianoside-IV
ESI-MS mode: negative, Unispray ion source

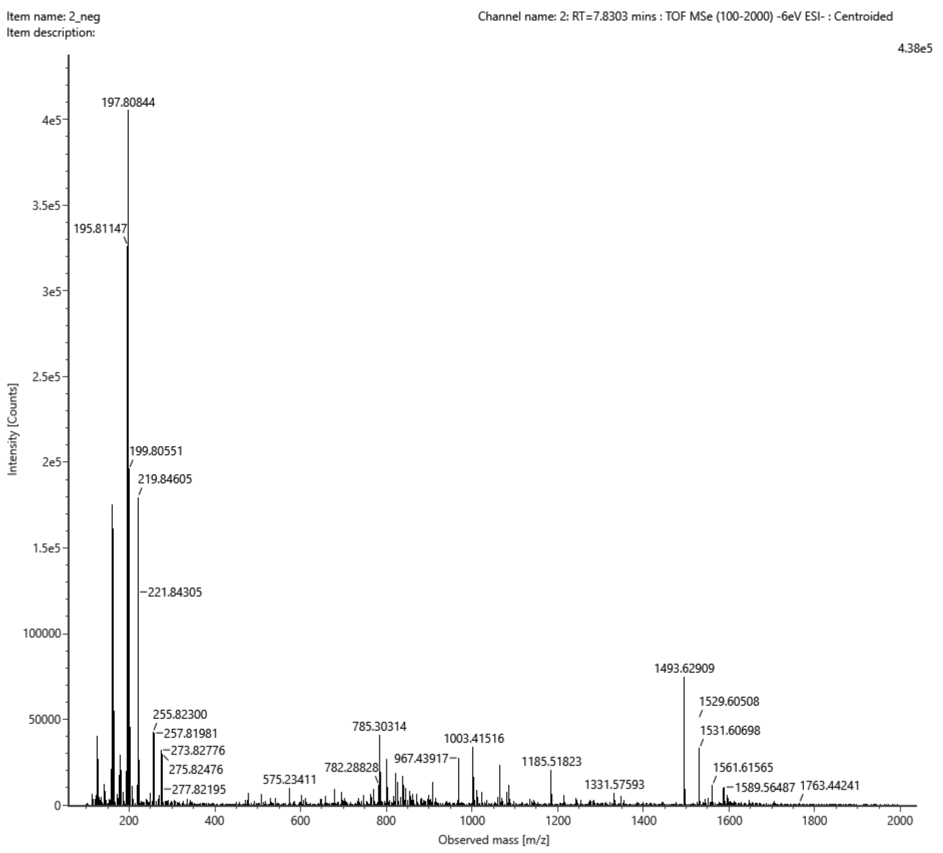

MS/MS spectrum for the diglucoside of Capsianoside-IV
ESI-MS mode: negative, Unispray ion source

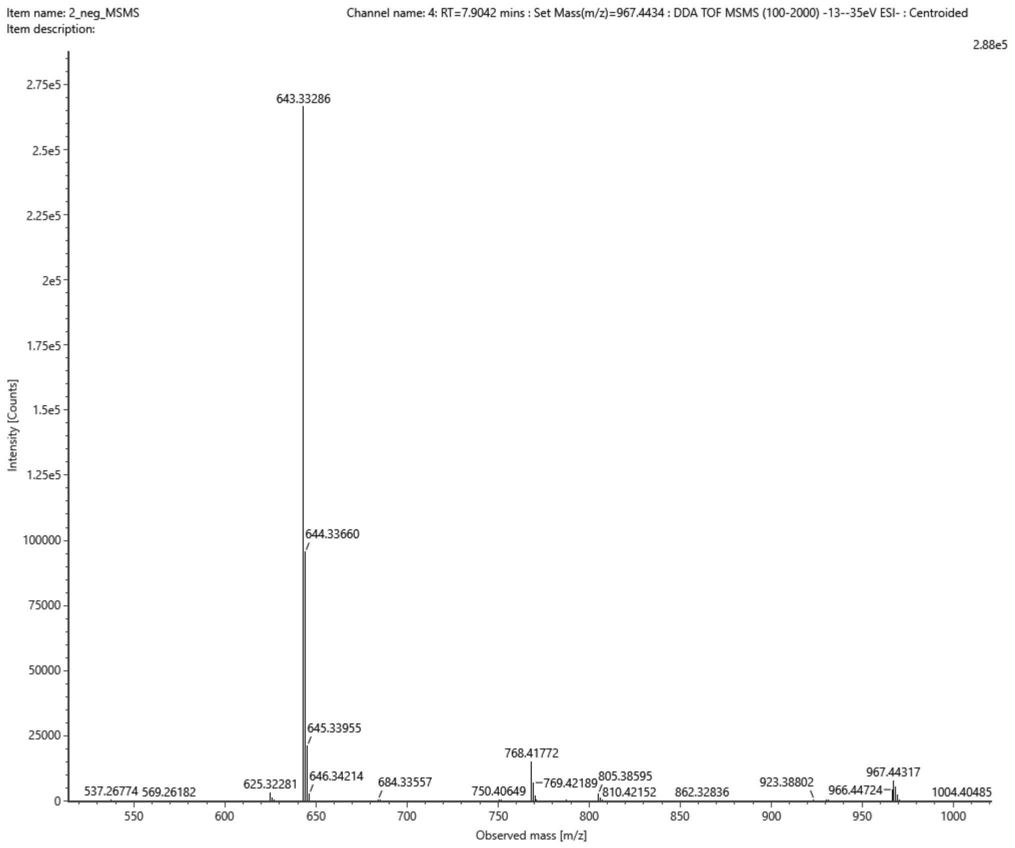

MS spectrum for Capsianoside-V (Na⁺ adduct)
ESI-MS mode: positive, Unispray ion source

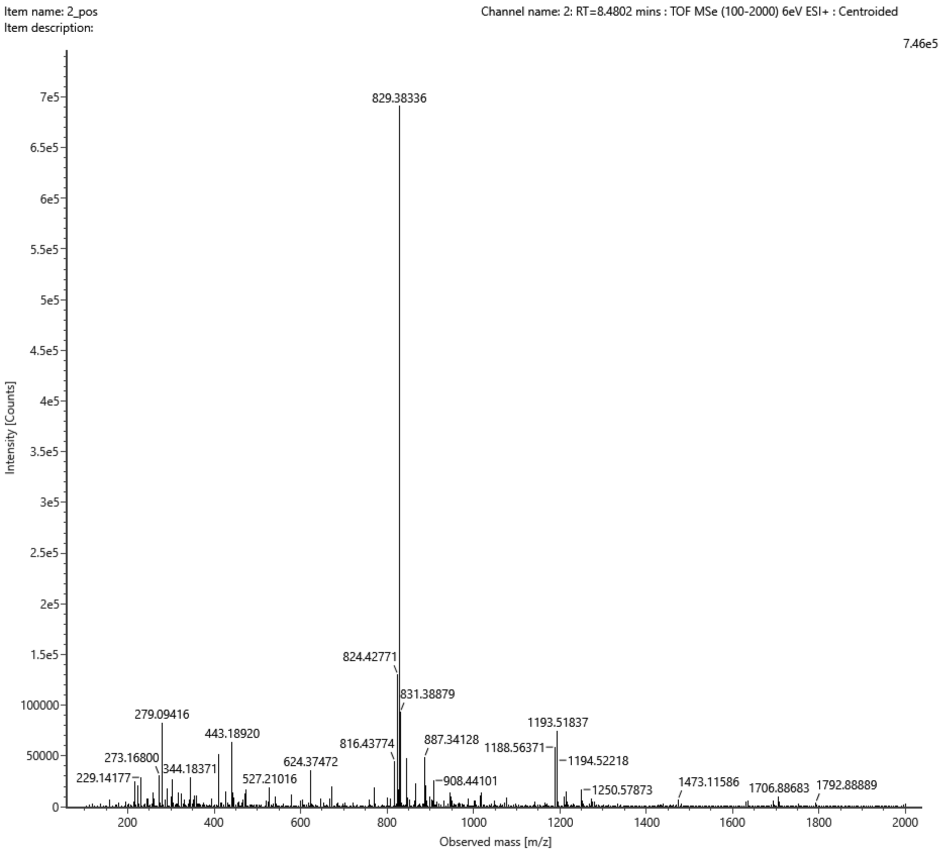

MS spectrum for Capsianoside-V
ESI-MS mode: negative, Unispray ion source

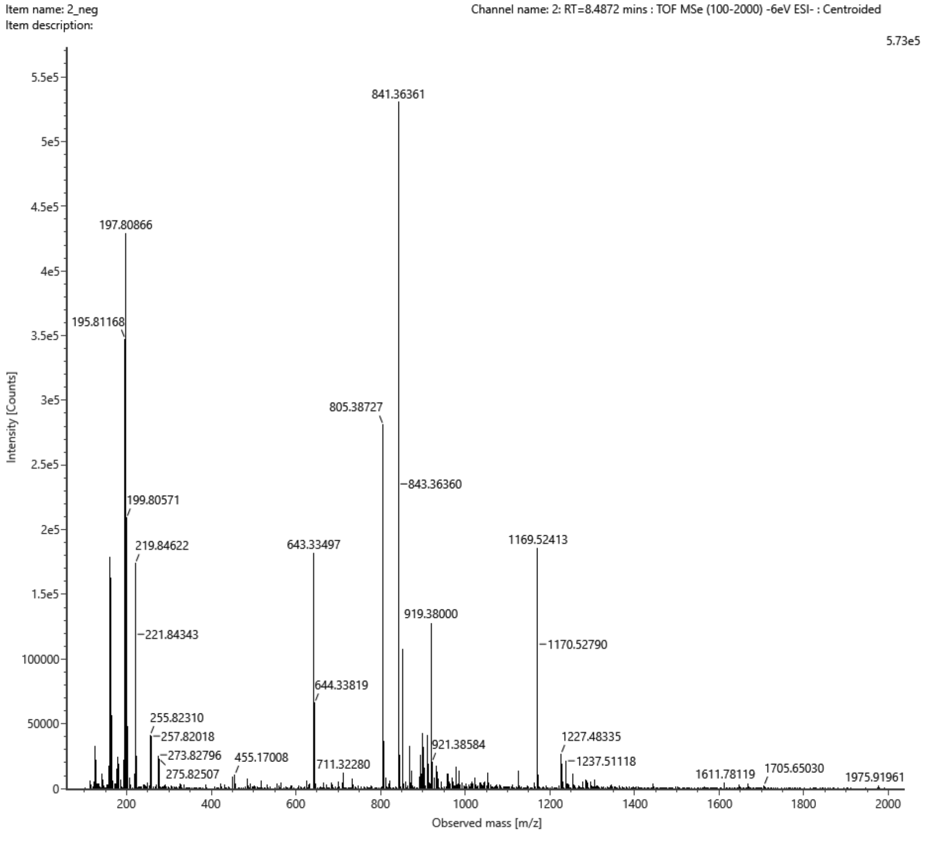

MS/MS spectrum for Capsianoside-V
ESI-MS mode: positive

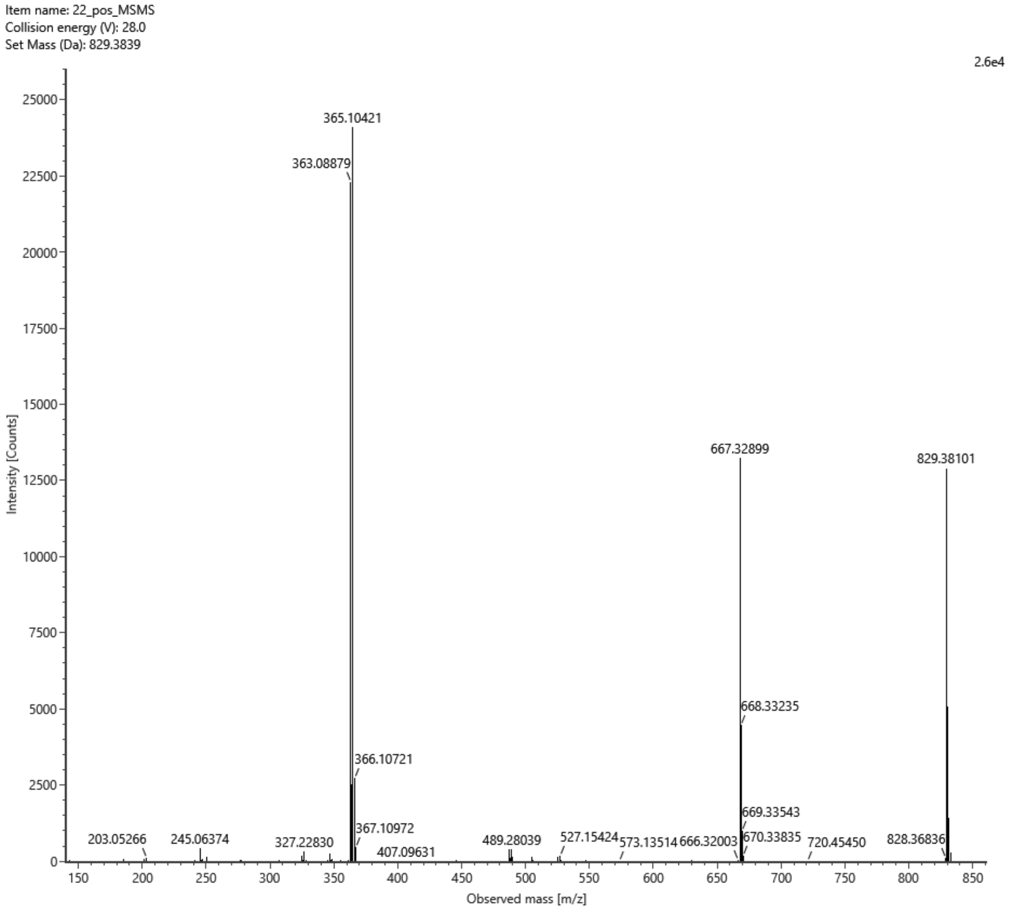

MS/MS spectrum for Capsianoside-V
ESI-MS mode: negative *(note the low abundance of the parent ion at m/z 805)*

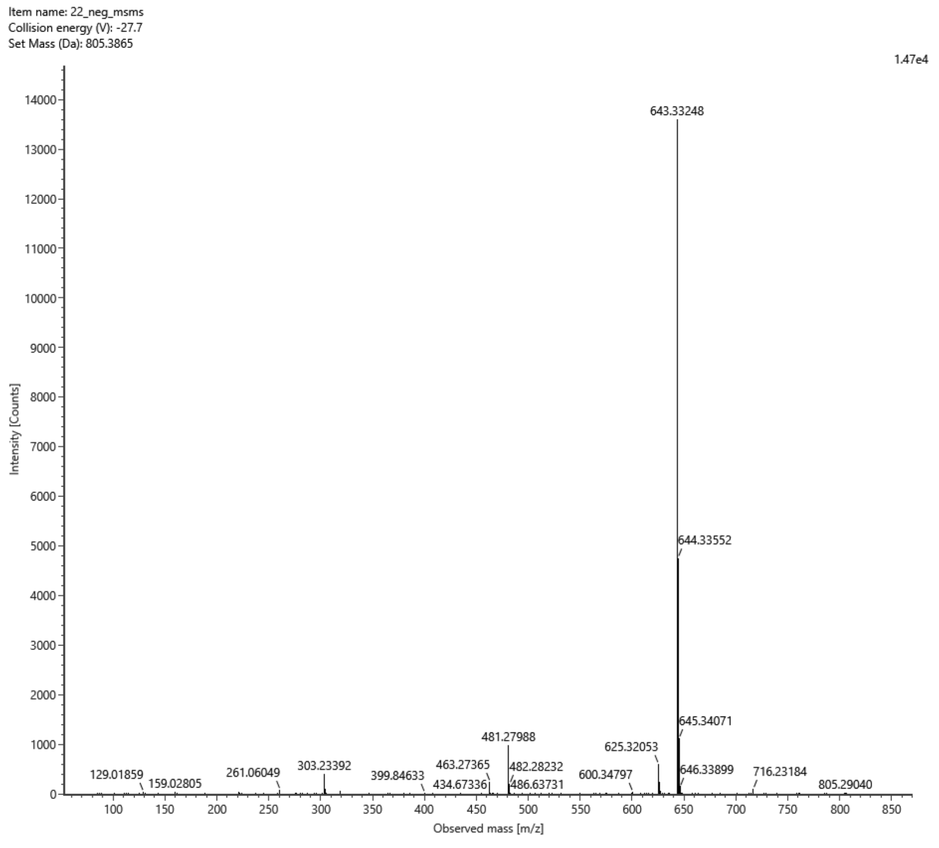

MS spectrum for the compound NI1
ESI-MS mode: positive, Unispray ion source

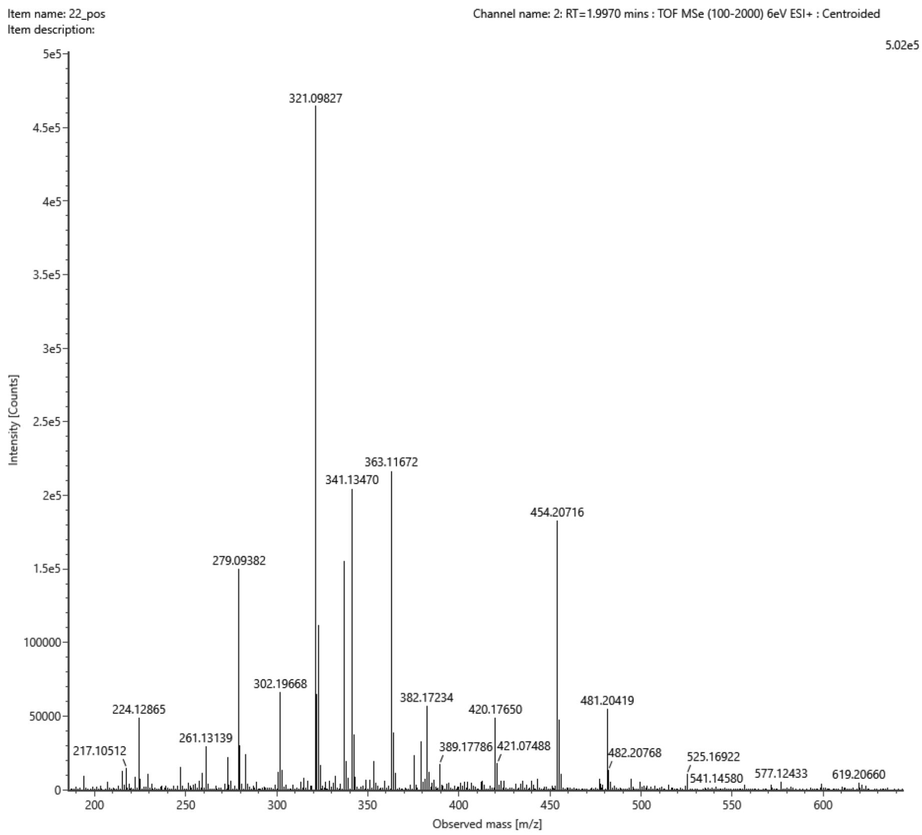

MS/MS spectrum for the compound NI1
ESI-MS mode: positive *(note the low abundance of the parent ion at m/z 341)*

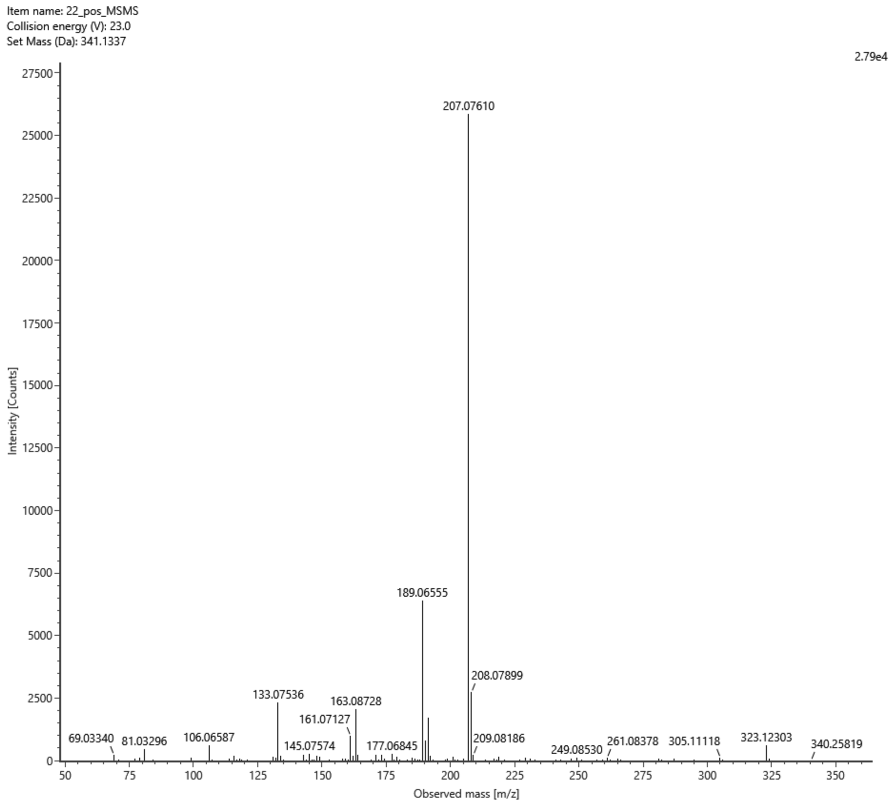

MS spectrum for the compound NI2
ESI-MS mode: positive, Unispray ion source

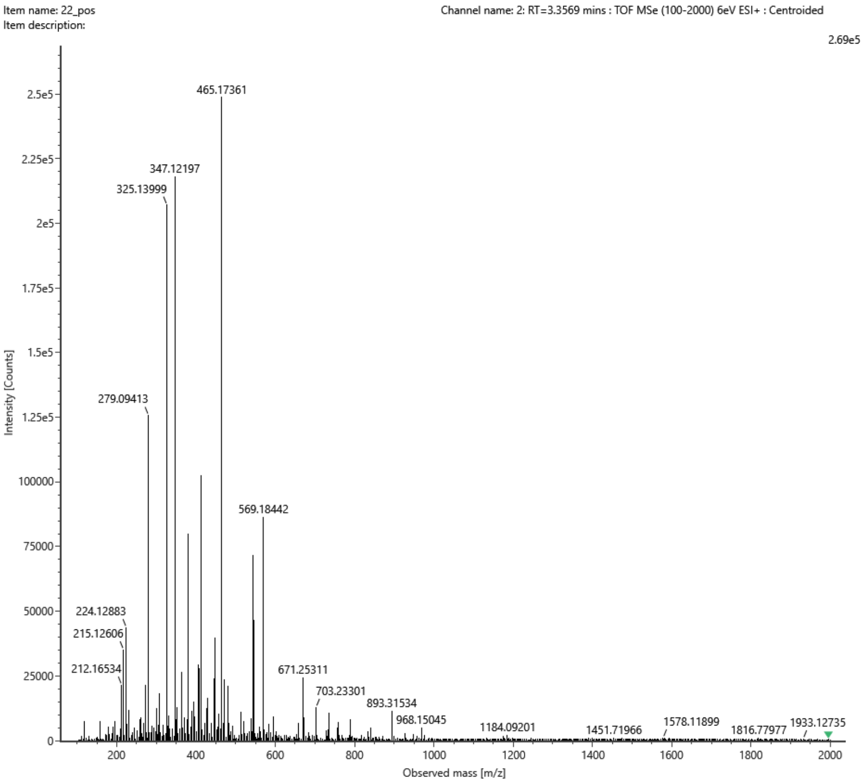

MS/MS spectrum for the compound NI2
ESI-MS mode: positive, Unispray ion source

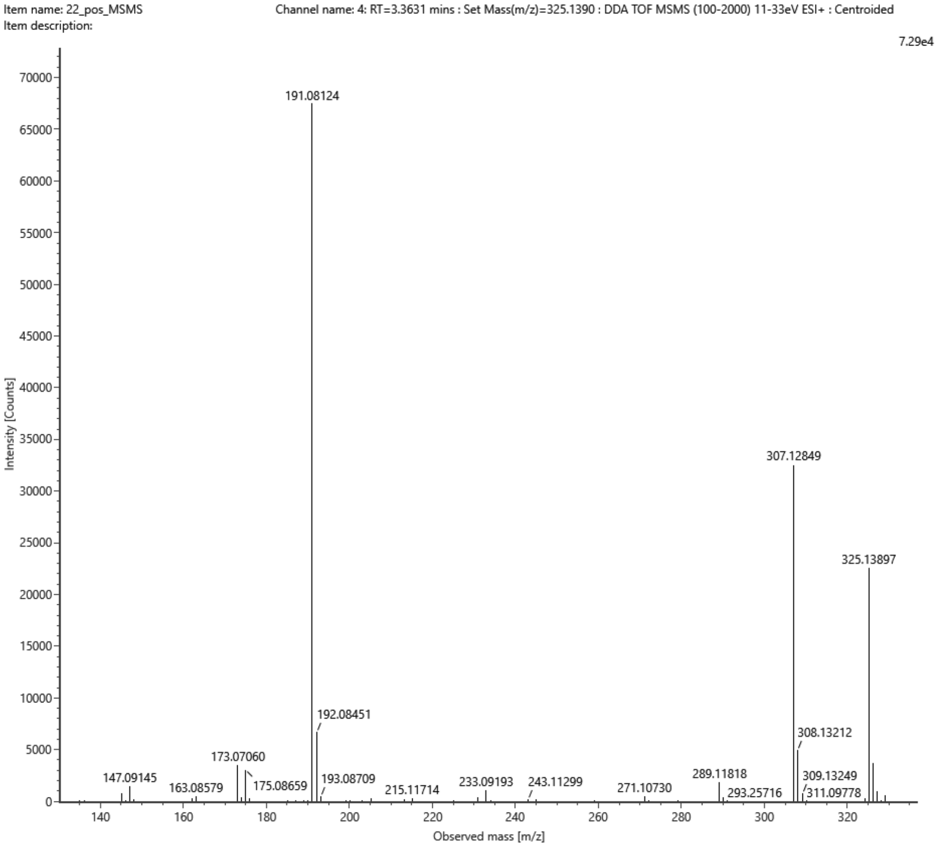

MS spectrum for the compound NI3
ESI-MS mode: positive, Unispray ion source

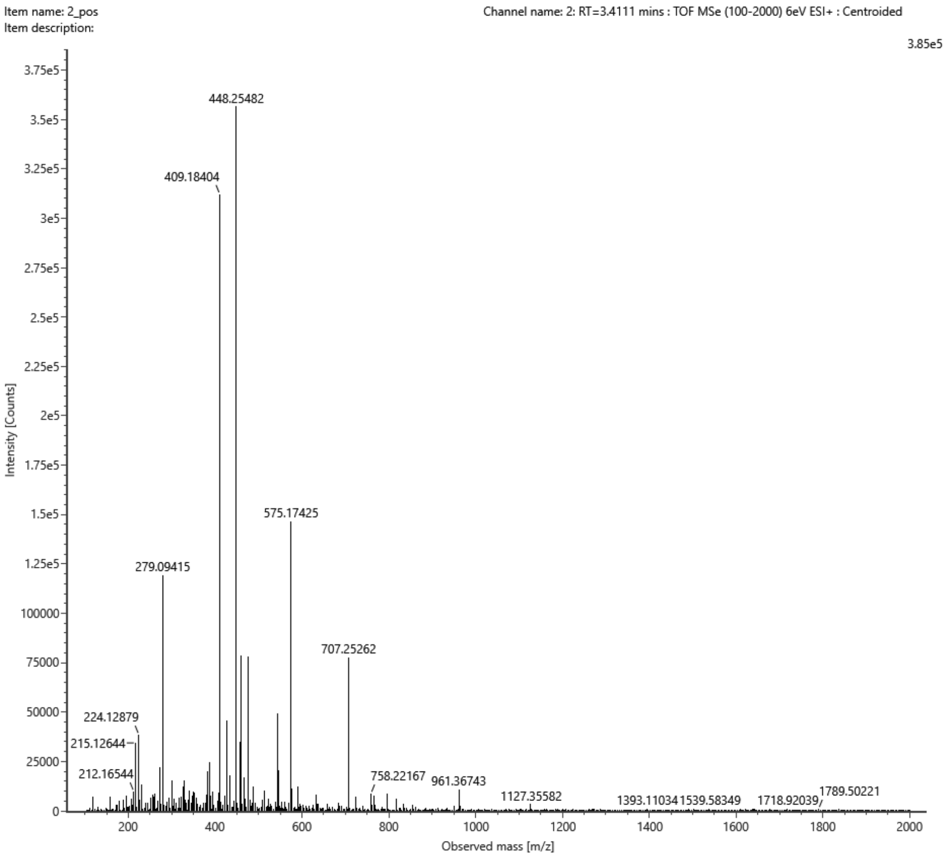

MS/MS spectrum for the compound NI3 (Na^+ adduct)
ESI-MS mode: positive, Unispray ion source

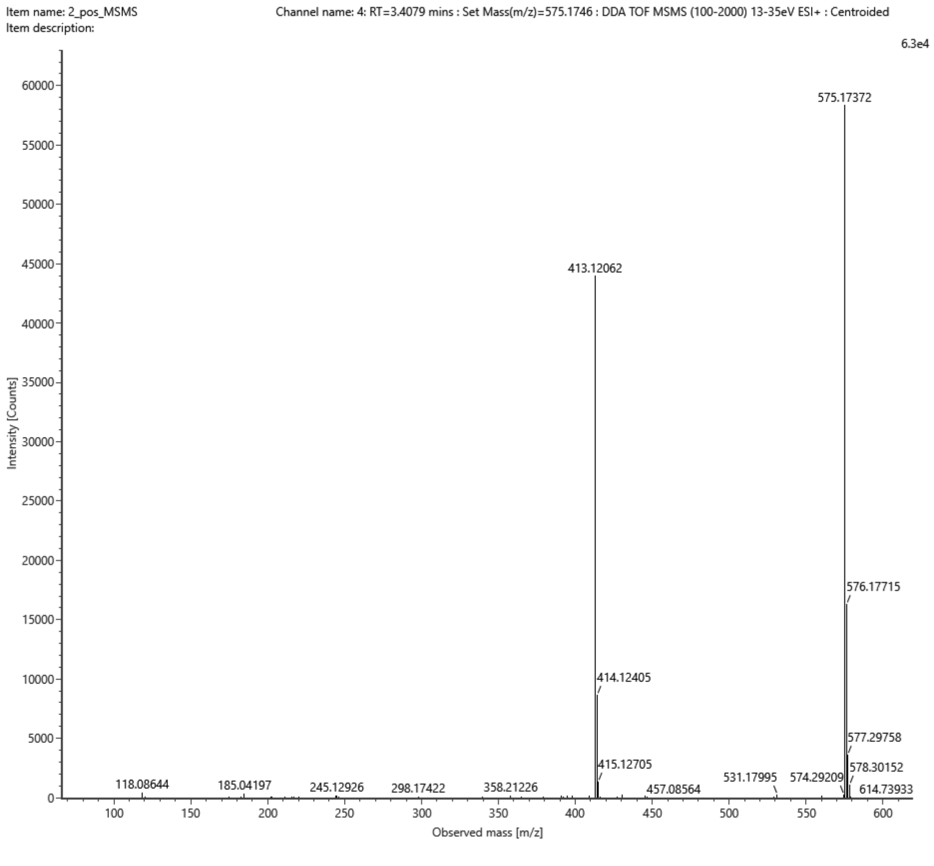

MS spectrum for the compound NI6 (supposed NCC_840 phyllobilin isomer)
ESI-MS mode: positive, Unispray ion source

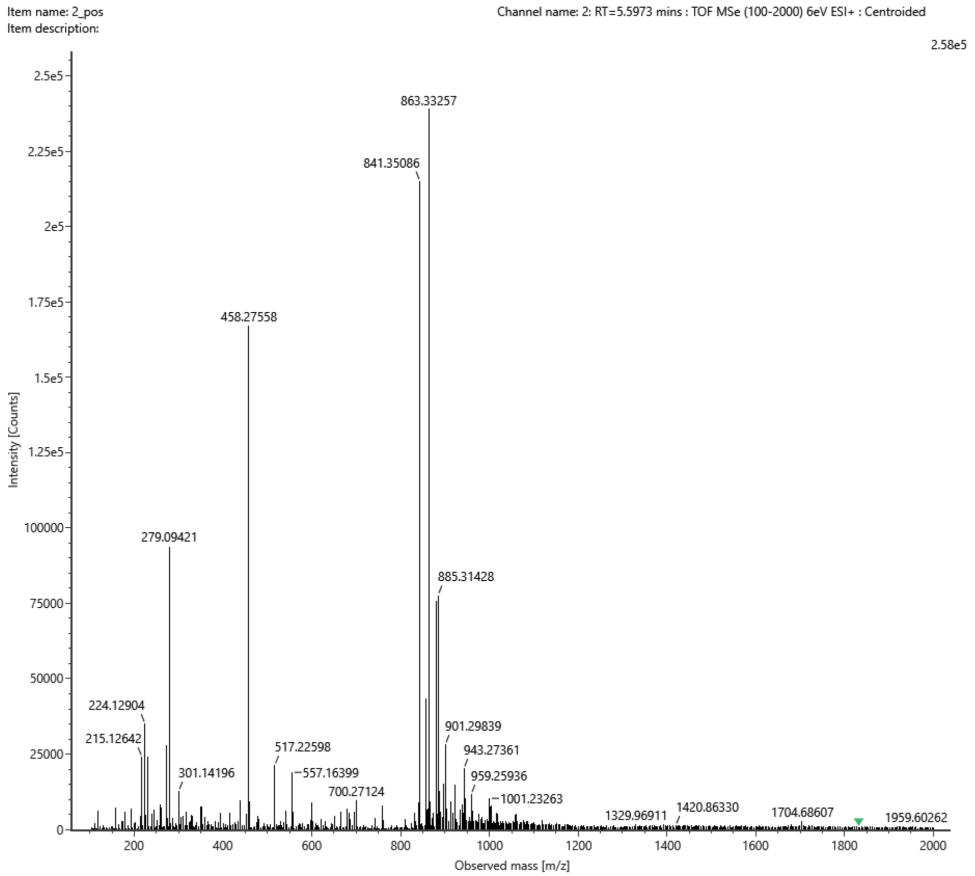

MS/MS spectrum for the compound NI6 (supposed NCC_840 phyllobilin isomer)
ESI-MS mode: positive, Unispray ion source

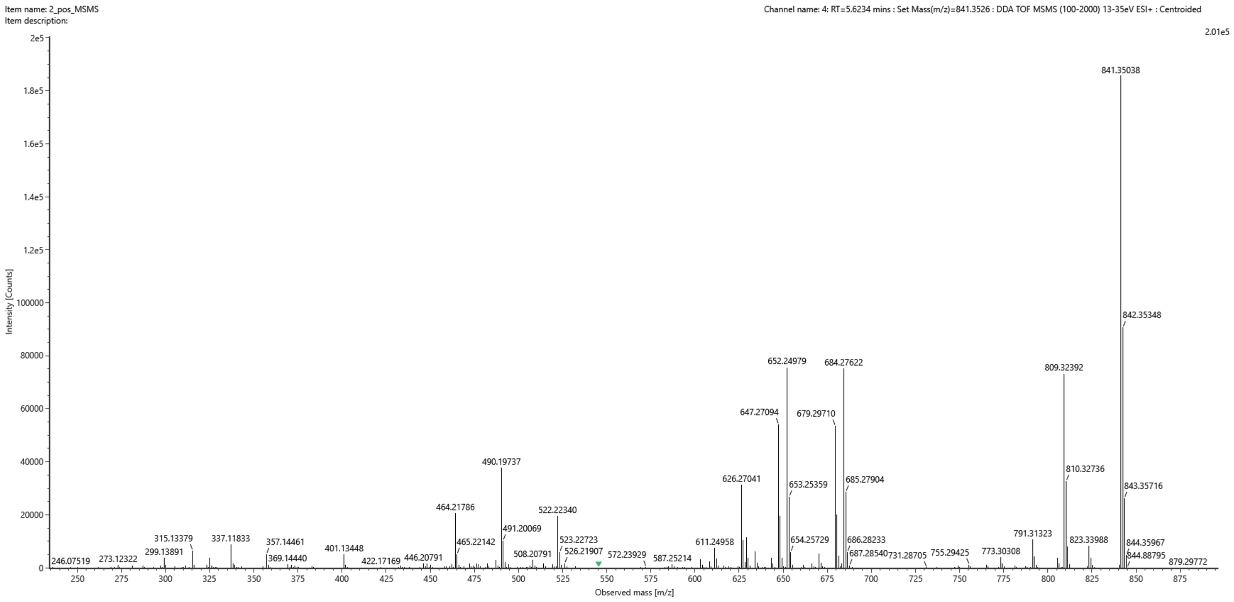

MS spectrum for the compound NI7 (supposed NCC_840 phyllobilin isomer)
ESI-MS mode: positive, Unispray ion source

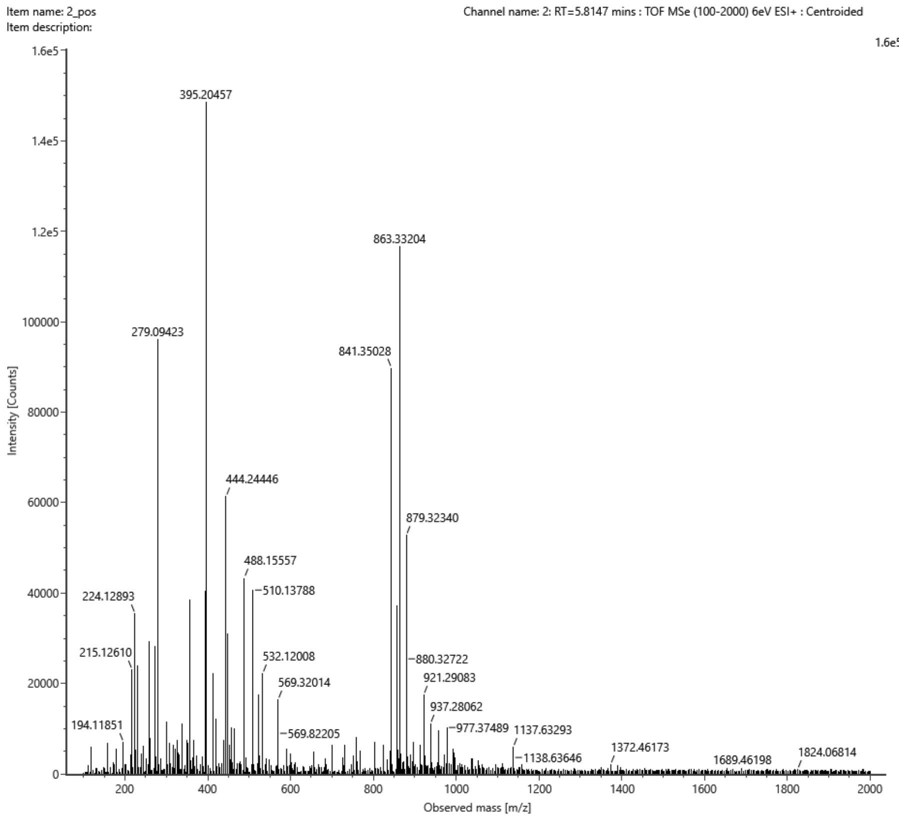

MS/MS spectrum for the compound NI7 (supposed NCC_840 phyllobilin isomer)
ESI-MS mode: positive, Unispray ion source

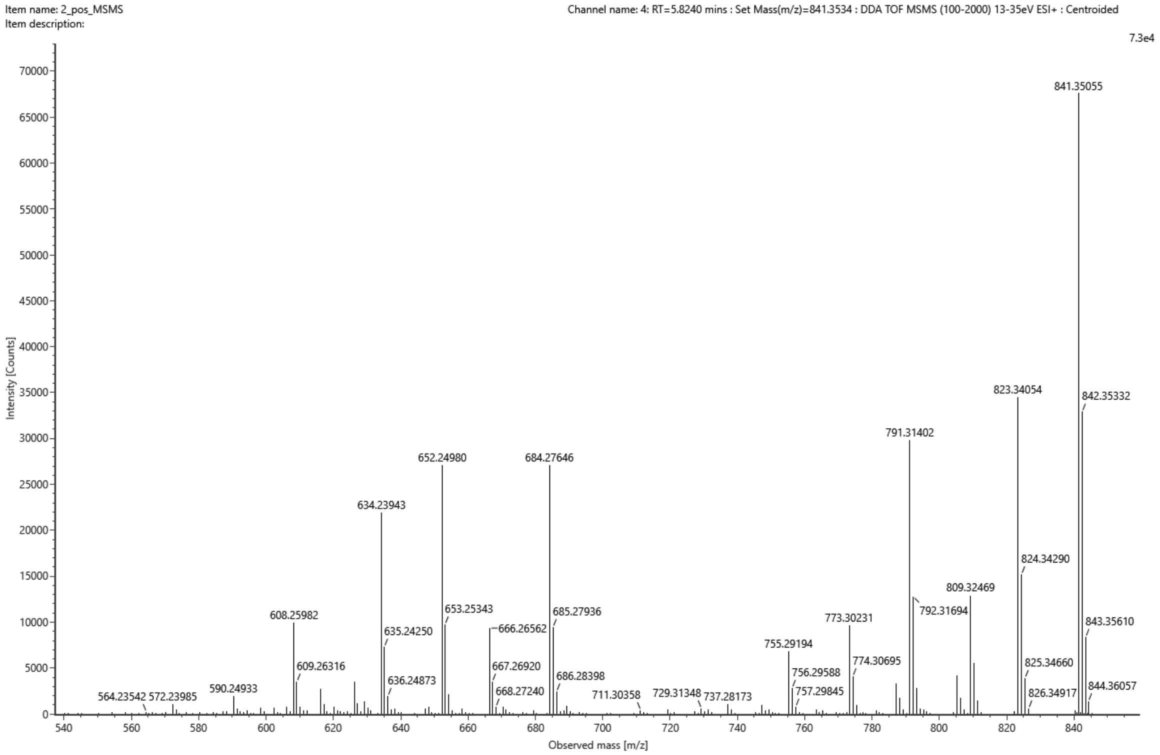

MS spectrum for the compound NI9 (supposed NCC_678 phyllobilin isomer)
ESI-MS mode: positive, Unispray ion source

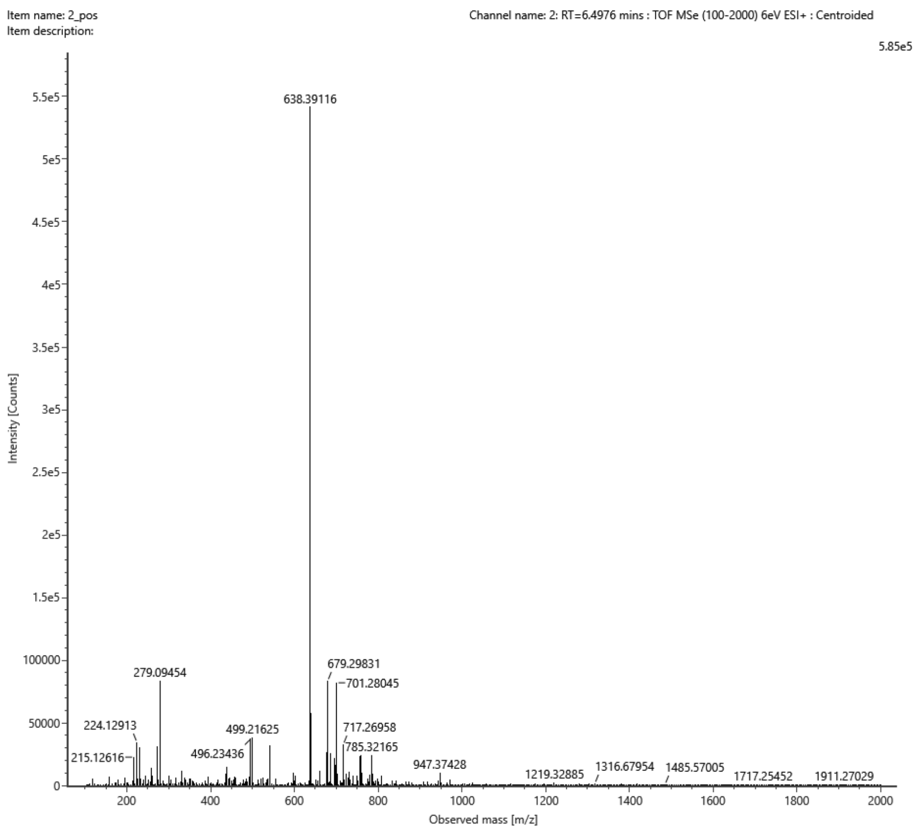

MS spectrum for the compound NI10 (supposed NCC_678 phyllobilin isomer)
ESI-MS mode: positive, Unispray ion source

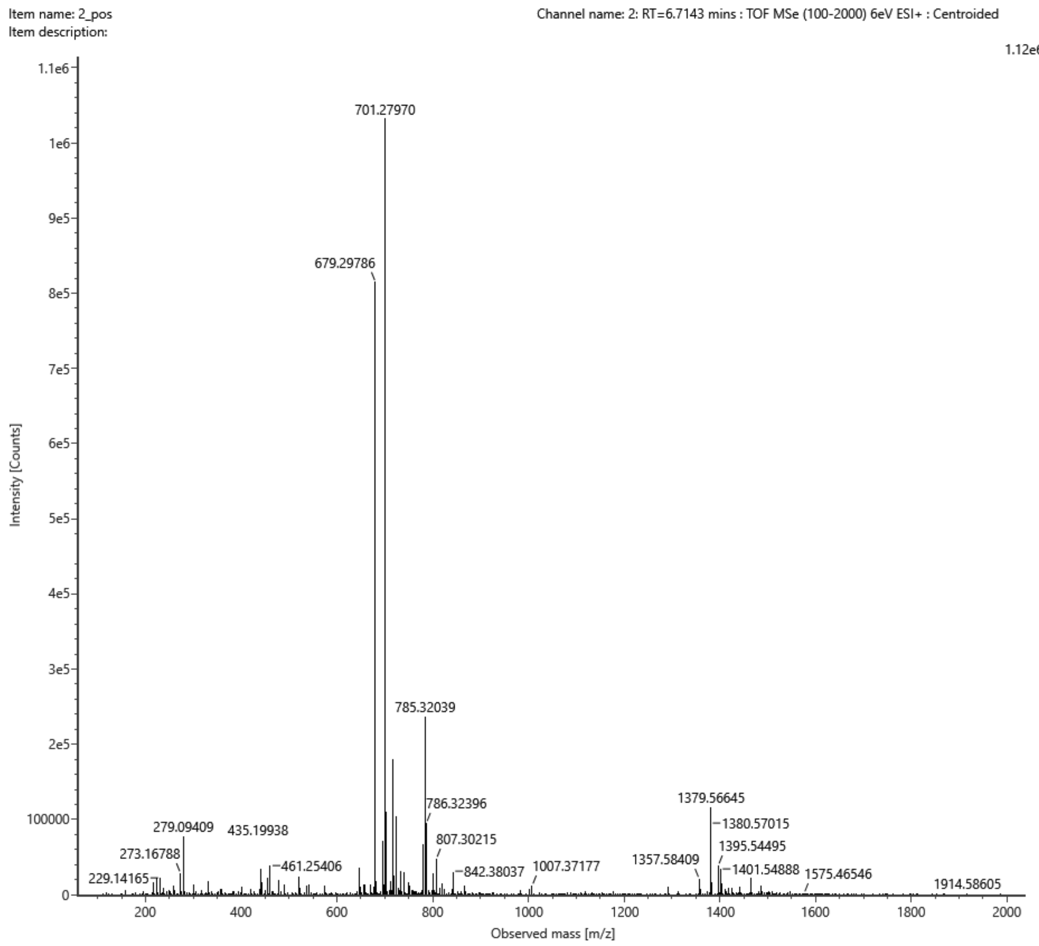

MS/MS spectrum for the compound NI10 (supposed NCC_678 phyllobilin isomer)
ESI-MS mode: positive, Unispray ion source

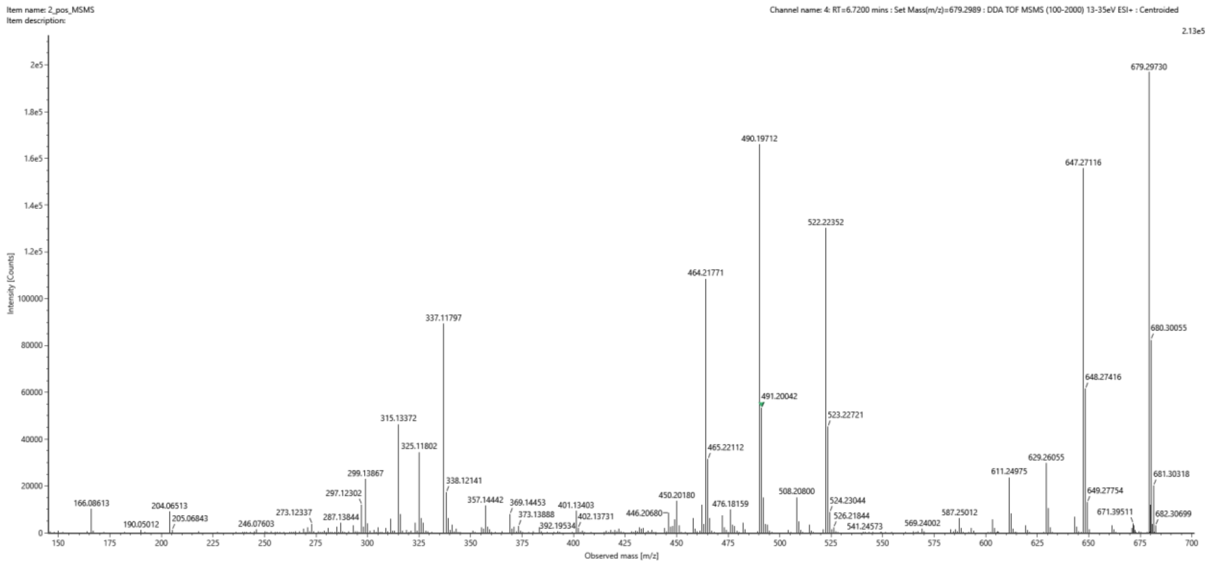

MS spectrum for the compound NI11 (supposed NCC_678 phyllobilin isomer)
ESI-MS mode: positive, Unispray ion source

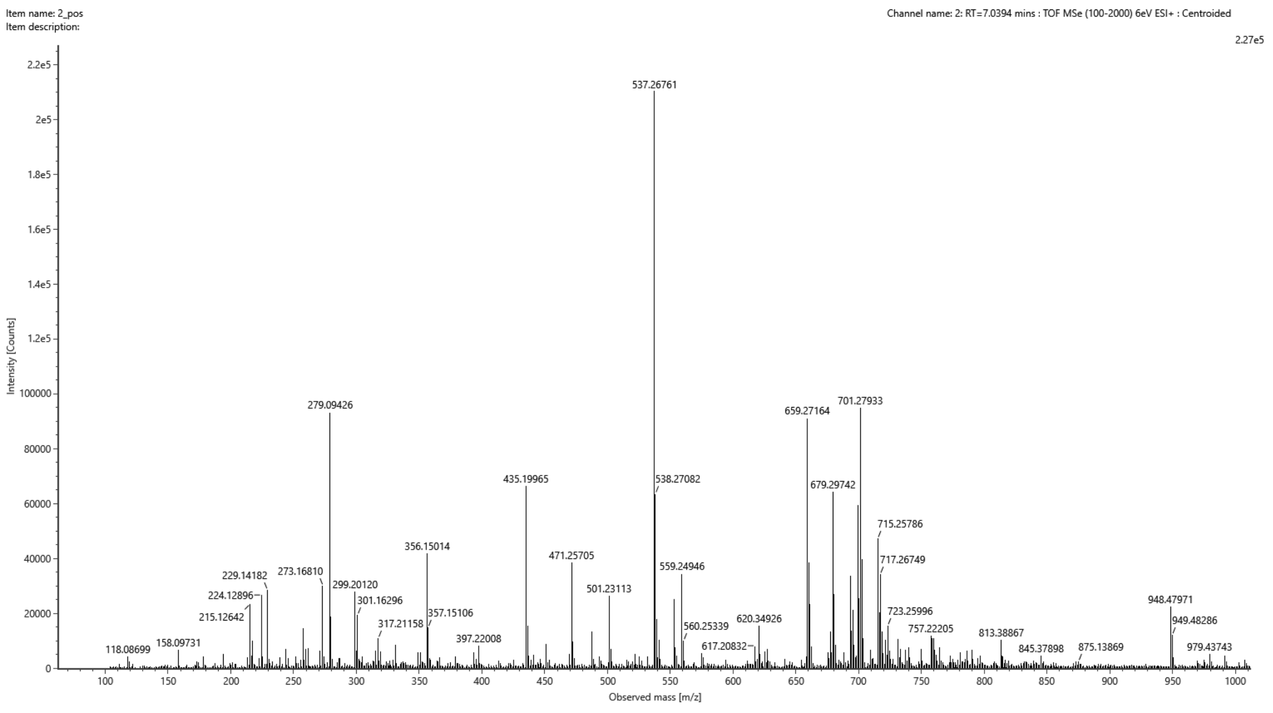

MS spectrum for the compound NI12 (supposed Capsianoside-IX; Na^+ adduct)
ESI-MS mode: positive, Unispray ion source

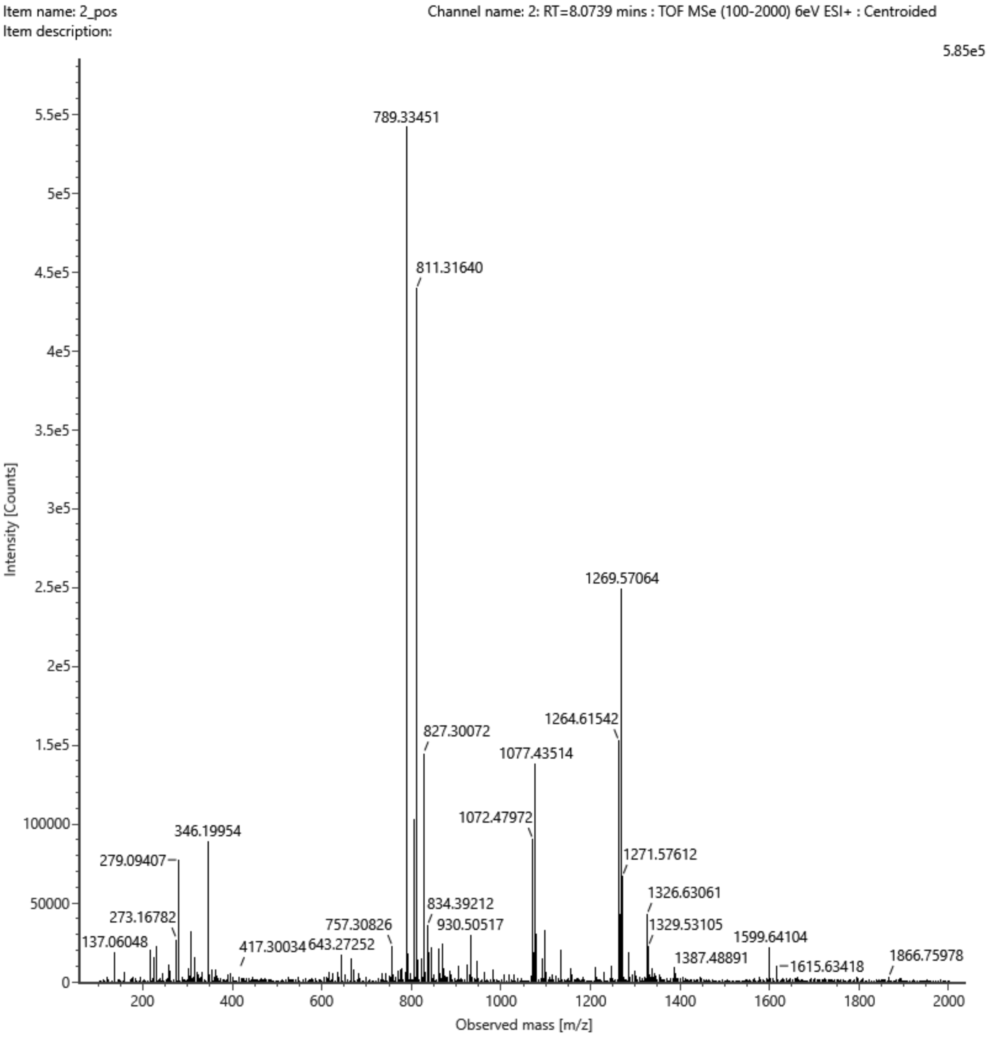

MS spectrum for the compound NI14 (supposed Capsianoside monomer, Na$^+$ adduct)
ESI-MS mode: positive, Unispray ion source

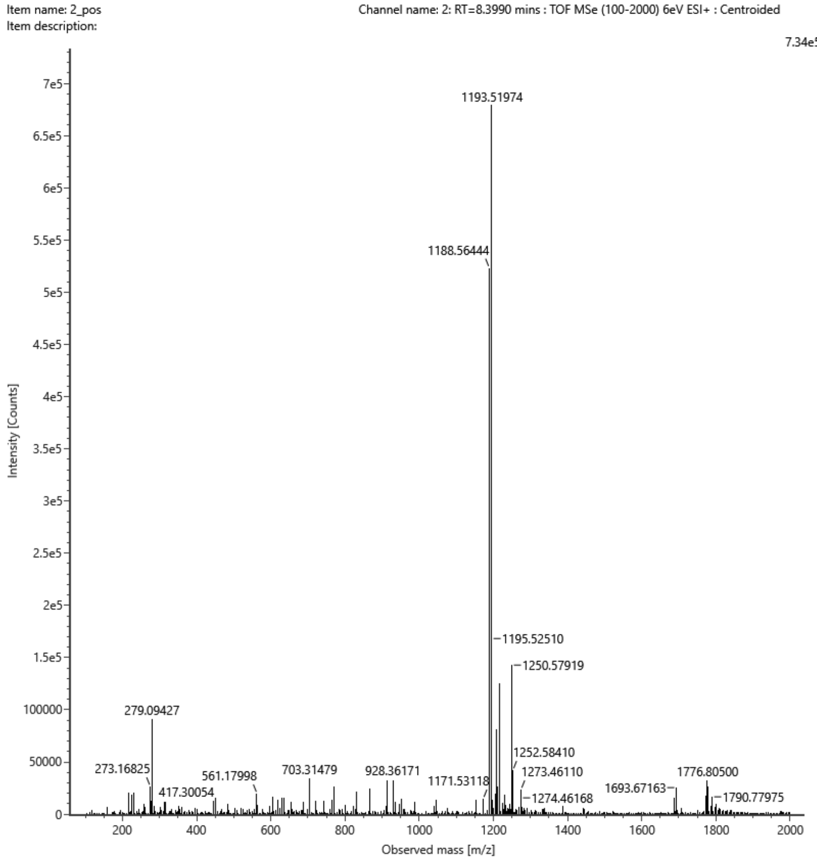

MS/MS spectrum for the compound NI14 (supposed Capsianoside monomer, Na$^+$ adduct)
ESI-MS mode: positive

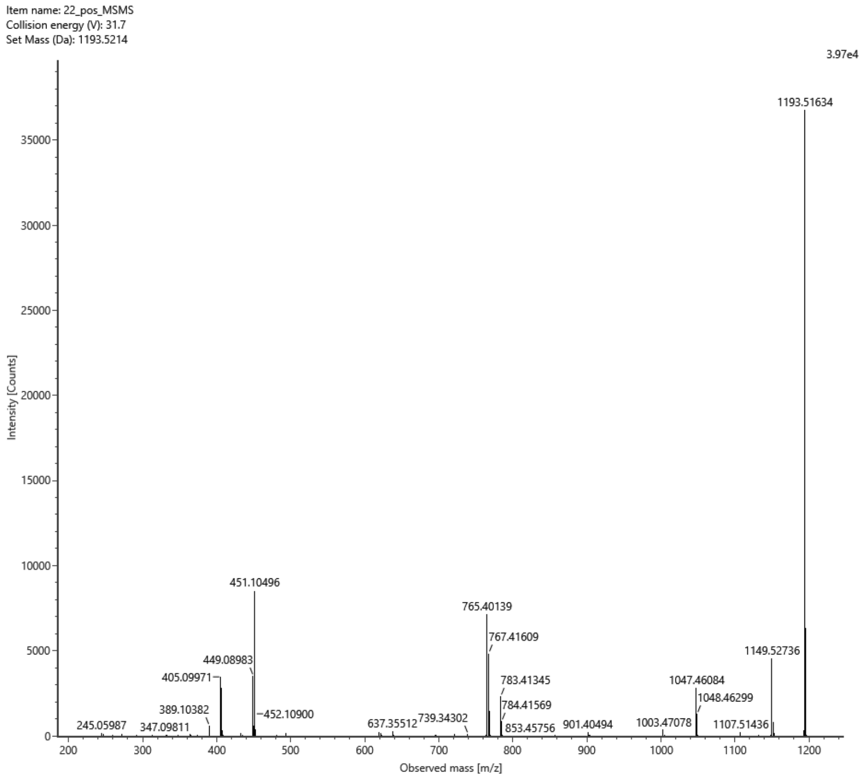

MS spectrum for the compound NI1
ESI-MS mode: negative, Unispray ion source

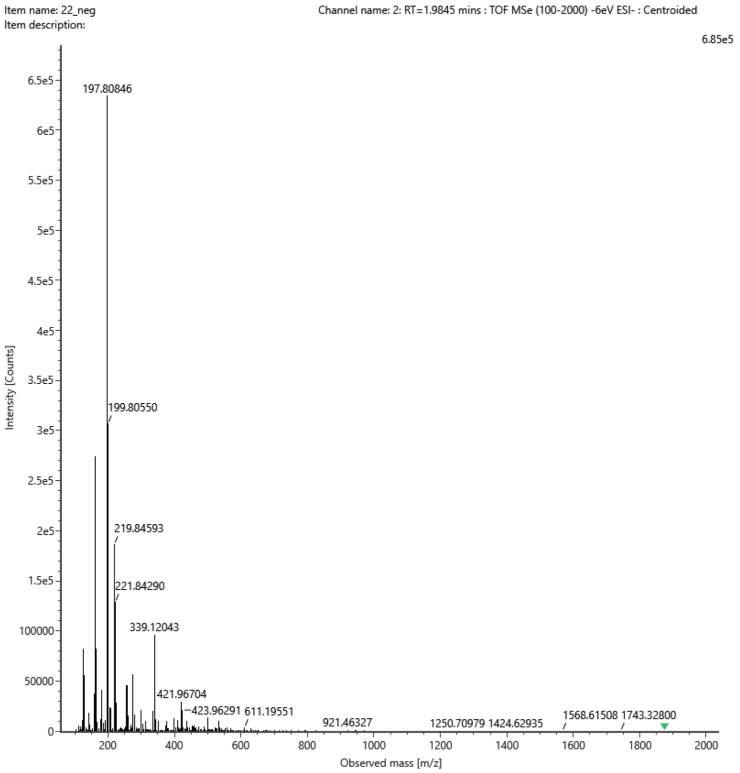

MS/MS spectrum for the compound NI1
ESI-MS mode: negative, Unispray ion source

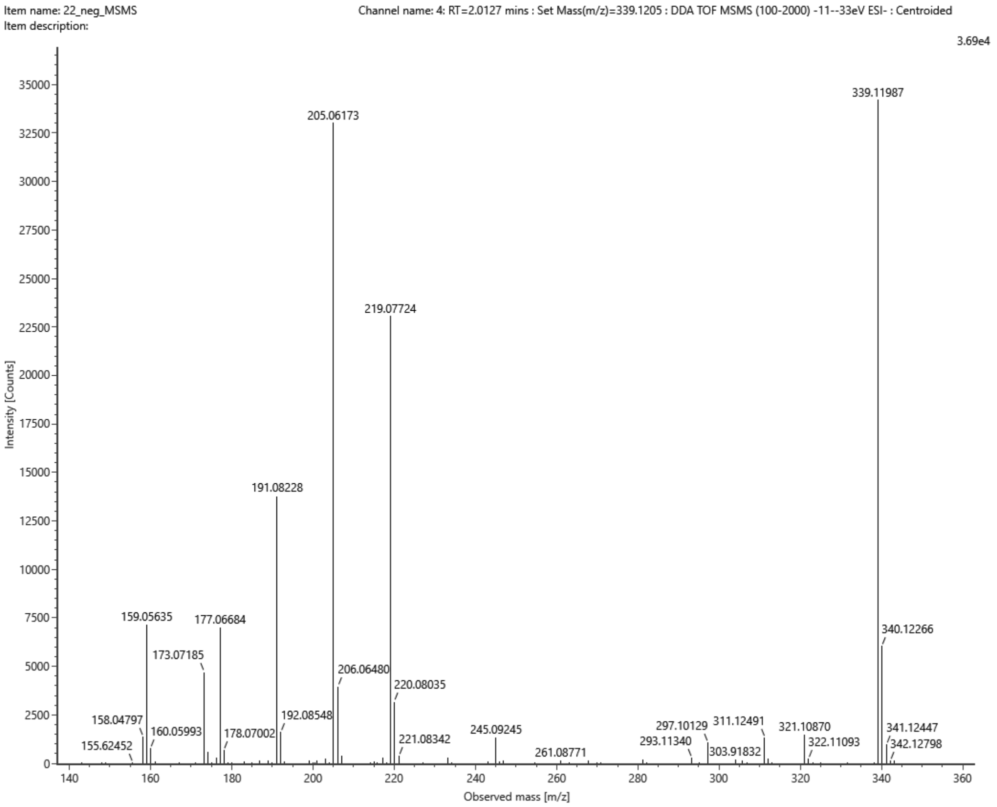

MS spectrum for the compound NI2
ESI-MS mode: negative, Unispray ion source

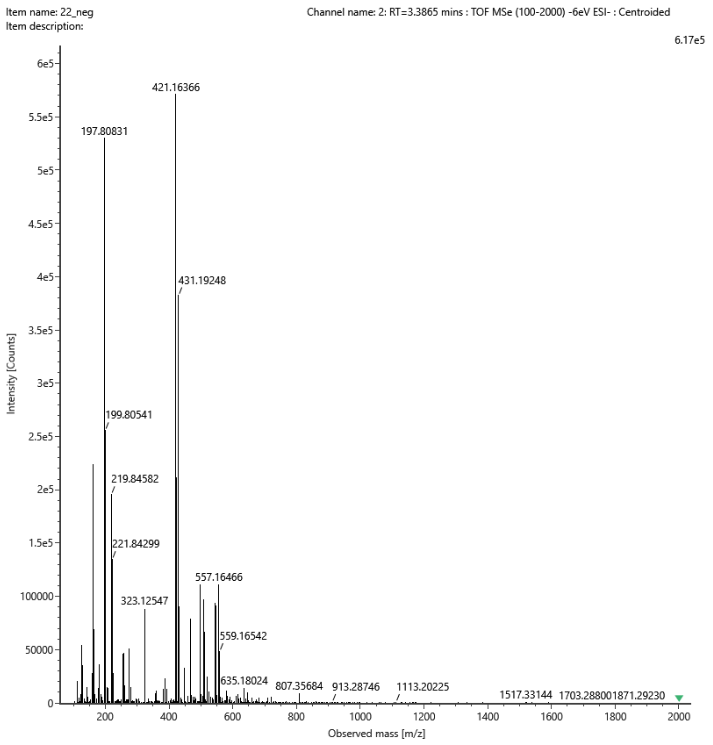

MS/MS spectrum for the compound NI2
ESI-MS mode: negative, Unispray ion source

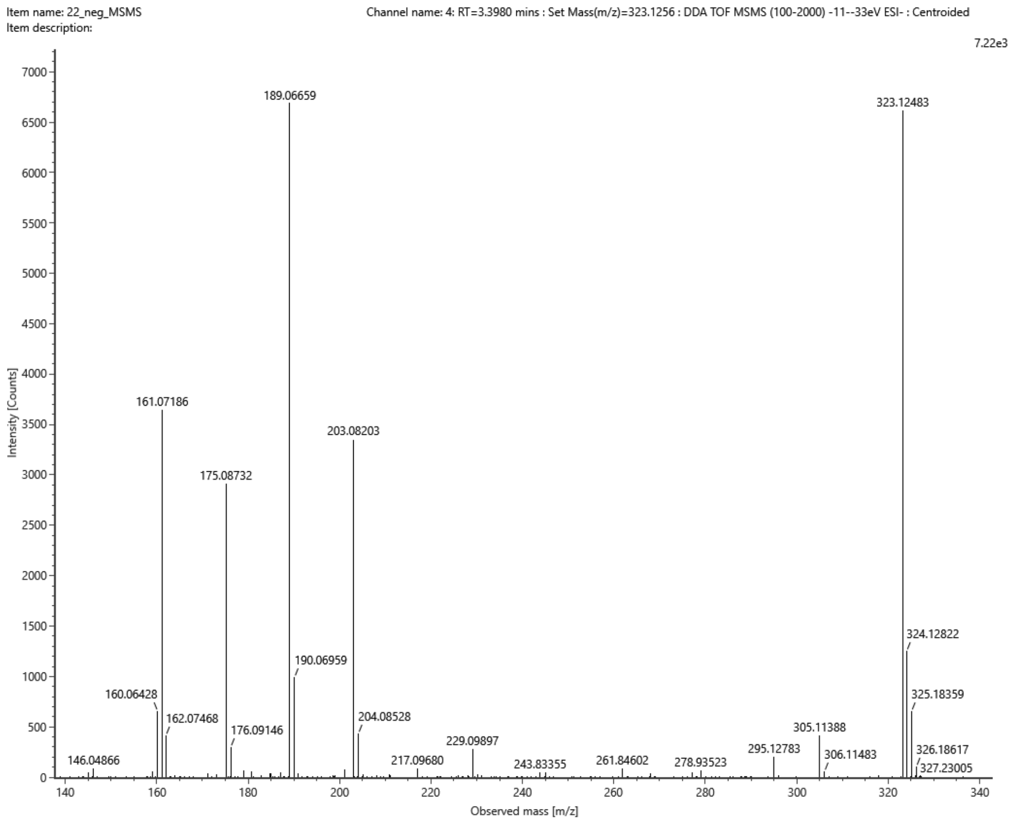

MS spectrum for the compound NI3
ESI-MS mode: negative, Unispray ion source
(no MS/MS data obtained in negative mode)

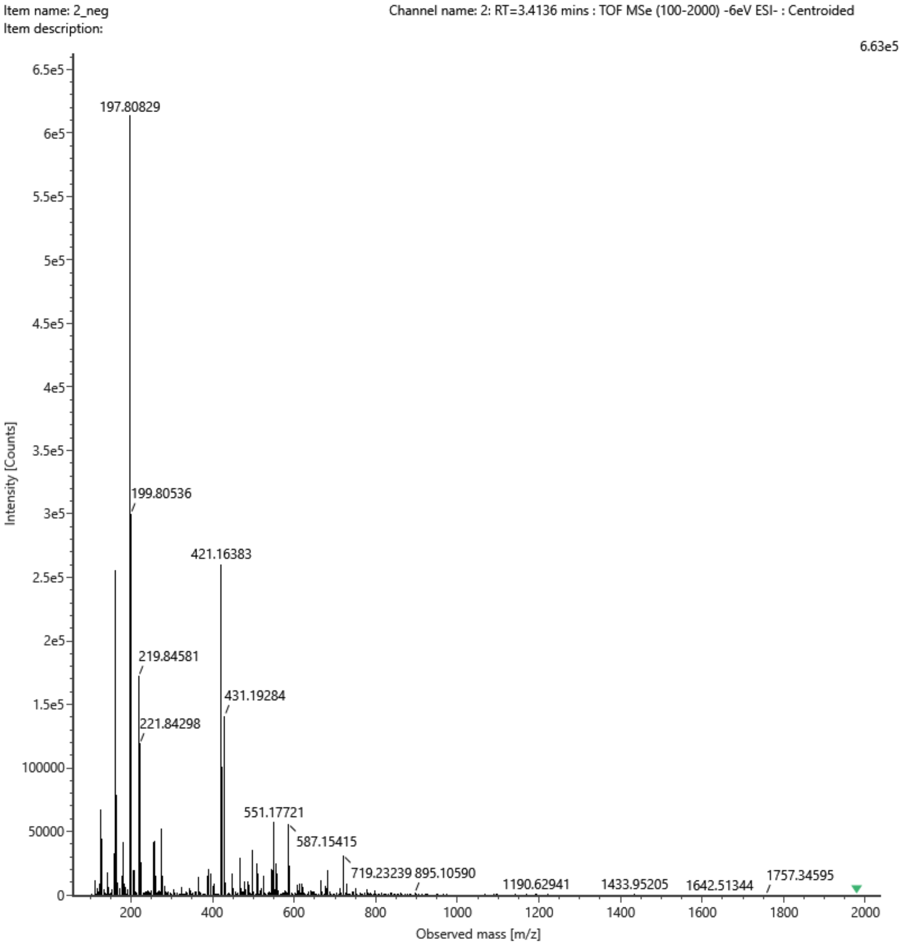

MS spectrum for the compound NI6 (supposed NCC_840 phyllobilin isomer)
ESI-MS mode: negative, Unispray ion source

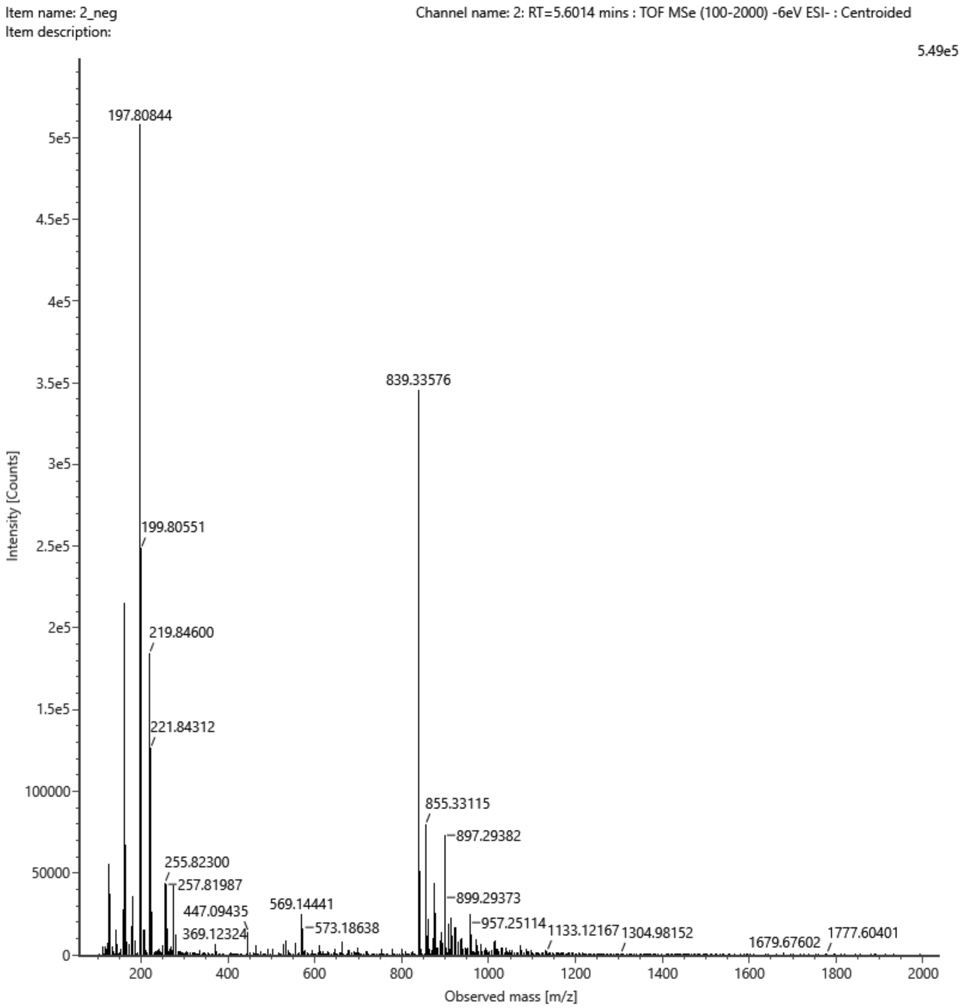

MS/MS spectrum for the compound NI6 (supposed NCC_840 phyllobilin isomer)
ESI-MS mode: negative, Unispray ion source

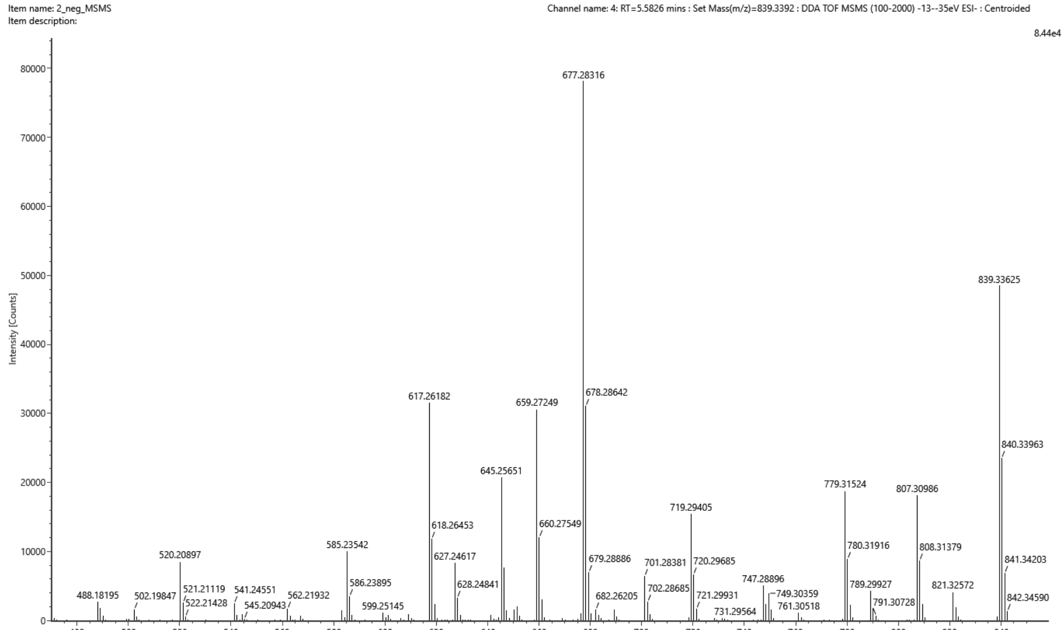

MS spectrum for the compound NI7 (supposed NCC_840 phyllobilin isomer)
ESI-MS mode: negative, Unispray ion source

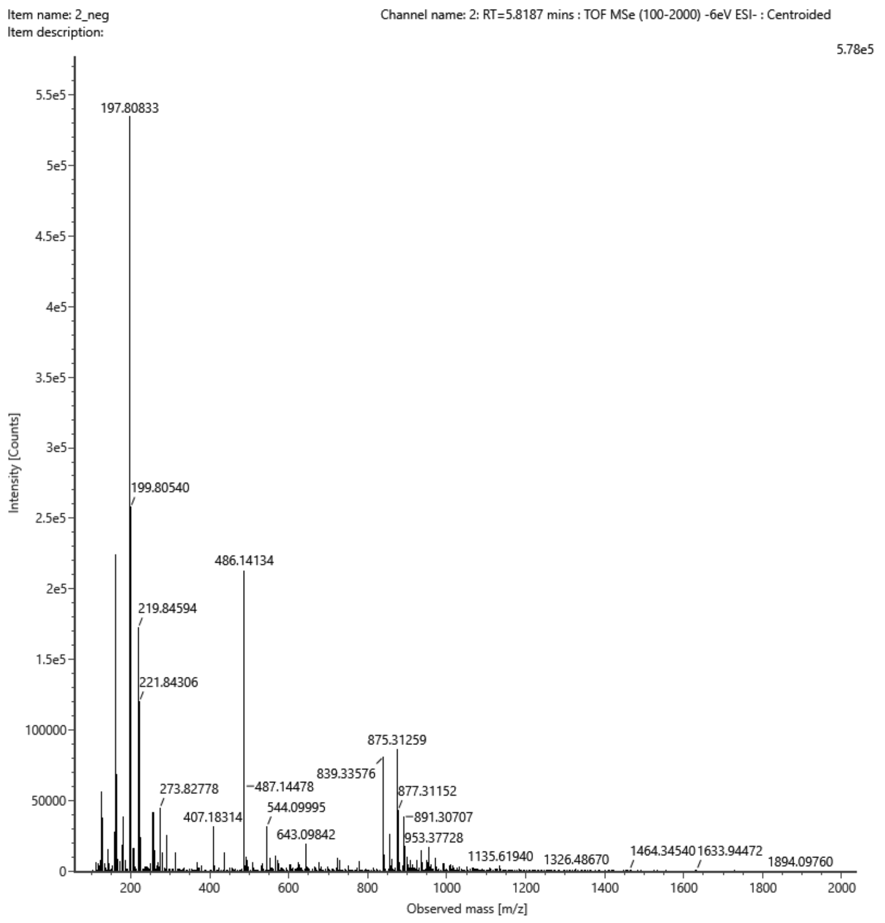

MS/MS spectrum for the compound NI7 (supposed NCC_840 phyllobilin isomer)
ESI-MS mode: negative, Unispray ion source

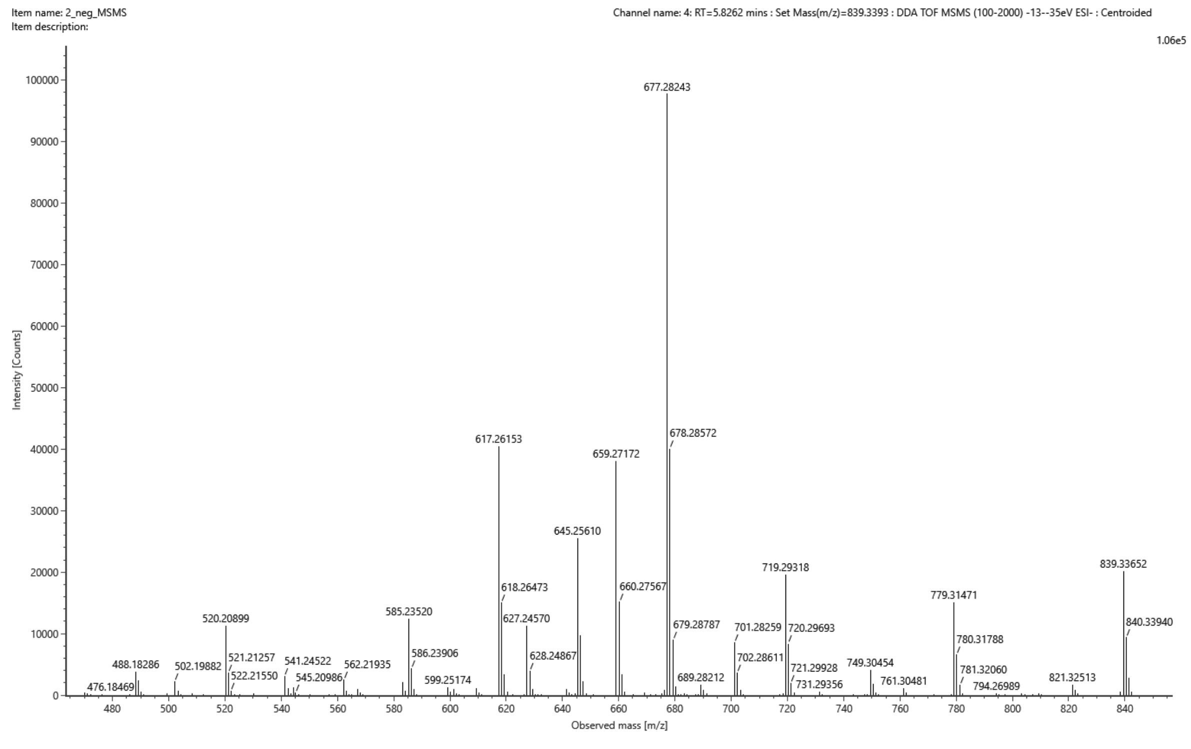

MS spectrum for the compound NI9 (supposed NCC_678 phyllobilin isomer)
ESI-MS mode: negative, Unispray ion source

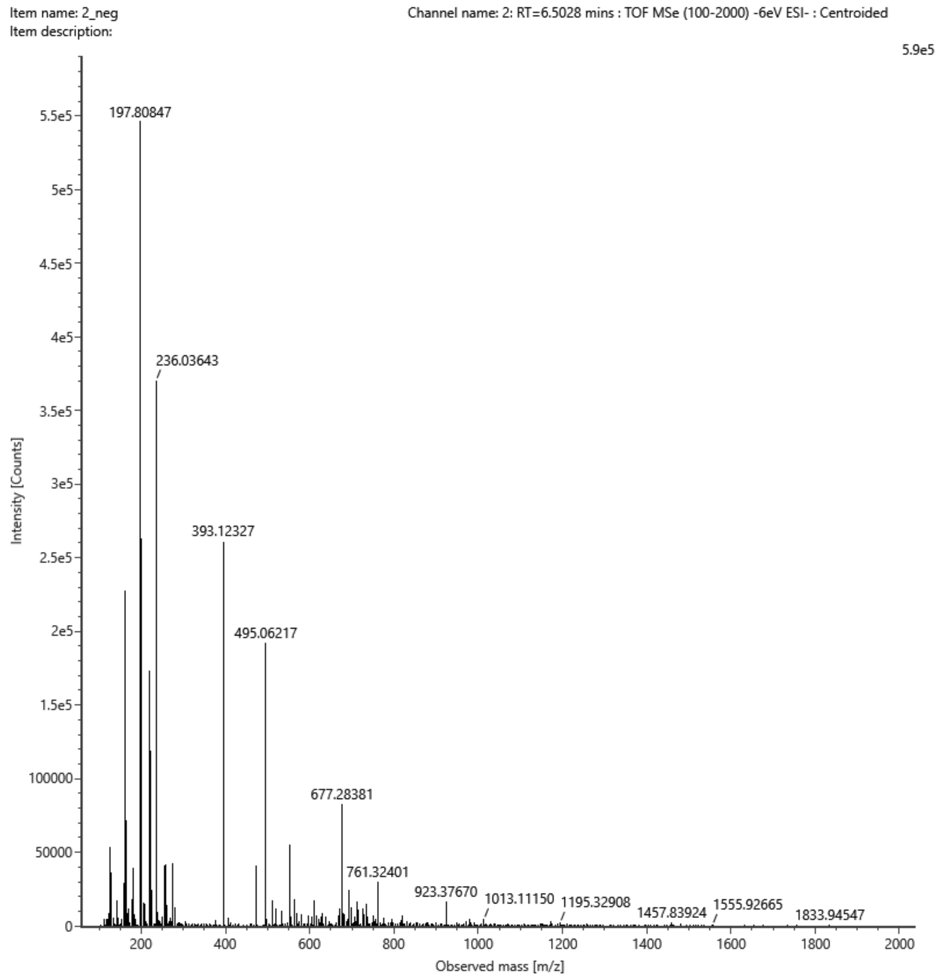

MS/MS spectrum for the compound NI9 (supposed NCC_678 phyllobilin isomer)
ESI-MS mode: negative, Unispray ion source

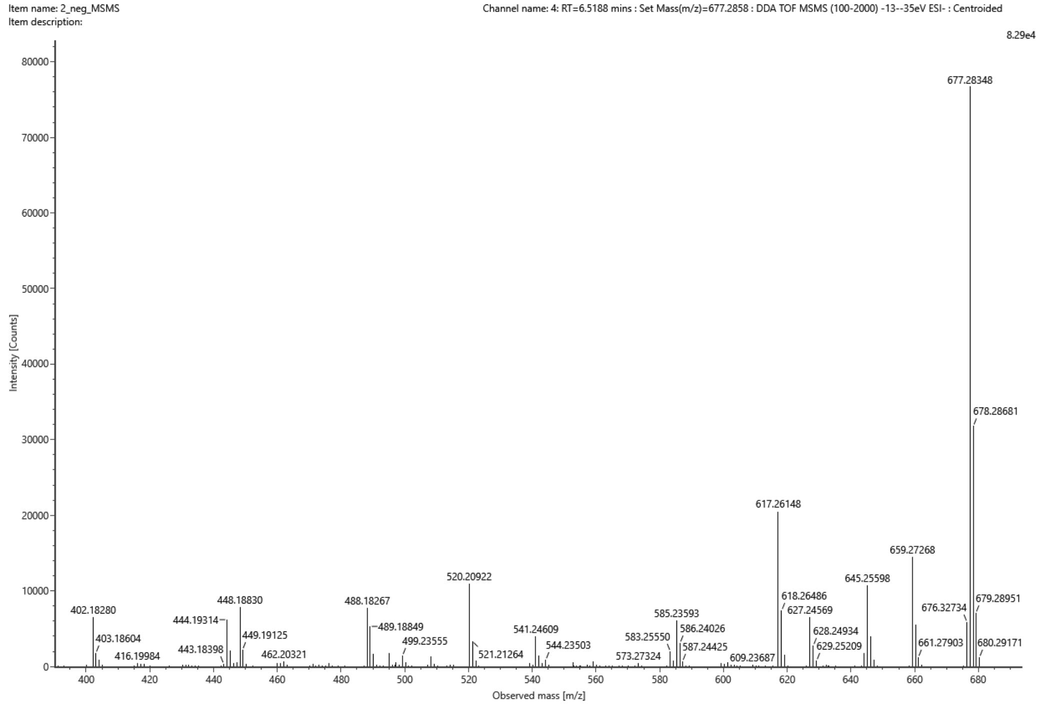

MS spectrum for the compound NI10 (supposed NCC_678 phyllobilin isomer)
ESI-MS mode: negative, Unispray ion source

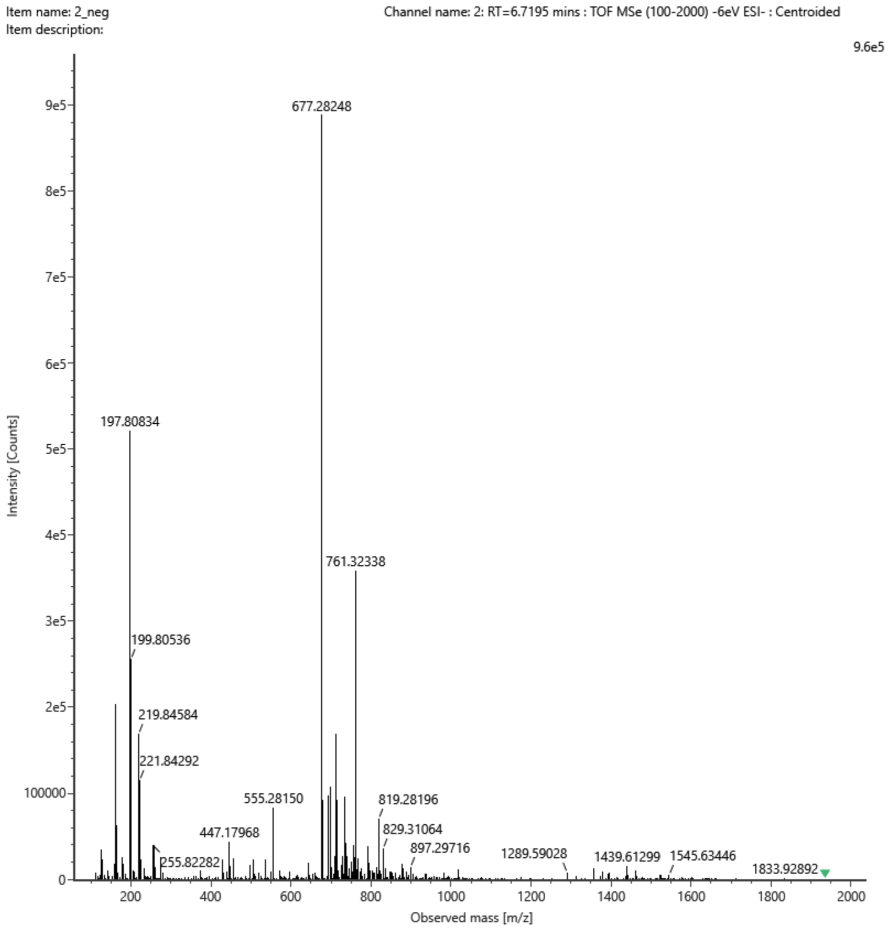

MS/MS spectrum for the compound NI10 (supposed NCC_678 phyllobilin isomer)
ESI-MS mode: negative, Unispray ion source

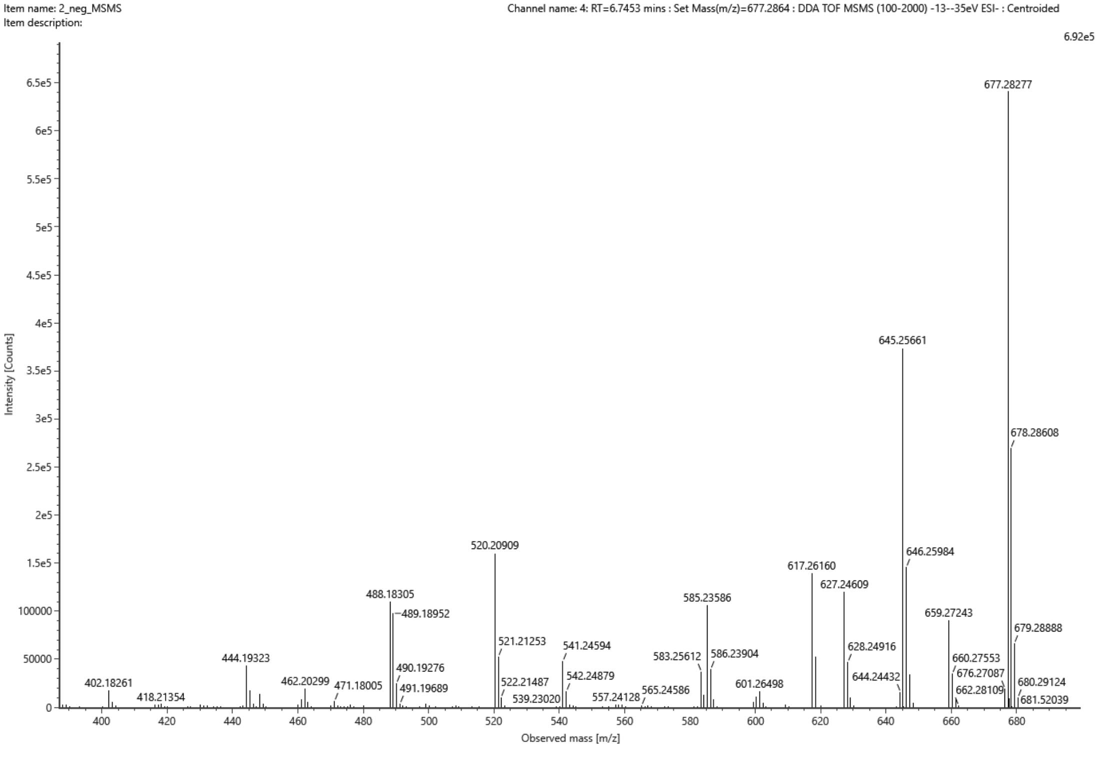

MS spectrum for the compound NI11 (supposed NCC_678 phyllobilin isomer)
ESI-MS mode: negative, Unispray ion source

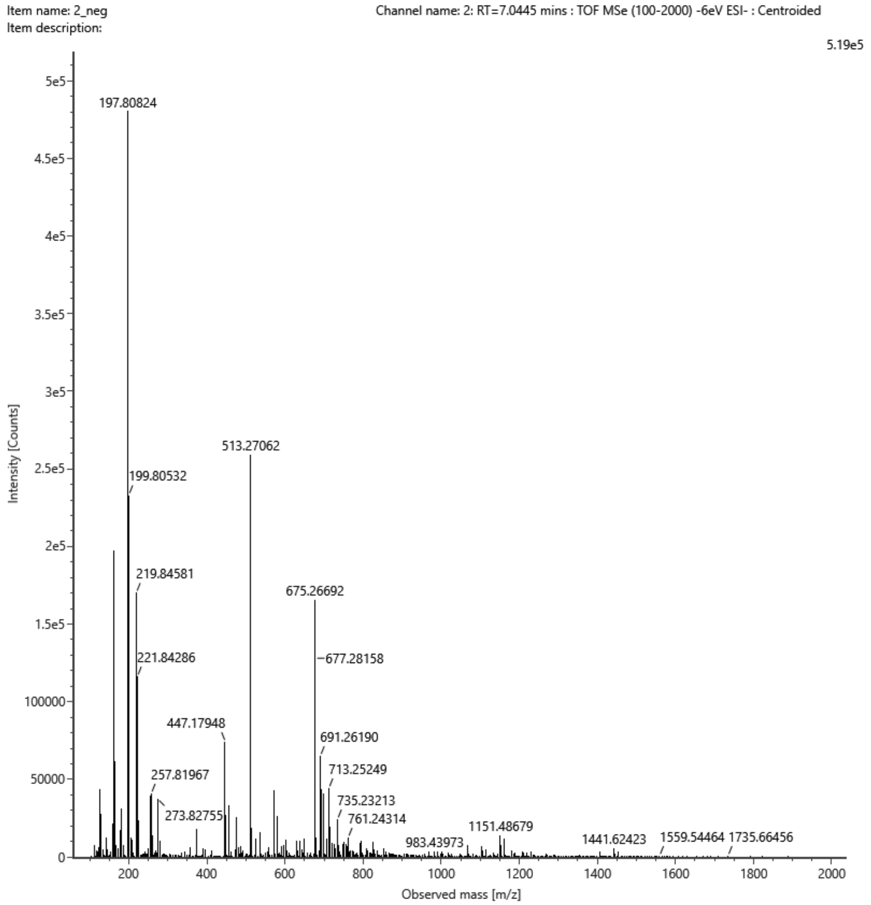

MS/MS spectrum for the compound NI11 (supposed NCC_678 phyllobilin isomer)
ESI-MS mode: negative, Unispray ion source

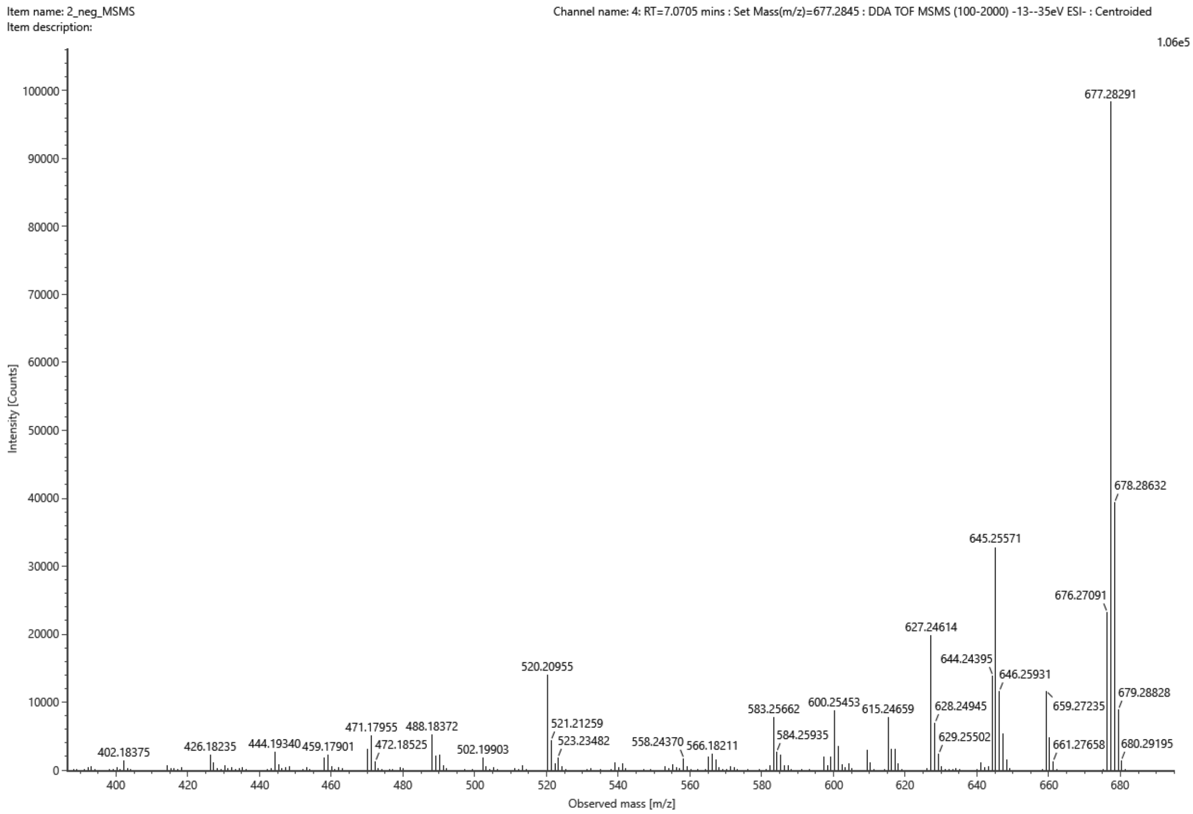

MS spectrum for the compound NI12 (supposed Capsianoside-IX)
ESI-MS mode: negative, Unispray ion source

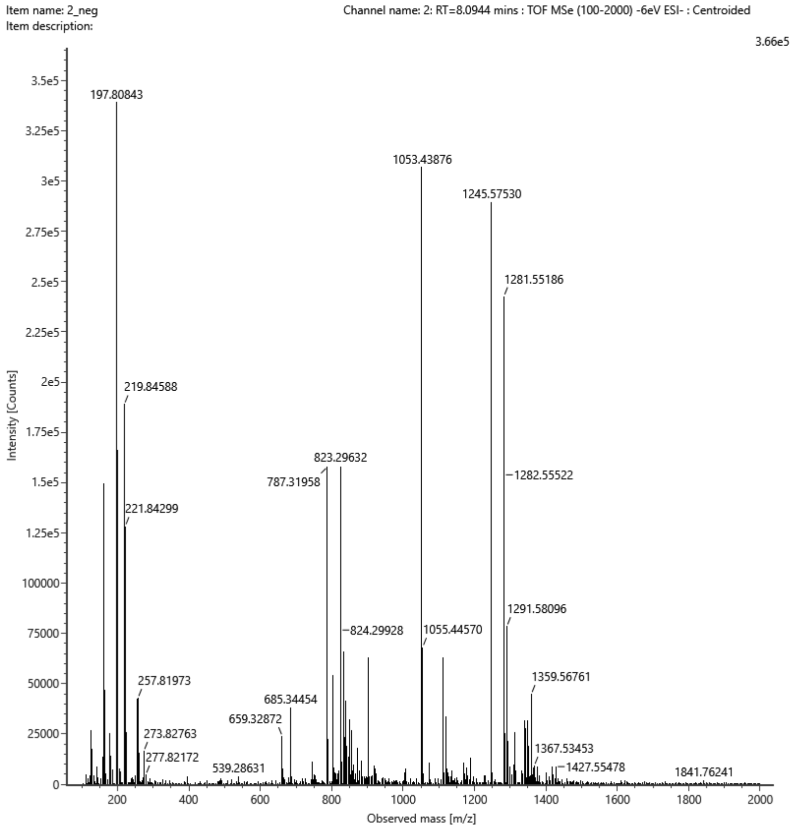

MS/MS spectrum for the compound NI12 (supposed Capsianoside-IX)
ESI-MS mode: negative, Unispray ion source

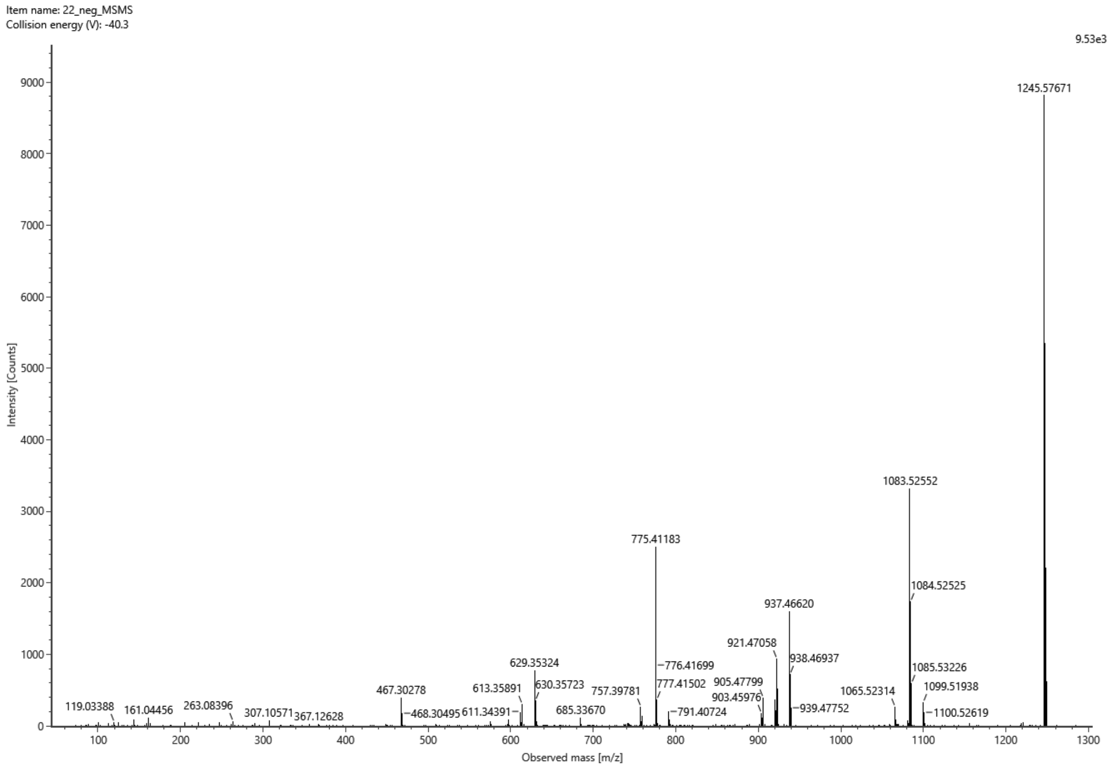

MS spectrum for the compound NI14 (supposed Capsianoside monomer)
ESI-MS mode: negative, Unispray ion source

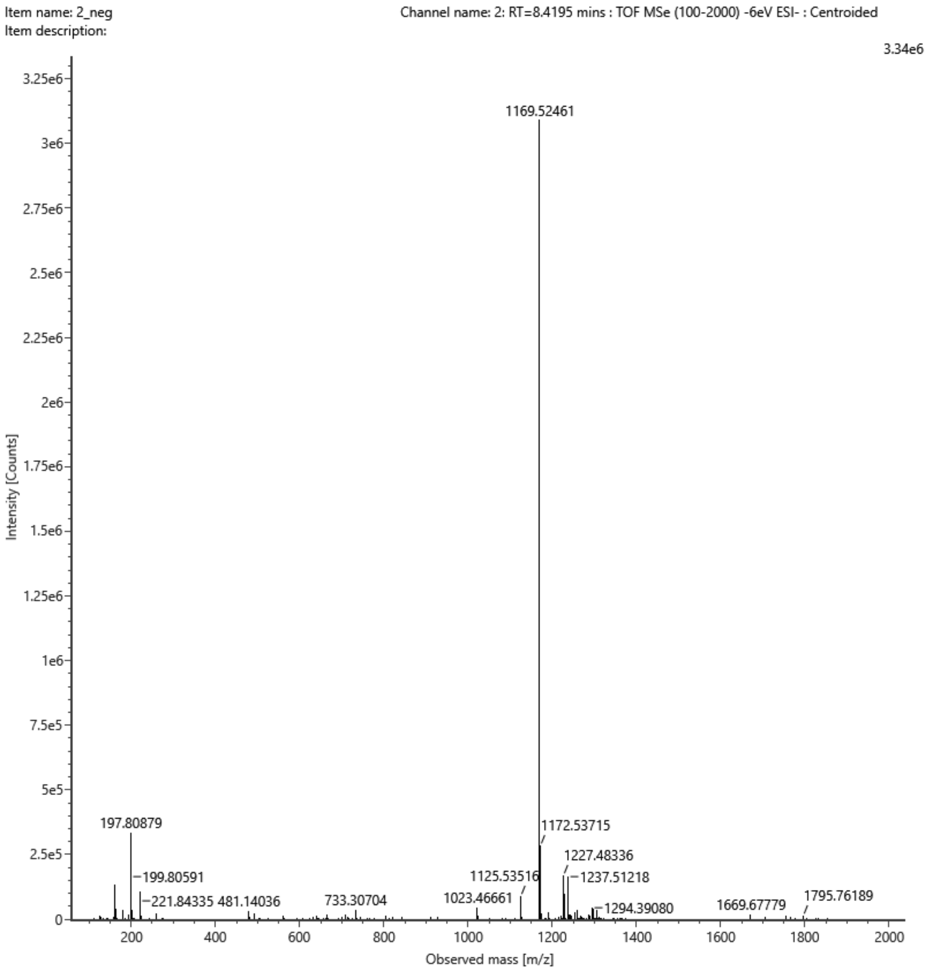

MS/MS spectrum for the compound NI14 (supposed Capsianoside monomer)
ESI-MS mode: negative, Unispray ion source

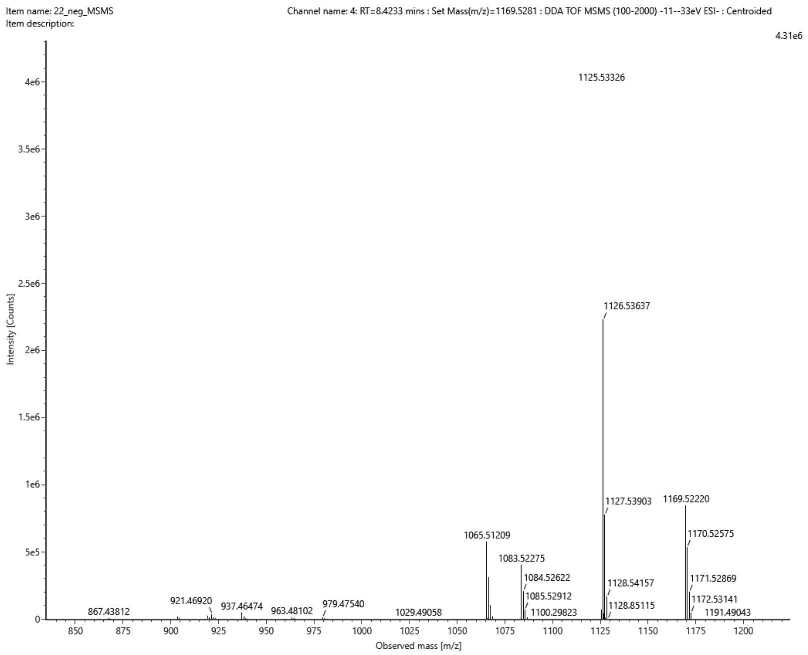

Supplement: Supplementary file 3 [file Data_Sheet_2.pdf]
