# Supplementary material for: Modulated Light Dependence of Growth, Flowering, and the Accumulation of Secondary Metabolites in Chilli
Source: Front Plant Sci. 2022 Mar 22;13:801656. doi: 10.3389/fpls.2022.801656 (PMC8981241; doi:10.3389/fpls.2022.801656)

Supplementary information for the article entitled

Modulated light dependence of growth, flowering and the accumulation of secondary metabolites in chilli

published in Frontiers in Plant Science
(section Crop and Product Physiology; manuscript No. 801656;
doi: 10.3389/fpls.2022.801656)

by

Eva Darko[1]*, Kamirán A. Hamow[1], Tihana Marcek[2], Mihály Dernovics[1], Mohamed Ahres[1] and Gábor Galiba[1,3]

[1] Centre for Agricultural Research, Agricultural Institute, Martonvásár, Hungary

[2] Faculty of Food Technology, Josip Juraj Strossmayer University of Osijek, Osijek, Croatia

[3] Hungarian University of Agriculture and Life Sciences, Keszthely, Hungary

*corresponding author

Supplementary Figure 1 : Schematic presentation of the light regimens (A – F). The plants were grown under 16/8 h photperiod under different light intensities and spectral combinations:

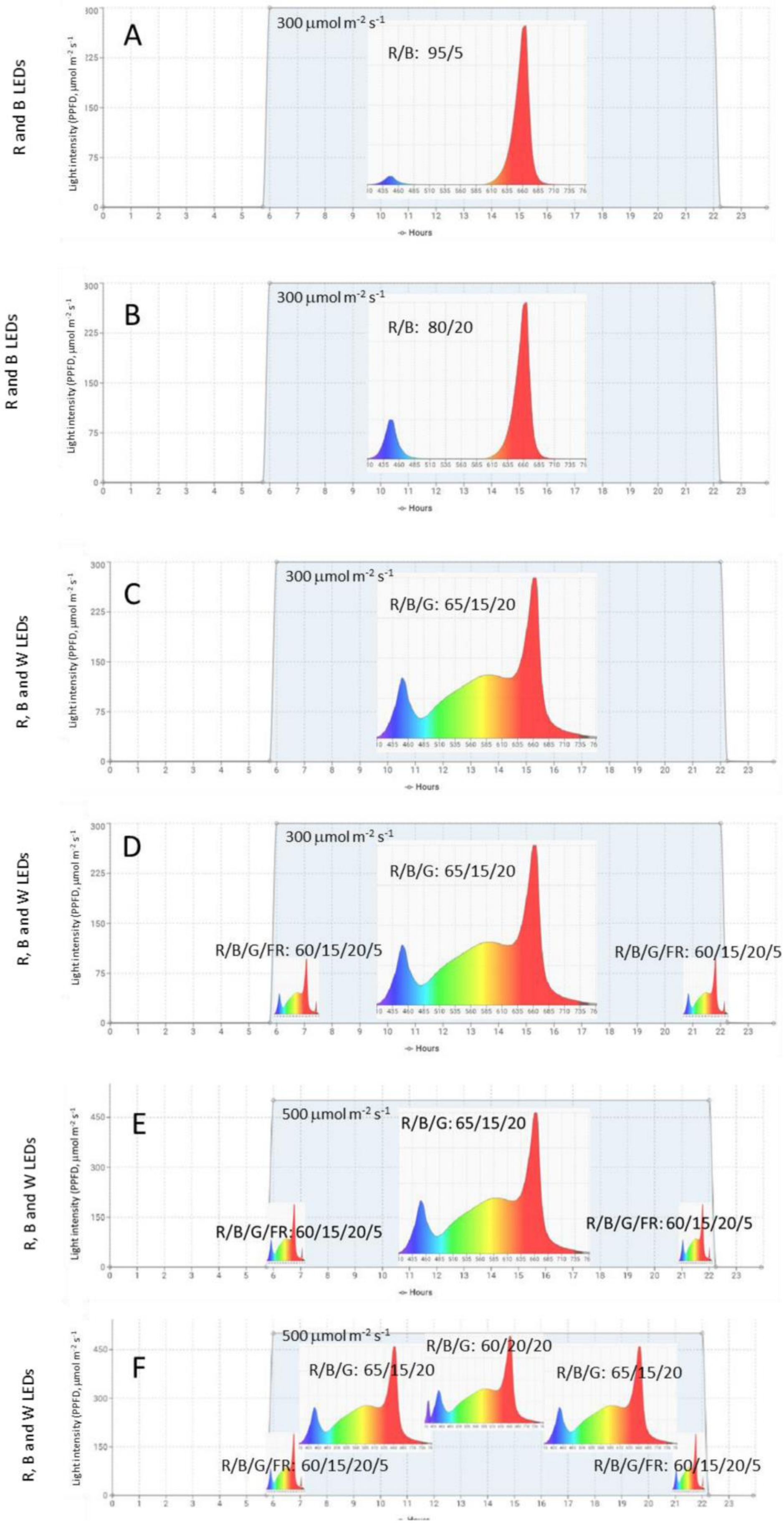

Supplement: Supplementary file 4 [file Image_1.pdf]
